# Selective Aerobic Peroxidation of Styrene Catalyzed by a Cobalt *tert*-Butylperoxo Complex

**DOI:** 10.1021/jacsau.5c00139

**Published:** 2025-02-28

**Authors:** Yunzhou Chen, Huiying Song, Yiming Hao, Matthew Y. Lui, Wing-Leung Wong, William W. Y. Lam, Bun Chan, Huatian Shi, Wai-Lun Man

**Affiliations:** † Department of Chemistry, Hong Kong Baptist University, Waterloo Road, Kowloon Tong, HKSAR 999077, PR China; ‡ Department of Applied Biology and Chemical Technology, The Hong Kong Polytechnic University, Hung Hom, Kowloon, HKSAR 999077, PR China; § Department of Food and Health Sciences, Technological and Higher Education Institute of Hong Kong, Tsing Yi, New Territories, HKSAR 999077, PR China; ∥ Graduate School of Engineering, Nagasaki University, Bunkyo 1-14, Nagasaki 852-8521, Japan; ⊥ School of Environment and Civil Engineering, Research Center for Eco-environmental Engineering, Dongguan University of Technology, Dongguan, Guangdong 523808, PR China

**Keywords:** Cobalt catalysis, alkylperoxo complex, styrene oxidation, aerobic peroxidation, mechanism

## Abstract

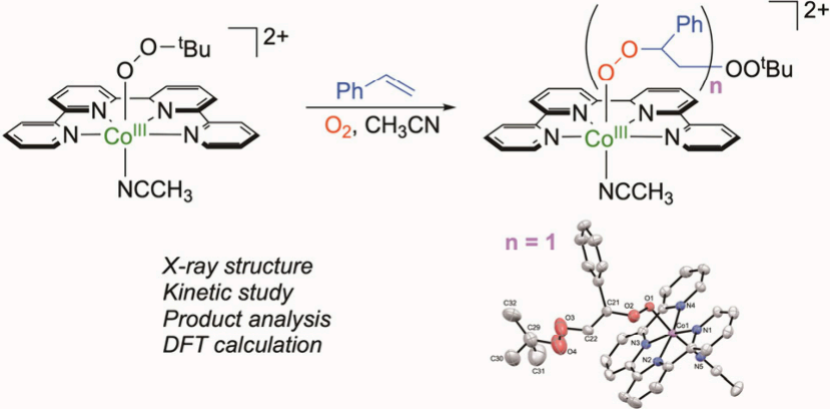

Selective oxidation of styrene to desired products is essential and challenging. In this study, we elucidate a unique pathway for the selective oxidation of styrene to polystyrene peroxo species, catalyzed by the cobalt(III) *tert*-butylperoxo complex, [Co^III^(OO^*t*^Bu)(qpy)(NCCH_3_)]^2+^ (**1**), under ambient conditions. Mechanistic investigations, including the structural determination of the diperoxo complex, [Co^III^(qpy)(OOCH(Ph)CH_2_OO^*t*^Bu)(NCCH_3_)]^2+^ (**2**), by X-ray analysis and theoretical calculations reveal that the reaction begins with the nucleophilic addition of styrene to the Co^III^–OO^*t*^Bu moiety in **1**. This step is followed by an addition with an O_2_ molecule, forming a diperoxyl radical (PhCOO^•^(H)CH_2_OO^t^Bu), which subsequently rebounds with Co^II^(qpy) to yield **2**. In the presence of excess O_2_, complex **2** can further react with additional styrene molecules, leading to the formation of cobalt(III) polystyrene peroxo species.

Selective oxidation of styrene under various conditions to give products such as ketones, epoxides, diols, and diperoxides is an important reaction in the chemical laboratory and industry, as the various oxidized products are important intermediates for synthesizing various useful chemicals. While metal-catalyzed selective oxygenation of styrene (Scheme 1) to carbonyls through C=C cleavage (path a),^1−6^ epoxides via epoxidation (path b),^7−14^ and 1,2-diols through dihydroxylation (path c)^15−20^ has been extensively studied, the selective oxidation to diperoxides (path d)^21−25^ is underexplored. Peroxo species are potential compounds with diverse applications. They have been used as fuel substitutes, biocompatible drug carriers, and coating materials.^26^ Alkylperoxo complexes, particularly of 3*d* metals, play important roles in organic molecule transformation.^27−31^ Recently, we reported the unique reactivity of the cobalt(III) *tert*-butylperoxo complex, [Co^III^(qpy)(OO^*t*^Bu)(NCCH_3_)]^2+^ (**1**) (qpy = 2,2′,6′,6″-quaterpyridine) in activating hydrocarbons (RH) with weak to mild C–H bonds under an ambient condition.^32^ For example, **1** catalyzed the aerobic allylic peroxidation of cyclopentene via an initial C–H activation with the release of ^*t*^BuOOH, as a free hydroperoxide (Scheme 2A).

**Scheme 1:**
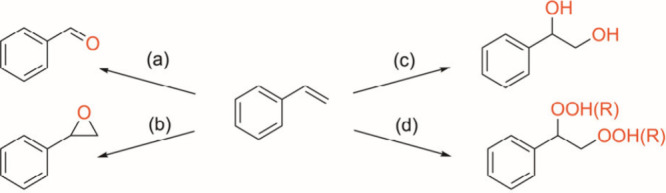
Metal-Catalyzed Oxidation of Styrene

**Scheme 2:**
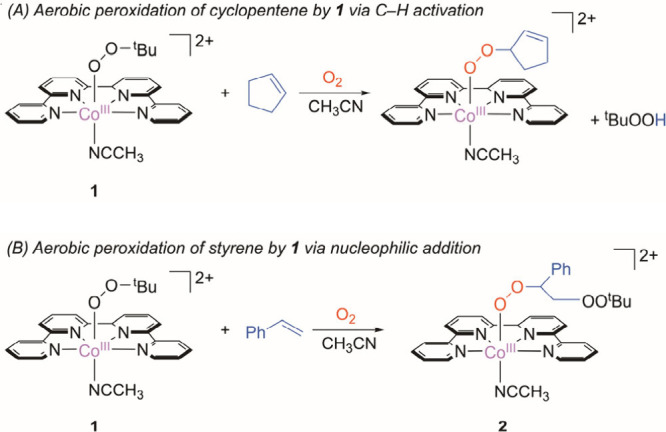
Aerobic Peroxidation of Hydrocarbons by **1**. (A) Cyclopentene. (B) Styrene

Herein, we report another unique reactivity of **1** toward styrene that does not contain weak C–H bonds (Scheme 2B). In the presence of excess O_2_, **1** facilitates catalytic aerobic peroxidation of styrene via an initial nucleophilic addition of styrene to generate polystyrene peroxo species selectively over other oxygenated products such as carbonyls, epoxides, and diols.

Treatment of **1** with excess styrene (PhCH=CH_2_) in CH_3_CN for 8 h under a limited supply of air (reaction flask stoppered) at room temperature afforded the diperoxo complex, [Co^III^(qpy)(OOCH(Ph)CH_2_OO^*t*^Bu)(NCCH_3_)]^2+^ (**2**) with 77% isolated yield. The molecular structure of **2** (Figure 1) shows a styrene peroxo moiety inserted into the Co–OO^*t*^Bu bond of **1**. The Co–O and Co–N bond distances and the Co–O–O bond angle in **2** are similar to **1** (Table S1 and S2). Mechanistic and theoretical studies below suggest that the styrene peroxo moiety is formed from “PhCH=CH_2_ + exogenous O_2_”.

**Figure 1:**
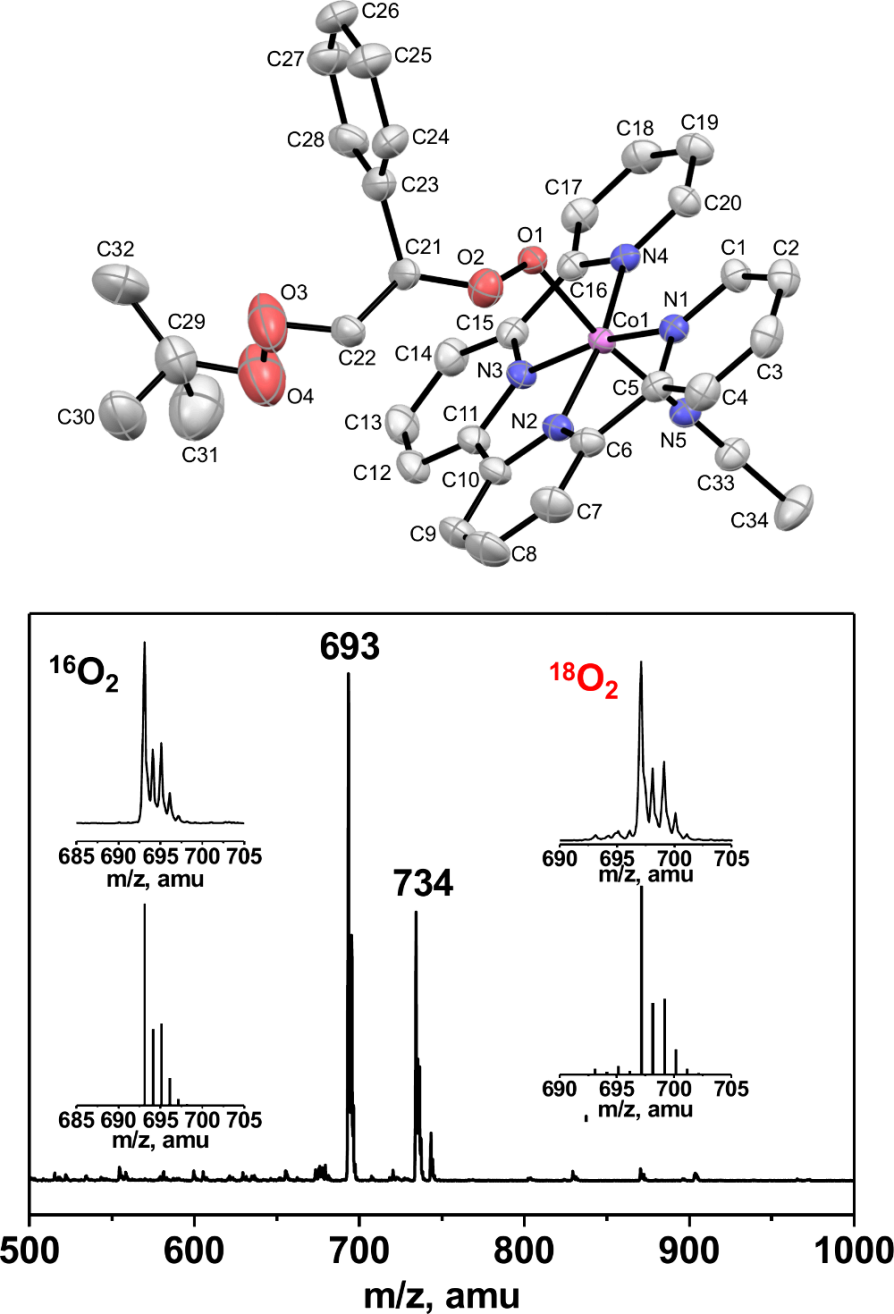
X-ray structure (top) and ESI mass spectrum (bottom) of **2**. Insets show the experimental (top) and simulated (bottom) patterns for the dominant peaks of **2** (left) and ^**18**^**O**_**2**_**-2** (right).

The electrospray ionization (ESI) mass spectrum (positive mode in CH_3_CN) of **2** displays two prominent peaks at *m*/*z* 734 and 693, corresponding to the singly charged parent ion, {[Co(qpy)(OOCH(Ph)CH_2_OO^*t*^Bu)(NCCH_3_)](ClO_4_)}^+^ (M^+^) and its fragment ion (M^+^ – CH_3_CN), respectively (Figure 1). The ^**18**^**O**_**2**_**-2** complex (prepared using 98% ^18^O-enriched O_2_) exhibits a 4-mass unit shift of the fragment ion to *m*/*z* 697, indicating that the two oxygen atoms of the styrene peroxo moiety come from O_2_ in the air. The ^1^H NMR spectrum of **2** shows peaks arising from PhCHCH_2_, including five aromatic protons (δ 6.46–7.20 ppm) and two multiplets at δ 3.5 and 3.7 ppm corresponding to the C*H* and C*H*_2_ protons, respectively (Figure S1). Notably, the ^*t*^Bu singlet shifts significantly from δ 0.35 ppm in **1** to δ 0.96 ppm in **2**, indicating decreased magnetic interaction between the ^*t*^Bu group and the aromatic qpy rings due to their increased separation.^33^

The kinetics of the reaction between 0.2 mM **1** and 0.2 M styrene were investigated in an air-saturated CH_3_CN solution. The UV–vis spectral changes reveal a gradual conversion from **1** (red line) to **2** (blue line), with isosbestic points maintained at 447, 512, and 619 nm throughout the reaction (Figure 2A). The decay of **1** at 686 nm follows pseudo-first-order kinetics over three half-lives. The pseudo-first-order rate constant, *k*_obs_, shows a linear correlation with [styrene] and gives a second-order rate constant, *k*_2_, of (4.64 ± 0.10) × 10^–3^ M^–1^ s^–1^ at 25 °C (Figure S2). When **1** (0.2 mM) was mixed with styrene (0.2 M) under an O_2_-saturated atmosphere, a similar *k*_obs_ value of 1.23 × 10^–3^ s^–1^ was determined (Figure S3). The effect of temperature on the reaction rates was studied between 10–40 °C. A reasonably linear correlation was found in the Eyring plot of ln(*k*_2_/T) versus 1/T (Figures 2B and S2, and Table S3). Activation parameters, including the enthalpy (Δ*H*^‡^) = (23.4 ± 0.8) *k*cal mol^–1^ and the entropy (Δ*S*^‡^) = (10 ± 3) cal mol^–1^ K^–1^, were determined from the slope and the y-intercept of the plot, respectively. At 25 °C, the Gibbs free energy (Δ*G*^‡^) of (20.4 ± 1.0) *k*cal mol^–1^ was calculated. The kinetics of the reaction of **1** with various *para*-substituted styrenes (*p*-X-PhCH=CH_2_) were also investigated (Table S3 and Figure S4) and resulted in a linear Hammett correlation between log(*k*_2_^X^/*k*_2_^H^) and the Hammett constants (σ_*p*_). The small negative ρ value of −0.79 was determined from the slope, indicating a moderate electrophilic reactivity of **1** toward styrenes (Figure 2C).

**Figure 2:**
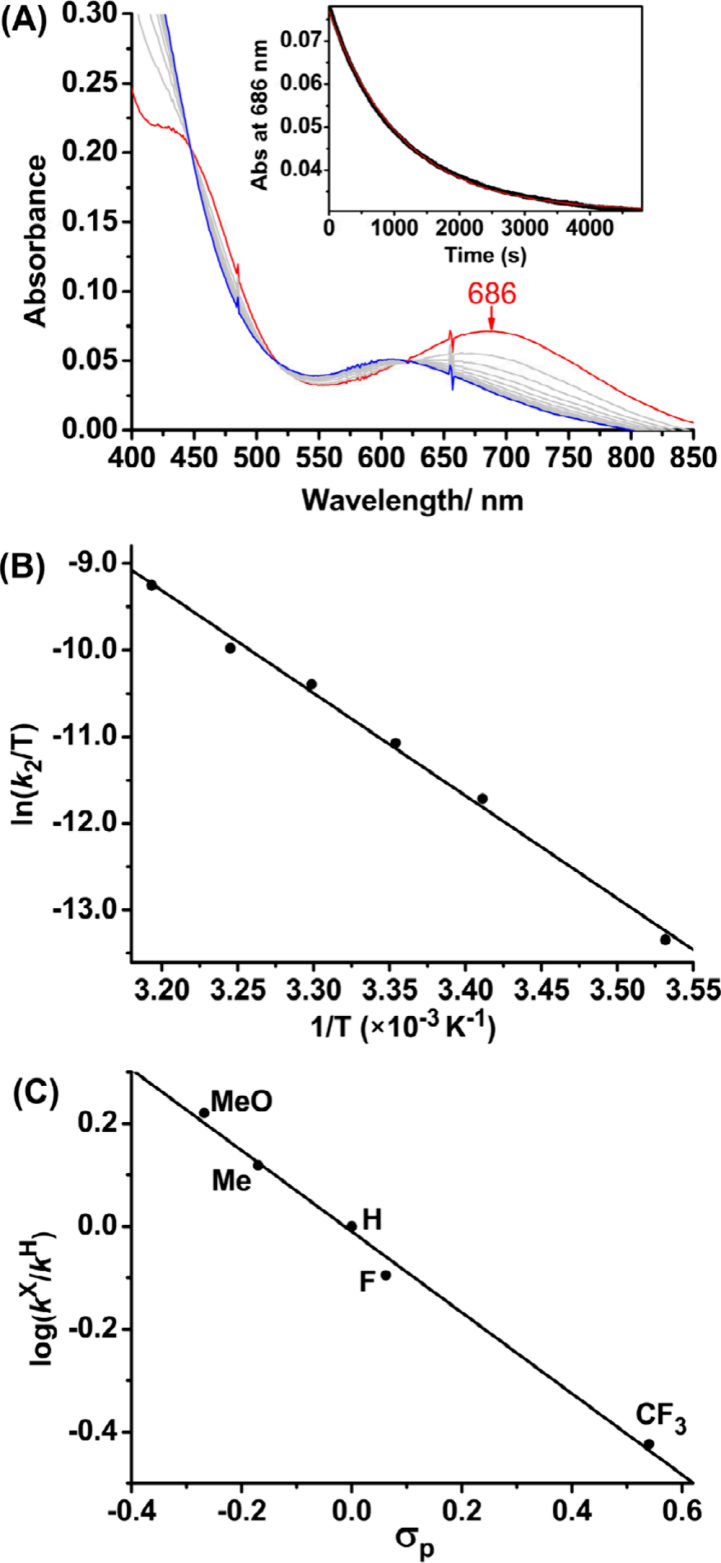
Kinetic studies by UV–vis. (A) Spectral changes at 300 s intervals for the reaction of **1** (0.2 mM) and styrene (0.2 M) in CH_3_CN in air at 25 °C. Inset shows the decay of **1**. (B) Plot of ln(*k*_2_/T) against 1/T. (C) Plot of log(*k*^X^/*k*^H^) against σ_*p*_.

The kinetic studies described above for converting **1** to **2** were carried out in limited [O_2_] (ca. 1.6 mM in CH_3_CN at 25 °C).^34^ When the reaction was carried out under continuous exposure to air, further reaction occurred, the nature of which was revealed by ESI/MS and ^1^H NMR. Upon stirring 0.2 mM **1** and 0.2 M styrene in CH_3_CN under air at 23 °C, the MS indicates almost complete disappearance of **1** (*m*/*z* 557) and the formation of **2** (*m*/*z* 693) as the predominant species after 15 min (Figure 3). The mass difference of 136 amu between **1** and **2** is attributed to incorporating the “*PhCHCH*_2_*+ O*_2_^”^ adduct. Intriguingly, two distinct (*PhCHCH*_2_*+ O*_2_)_*n*_ adducts are also observed at *m*/*z* 829 (*n* = 2) and 965 (*n* = 3). The number of adducts (*n*) increased with time, reaching a maximum of *n* = 6 (*m*/*z* 1373) after 10 h, suggesting that **1** catalyzed the aerobic peroxidation of styrene to generate oligomeric styrene peroxo complexes. It is noteworthy that a minor and unidentified peak at *m*/*z* 929 was also formed after 4 h during the oligoperoxidation process, suggesting a possible side reaction.

**Figure 3:**
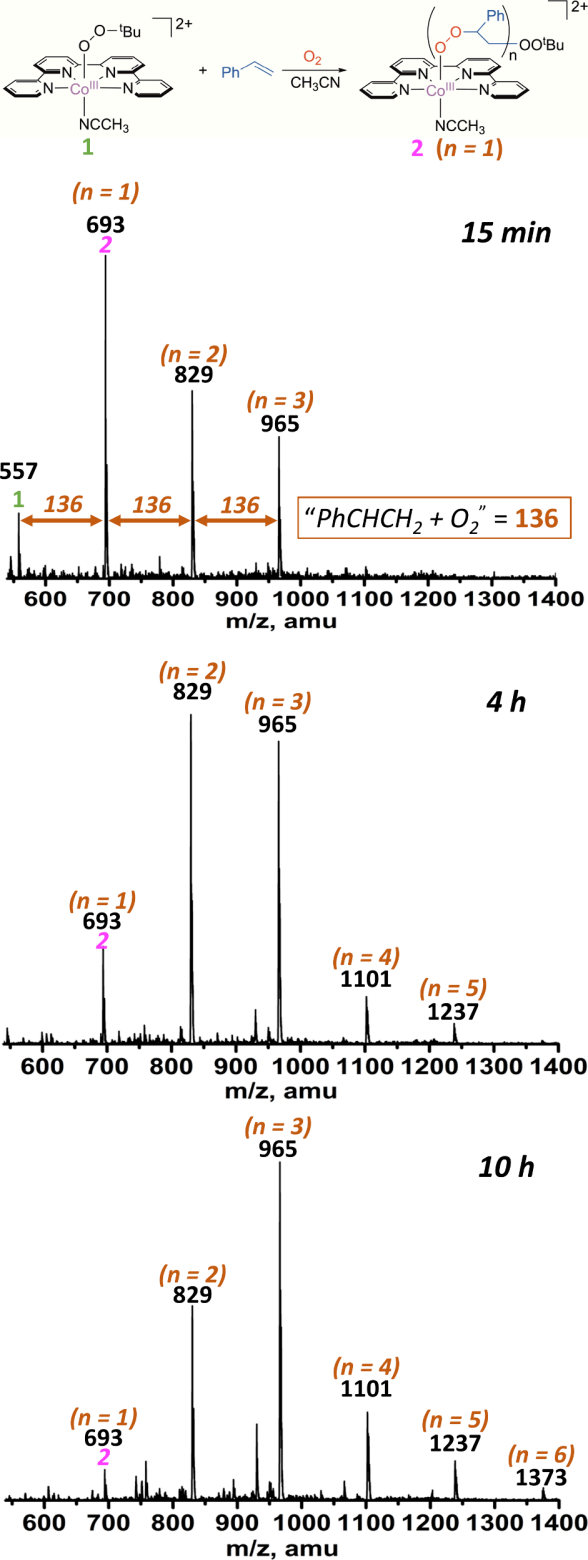
Product analysis at different time intervals for the reaction of **1** (0.2 mM) and styrene (0.2 M) in CH_3_CN by ESIMS under ambient conditions. The peaks correspond to the ions with the general formula {[Co(qpy)(OOCH(Ph)CH_2_)_n_OO^*t*^Bu](ClO_4_)}^+^.

If the solution containing 0.2 mM **1** and 1 M styrene was stirred under an O_2_-saturated atmosphere, a maximum of n = 13 (*m*/*z* 2461) was achieved after 3 h (Figure S5). This oligoperoxidation process is further supported by ^1^H NMR (Figure S6). In the absence of O_2_, UV–vis analysis indicated a reduction of Co(III) to Co(II), with no absorption peak observed between 600 and 700 nm (Figure S2). ESIMS did not detect any diperoxo or oligoperoxo species; instead, styrene oxide and ^*t*^BuOH were identified by NMR and GC-MS (Figure S7).

To provide further insight into the reaction mechanism of **1** with styrene in the presence of air, we conducted a computational analysis at the B3LYP/def2-TZVP level of theory (Scheme 3). All calculated structures at different spin states are compiled in Figures S8–35. The discussion focuses mainly on the species with the lowest energy in the potential energy surface. First of all, the ground state of **1** was confirmed at the singlet state with an energy of 4.6 *k*cal mol^–1^ lower than the low-lying quintet state, which is consistent with the diamagnetism of **1** in CD_3_CN. Hence, the sum of the energy of **1** at the singlet state and styrene (**I**) was used as a reference. When **1** and styrene were placed together, a reaction complex (**II**) was achieved at 6.2 *k*cal mol^–1^ at the singlet state. The PhCH=CH_2_ of **II** then undergoes a nucleophilic addition to **1** (via the terminal carbon atom onto the proximal oxygen atom of Co^III^–*O*O^t^Bu) to generate a weakly polar transition state (**III**), collaborated with the small negative ρ value (−0.79) obtained from the Hammett plot (Figure 2C). The energy of **III** at the singlet state is 23.1 *k*cal mol^–1^. On the other hand, the lower energy of 21.7 *k*cal mol^–1^ at the quintet state (−1.4 *k*cal mol^–1^) suggests a possible spin state crossover from singlet **II** to quintet **III**. Nevertheless, both comparable values at the singlet and quintet states are consistent with the experimental Δ*G*^‡^ value of (20.4 ± 1.0) *k*cal mol^–1^. At the quintet transition state **III**, the Co–O bond distance increased from 1.85 to 2.19 Å, and the spin densities were observed at the two oxygen atoms (OO^*t*^Bu moiety) and the internal carbon atom of styrene (Figure S19). Rearrangement of electrons afforded the intermediate (**IV**) results in a C–O bond formation (via the terminal C atom of styrene and the proximal O atom of *O*O^*t*^Bu) with the energies of all spin states within ±1 *k*cal mol^–1^ (from the lowest 10.2 *k*cal mol^–1^ of the broken-symmetry triplet to the highest 11.2 *k*cal mol^–1^ of the singlet state). The molecular orbital and spin density analyses suggest the formulation of **IV** as a Co^II^(qpy) complex with a weakly O-bound carbon-centered radical (PhC^•^(H)CH_2_OO^*t*^Bu). This reactive alkyl radical captures an O_2_ molecule at a diffusion-controlled rate,^35^ leading to the formation of a diperoxyl radical in **V**, for which several spin states are close in energy. Finally, the diperoxyl radical couples with Co^II^(qpy) to yield the close-shell cobalt(III) diperoxyl complex **2** (**VI**) from the preceding singlet, which is the second-lowest-energy spin state of **V**. Overall, the reaction is thermodynamically favorable by lowering the energy of 4.4 *k*cal mol^–1^. It is noteworthy that the formation of an alternative carbon-bound intermediate, Co–C(Ph)HCH_2_O_2_^*t*^Bu, after **III,** is also possible. Although the energies of these species in singlet (6.1 *k*cal mol^–1^), triplet (9.7 *k*cal mol^–1^), and quintet states (9.1 *k*cal mol^–1^), are slightly lower than that of **IV** in 10.2 *k*cal mol^–1^, this organometallic intermediate is less likely to occur after considering the experimental results. In the absence of O_2_, we detected styrene oxide and ^*t*^BuOH by NMR and GCMS. It is proposed that the (PhC^•^(H)CH_2_OO^*t*^Bu) radical (**IV**) would undergo homolytic O–O cleavage, followed by the intramolecular coupling of diradicals to generate styrene oxide. The highly reactive ^*t*^BuO^•^ could abstract a hydrogen atom, giving rise to ^*t*^BuOH. **2** may further react with a second styrene molecule via a similar mechanism, which in the presence of excess O_2_ would eventually lead to polymeric styrene peroxo species. In principle, the radical intermediates in **IV** and **V** can also initiate a radical chain reaction to activate other styrene molecules to form polymeric styrene peroxo radicals before being scavenged by Co^II^(qpy). However, this radical chain reaction is not predominant; both **IV** to **V** and **V** to **VI** conversions are downhill reactions. More significantly, **2** has been isolated with a 77% yield during the synthesis. Our previous study also demonstrated that recombining Co^II^(qpy) species and the alkylperoxo radical is a fast reaction.^32^

**Scheme 3:**
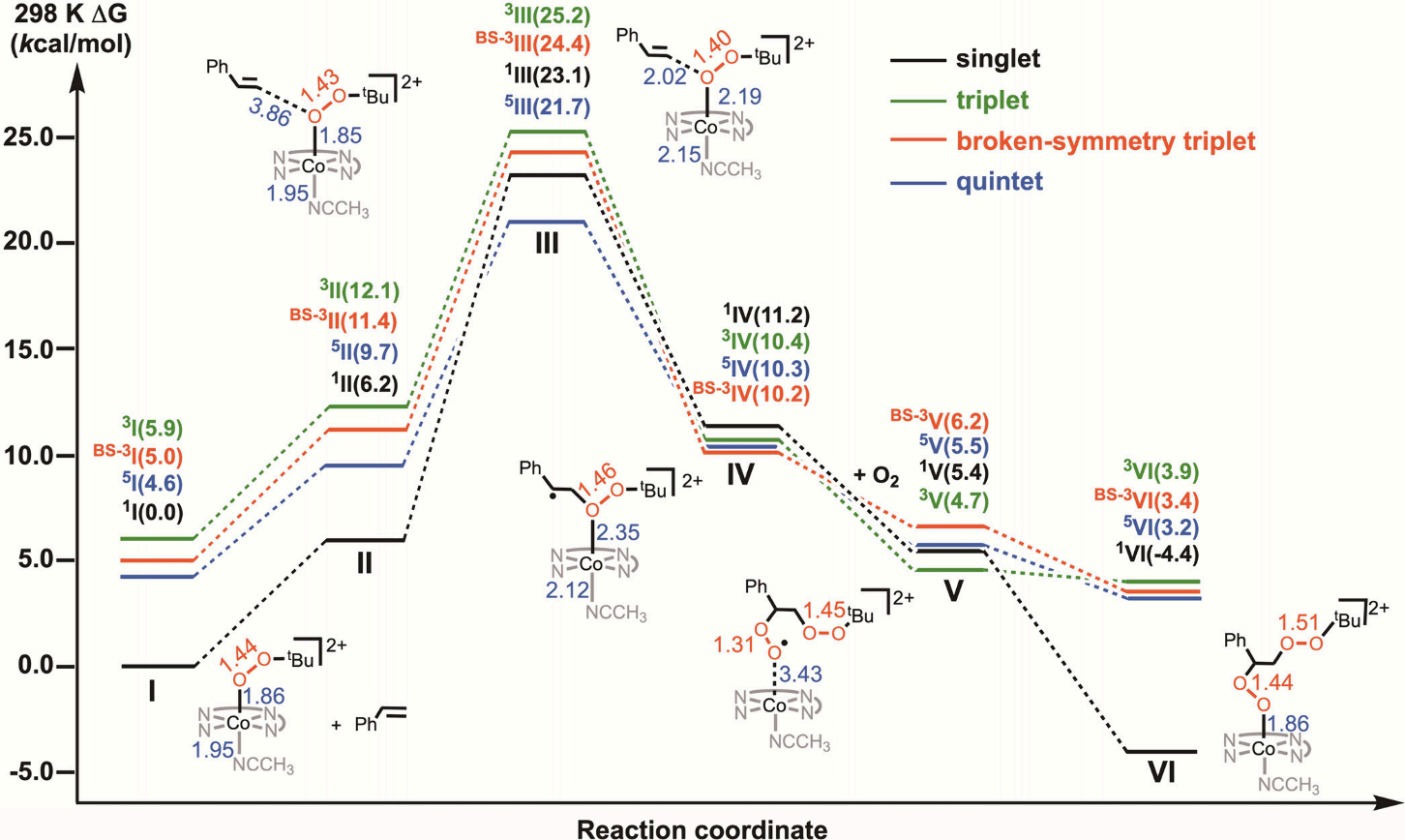
Energy Profile for the Reaction of **1** and Styrene in the Presence of O_2_ in CH_3_CN from DFT Calculation at the B3LYP/def2-TZVP Level Selected bond distances are given in Å.

In summary, we have provided experimental evidence for the selective aerobic peroxidation of styrene by **1** to generate a diperoxo complex **2** under ambient conditions. Experimental studies and theoretical calculations suggest that the mechanism involves the initial nucleophilic addition of styrene to **1** followed by the addition of O_2_, resulting in the peroxidation of styrene, which remains bound to the cobalt center. In the presence of excess O_2_, catalytic peroxidation of styrene occurs to generate polystyrene peroxo species, which are promising candidates for various applications.

## Supporting Information Available

Crystallographic data (CIF)
Detailed experimental procedures, spectroscopic and crystallographic data, and computational studies (PDF)

## Supplementary Material

au5c00139_si_001.cif

## References

[ref1] Abuhafez N.; Ehlers A. W.; de Bruin B.; Gramage-Doria R. Markovnikov-Selective Cobalt-Catalyzed Wacker-Type Oxidation of Styrenes into Ketones under Ambient Conditions Enabled by Hydrogen Bonding. Angew. Chem., Int. Ed. 2024, 63, e202316825 10.1002/anie.202316825. 38037901

[ref2] Liang Y.-F.; Bilal M.; Tang L.-Y.; Wang T.-Z.; Guan Y.-Q.; Cheng Z.; Zhu M.; Wei J.; Jiao N. Carbon-Carbon Bond Cleavage for Late-Stage Functionalization. Chem. Rev. 2023, 123, 12313–12370. 10.1021/acs.chemrev.3c00219. 37942891

[ref3] Huang Z.; Guan R.; Shanmugam M.; Bennett E. L.; Robertson C. M.; Brookfield A.; McInnes E. J. L.; Xiao J. Oxidative Cleavage of Alkenes by O2 with a Non-Heme Manganese Catalyst. J. Am. Chem. Soc. 2021, 143, 10005–10013. 10.1021/jacs.1c05757. 34160220 PMC8297864

[ref4] Salzmann K.; Segarra C.; Albrecht M. Donor-Flexible Bis(pyridylidene amide) Ligands for Highly Efficient Ruthenium-Catalyzed Olefin Oxidation. Angew. Chem., Int. Ed. 2020, 59, 8932–8936. 10.1002/anie.202002014. 32100371

[ref5] Urgoitia G.; SanMartin R.; Herrero M. T.; Dominguez E. Aerobic Cleavage of Alkenes and Alkynes into Carbonyl and Carboxyl Compounds. ACS Catal. 2017, 7, 3050–3060. 10.1021/acscatal.6b03654.

[ref6] Daw P.; Petakamsetty R.; Sarbajna A.; Laha S.; Ramapanicker R.; Bera J. K. A Highly Efficient Catalyst for Selective Oxidative Scission of Olefins to Aldehydes: Abnormal-NHC-Ru(II) Complex in Oxidation Chemistry. J. Am. Chem. Soc. 2014, 136, 13987–13990. 10.1021/ja5075294. 25237828

[ref7] Cao Q.; Diefenbach M.; Maguire C.; Krewald V.; Muldoon M. J.; Hintermair U. Water Co-Catalysis in Aerobic Olefin Epoxidation Mediated by Ruthenium Oxo Complexes. Chem. Sci. 2024, 15, 3104–3115. 10.1039/D3SC05516G. 38425537 PMC10901482

[ref8] Verspeek D.; Ahrens S.; Spannenberg A.; Wen X.; Yang Y.; Li Y.-W.; Junge K.; Beller M. Manganese N,N,N-pincer Complex-Catalyzed Epoxidation of Unactivated Aliphatic Olefins. Catal. Sci. Technol. 2022, 12, 7341–7348. 10.1039/D2CY01472F.

[ref9] Vicens L.; Olivo G.; Costas M. Rational Design of Bioinspired Catalysts for Selective Oxidations. ACS Catal. 2020, 10, 8611–8631. 10.1021/acscatal.0c02073.

[ref10] Shing K.-P.; Cao B.; Liu Y.; Lee H. K.; Li M.-D.; Phillips D. L.; Chang X.-Y.; Che C.-M. Arylruthenium(III) Porphyrin-Catalyzed C–H Oxidation and Epoxidation at Room Temperature and [Ru^V^(Por)(O)(Ph)] Intermediate by Spectroscopic Analysis and Density Functional Theory Calculations. J. Am. Chem. Soc. 2018, 140 (22), 7032–7042. 10.1021/jacs.8b04470. 29781605

[ref11] Miao C.; Wang B.; Wang Y.; Xia C.; Lee Y.-M.; Nam W.; Sun W. Proton-Promoted and Anion-Enhanced Epoxidation of Olefins by Hydrogen Peroxide in the Presence of Nonheme Manganese Catalysts. J. Am. Chem. Soc. 2016, 138, 936–943. 10.1021/jacs.5b11579. 26720313

[ref12] Cussó O.; Garcia-Bosch I.; Font D.; Ribas X.; Lloret-Fillol J.; Costas M. Highly Stereoselective Epoxidation with H_2_O_2_ Catalyzed by Electron-Rich Aminopyridine Manganese Catalysts. Org. Lett. 2013, 15, 6158–6161. 10.1021/ol403018x. 24245504

[ref13] Lane B. S.; Burgess K. Metal-Catalyzed Epoxidations of Alkenes with Hydrogen Peroxide. Chem. Rev. 2003, 103, 2457–2474. 10.1021/cr020471z. 12848577

[ref14] Al-Ajlouni A. M.; Espenson J. H. Epoxidation of Styrenes by Hydrogen Peroxide As Catalyzed by Methylrhenium Trioxide. J. Am. Chem. Soc. 1995, 117, 9243–9250. 10.1021/ja00141a016.

[ref15] Choukairi Afailal N.; Borrell M.; Cianfanelli M.; Costas M. Dearomative *syn*-Dihydroxylation of Naphthalenes with a Biomimetic Iron Catalyst. J. Am. Chem. Soc. 2024, 146, 240–249. 10.1021/jacs.3c08565. 38123164 PMC10785824

[ref16] Chen J.; Song W.; Lee Y. – M.; Nam W.; Wang B. Biological Inspired Nonheme Iron Complex-Catalyzed *cis*-Dihydroxylation of Alkenes Modeling Rieske Dioxygenases. Coord. Chem. Rev. 2023, 477, 214945 10.1016/j.ccr.2022.214945.

[ref17] Chen J.; Zhang J.; Sun Y.; Xu Y.; Yang Y.; Lee Y.-M.; Ji W.; Wang B.; Nam W.; Wang B. Hydrogen Bonding-Assisted and Nonheme Manganese-Catalyzed Remote Hydroxylation of C–H Bopnds in Nitrogen-Containing Molecules. J. Am. Chem. Soc. 2023, 145, 27626–27638. 10.1021/jacs.3c09508. 36811463

[ref18] Zhu W.; Kumar A.; Xiong J.; Abernathy M. J.; Li X.-X.; Seo M. S.; Lee Y.-M.; Sarangi R.; Guo Y.; Nam W. Seeing the cis-Dihydroxylating Intermediate: A Mononuclear Nonheme Iron-Peroxo Complex in *cis*-Dihydroxylation Reactions Modeling Rieske Dioxygenases. J. Am. Chem. Soc. 2023, 145, 4389–4393. 10.1021/jacs.2c13551. 36795537 PMC10544271

[ref19] Borrell M.; Costas M. Mechanistically Driven Development of an Iron Catalyst for Selective *Syn*-Dihydroxylation of Alkenes with Aqueous Hydrogen Peroxide. J. Am. Chem. Soc. 2017, 139, 12821–12829. 10.1021/jacs.7b07909. 28767230

[ref20] Kolb H. C.; VanNieuwenhze M. S.; Sharpless K. B. Catalytic Asymmetric Dihydroxylation. Chem. Rev. 1994, 94, 2483–2547. 10.1021/cr00032a009.

[ref21] Koizumi H.; Tanabe M.; Kambe T.; Imaoka T.; Chun W. J.; Yamamoto K. Copper-Bismuth Binary Oxide Clusters: An Efficient Catalyst for Selective Styrene Bisperoxidation. Chem. Lett. 2022, 51, 317–320. 10.1246/cl.210725.

[ref22] Su Y.-L.; De Angelis L.; Tram L.; Yu Y.; Doyle M. P. Catalytic Oxidative Cleavage Reactions of Arylalkenes by *tert*-Butyl Hydroperoxide – A Mechanistic Assessment. J. Org. Chem. 2020, 85, 3728–3741. 10.1021/acs.joc.9b03346. 31990547

[ref23] Terent’ev A. O.; Sharipov M. Y.; Krylov I. B.; Gaidarenko D. V.; Nikishin G. I. Manganese Triacetate as an Efficient Catalyst for Bisperoxidation of Styrenes. Org. Biomol. Chem. 2015, 13, 1439–1445. 10.1039/C4OB01823K. 25469680

[ref24] An G.; Zhou W.; Zhang G.; Sun H.; Han J.; Pan Y. Palladium-Catalyzed Tandem Diperoxidation/C–H Activation Resulting in Diperoxy-oxindole in Air. Org. Lett. 2010, 12, 4482–4485. 10.1021/ol101664y. 20839834

[ref25] Jayaseharan J.; Kishore K. Biomimetric Aerobic Polymerization of Vinyl Monomers. J. Am. Chem. Soc. 1998, 120, 825–826. 10.1021/ja971335s.

[ref26] Samanta P.; Mete S.; Pal S.; Khan M. E. H.; De P. Synthesis, Characterization, Degradation and Applications of Vinyl Polyperoxides. Polym. J. 2024, 56, 283–296. 10.1038/s41428-023-00860-y.

[ref27] Lee Y.; Kim B.; Kim S.; Ng E. W. H.; Ariyasu S.; Shoji O.; Yoon S.; Hirao H.; Cho J. Influence of Solvents on Catalytic C–H Bond Oxidation by a Copper(II)-Alkylperoxo Complex. ACS Catal. 2024, 14, 3524–3532. 10.1021/acscatal.3c05643.

[ref28] Downing A. N.; Coggins M. K.; Poon P. C. Y.; Kovacs J. A. Influence of Thiolate Versus Alkoxide Ligands on the Stability of Crystallographically Characterized Mn(III)-Alkylperoxo Complexes. J. Am. Chem. Soc. 2021, 143, 6104–6113. 10.1021/jacs.0c13001. 33851827 PMC12024730

[ref29] Opalade A. A.; Parham J. D.; Day V. W.; Jackson T. A. Characterization and Chemical Reactivity of Room-Temperature-Stable Mn^III^-Alkylperoxo Complexes. Chem. Sci. 2021, 12, 12564–12575. 10.1039/D1SC01976G. 34703542 PMC8494025

[ref30] Kumar P.; Lindeman S. V.; Fiedler A. T. Cobalt Superoxo and Alkylperoxo Complexes Derived from Reaction of Ring-Cleaving Dioxygenase Models with O_2_. J. Am. Chem. Soc. 2019, 141, 10984–10987. 10.1021/jacs.9b05320. 31251607 PMC6748656

[ref31] Rispens M. T.; Gelling O. J.; de Vries A. M.; Meetsma A.; van Bolhuis F.; Feringa B. L. Catalytic Epoxidation of Unfunctionalized Alkenes by Dinuclear Nickel(II) Complexes. Tetrahedron 1996, 52, 3521–3546. 10.1016/0040-4020(96)00030-0.

[ref32] Chen Y.; Shi H.; Lee C. S.; Yiu S. M.; Man W. L.; Lau T. C. Room Temperature Aerobic Peroxidation of Organic Substrates Catalyzed by Cobalt(III) Alkylperoxo Complexes. J. Am. Chem. Soc. 2021, 143, 14445–14450. 10.1021/jacs.1c07158. 34477359

[ref33] Saussine L.; Brazi E.; Robine A.; Mimoun H.; Fischer J.; Weiss R. Cobalt(III) Alkylperoxo Complexes. Synthesis, X-ray Structure, and Role in the Catalytic Decomposition of Alkyl Hydroperoxides and in the Hydroxylation of Hydrocarbons. J. Am. Chem. Soc. 1985, 107, 3534–3540. 10.1021/ja00298a022.

[ref34] Achord J. M.; Hussey C. L. Determination of Dissolved Oxygen in Nonaqueous Electrochemical Solvents. Anal. Chem. 1980, 52, 601–602. 10.1021/ac50053a061.

[ref35] Maillard B.; Ingold K. U.; Scaiano J. C. Rate Constants for the Reactions of Free Radicals with Oxygen in Solution. J. Am. Chem. Soc. 1983, 105, 5095–5099. 10.1021/ja00353a039.

