# Supplementary material for: Selective Aerobic Peroxidation of Styrene Catalyzed by a Cobalt tert-Butylperoxo Complex
Source: JACS Au. 2025 Feb 28;5(3):1090–5. doi: 10.1021/jacsau.5c00139 (PMC11938028; doi:10.1021/jacsau.5c00139)
Supplement: Supplementary file 2 — au5c00139_si_002.pdf [file au5c00139_si_002.pdf]

Supporting Information

Selective Aerobic Peroxidation of Styrene Catalyzed by a Cobalt *tert*-Butylperoxo Complex

Yunzhou Chen,[a] Huiying Song,[b] Yiming Hao,[a] Matthew Y. Lui,[a] Wing-Leung Wong,[b] William Wai-Yan Lam,[c] Bun Chan,*[d] Huatian Shi,*[e] and Wai-Lun Man*[a]

[a]Department of Chemistry, Hong Kong Baptist University, Waterloo Road, Kowloon Tong, HKSAR, PR China.

[b]Department of Applied Biology and Chemical Technology, The Hong Kong Polytechnic University, Hung Hom, Kowloon, HKSAR, PR China.

[c]Department of Food and Health Sciences, Technological and Higher Education Institute of Hong Kong, Tsing Yi, New Territories, HKSAR, PR China.

[d]Graduate School of Engineering, Nagasaki University, Bunkyo 1-14, Nagasaki 852-8521, Japan.

[e]School of Environment and Civil Engineering, Research Center for Eco-environmental Engineering, Dongguan University of Technology, Guangdong, PR China.

*To whom all correspondence should be addressed. Email: bun.chan@nagasaki-u.ac.jp (B. Chan); shihuatian@dgut.edu.cn (H. Shi); wlman118@hkbu.edu.hk (W. L. Man).

Experimental Section

Materials

The compound $[Co^{III}(qpy)(OO^tBu)](ClO_4)_2$ **[1](ClO4)2** was prepared according to the literature report.[1] Styrene and *para*-substituted styrene (TCI) were purified by passing through short neutral alumina columns before use. d_3-Acetonitrile (99.8% D) was purchased from Cambridge Isotope Laboratories, Inc. ^{18}O-labeled water (98% ^{18}O atom enrichment) was purchased from Medical Isotopes Inc. $^{18}O_2$ gas was generated by the oxidation of 98% ^{18}O-atom $H_2{}^{18}O$ water using ammonium cerium(IV) nitrate, catalyzed by $Ru(bda)(pic)_2$.[2] All solvents were of reagent grade and used as received unless otherwise stated.

Instrumentation

NMR spectroscopy was performed on a Bruker 400 MHz NMR Ascend or Ultrashield Plus spectrometer. UV-vis spectra were taken on Agilent Cary 8454 UV-Visible Spectrophotometer equipped with PCB 1500 water Peltier system. Electrospray Ionization Mass Spectrometry was conducted on AB Sciex API-3200 Qtrap mass spectrometer. Infrared spectra were recorded on Pekin Elmer Paragon 1000 PC. GC-MS were done with Agilent 7890 GC with Dual FID & 5977MSD equipped with HP-5MS capillary columns (30 m × 250 μm × 0.25 μm). CHN elemental analysis was performed with a varioMICRO CHNS instrument.

X-ray crystallography

The single crystal of **2** was sealed in a loop and mounted on a goniometer head. Measurements were done on a Bruker SMART APEX II CCD area detector system with a graphite-monochromated Mo Kα radiation (λ = 0.71073 Å) in the ω-scan mode. Data acquisition and reduction were performed using the Bruker APEX3 software suite. The structures were solved with SHELXT-2015 and refined on SHELXL-2014 or -2017 with the Olex2 program package.[3–5]

Synthesis of [Co^{III}(qpy)(OOCH(OO^{t}Bu)CH_2Ph)($NCCH_3$)](ClO_4)$_2$ 2(ClO_4)$_2$

The mixture containing **1(ClO_4)$_2$** (53 mg, 0.076 mmol) and styrene (0.5 mL, 4.36 mmol) in 2 mL CH_3CN was stirred for 8 h at ambient conditions. The resulting solution was poured into 15 mL diethyl ether with vigorous stirring to afford the green solid, which was filtered and air-dried. **2** was recrystallized from the solution of CH_3CN/^{i}PrOH at −40 °C. Yield: (49 mg, 77%). Single crystals suitable for X-ray analysis were obtained by standing the solution of **2** in CH_3CN/^{i}PrOH (1:1) at −40 °C for a week. IR (KBr, cm^{-1}): ν(Cl–O) = 1090, ν(O–O) = 891, 866. Anal. Calcd. for $C_{34}H_{34}N_5O_{12}Cl_2Co$: C, 48.94; H, 4.11; N, 8.39. Found: C, 49.20; H, 4.13; N, 8.27. ^{1}H NMR (400 MHz, CD_3CN) δ 9.35 – 9.22 (m, 2H), 8.68 – 8.29 (m, 10H), 8.03 (dtd, J = 13.3, 5.7, 3.4 Hz, 2H), 7.36 (ddd, J = 8.7, 6.8, 4.5 Hz, 1H), 7.21 – 7.14 (m, 1H), 7.12 – 7.02 (m, 2H), 6.53 – 6.39 (m, 2H), 4.06 – 3.87 (m, 1H), 3.65 – 3.55 (m, 1H), 3.53 – 3.40 (m, 2H), 1.16 (d, J = 9.3 Hz, 2H), 0.96 (s, 5H). UV/vis (CH_3CN): λ_{max}[nm] (ε [$mol^{-1}dm^3\ cm^{-1}$]) 207 (77000), 293 (24760), 612 (250).

NMR studies

Under air: The reaction progress between **1** and styrene carried out under air was monitored by NMR at different time intervals as follows: A solution containing **1** (0.50 μmol) and nitromethane (0.50 μmol, as internal standard) in 0.5 mL CD_3CN in an NMR tube was prepared and the NMR spectrum was recorded (time = 0). Then, styrene (0.10 mmol) was added to the solution, and the NMR spectra at various time intervals were recorded. *Control experiments show that there is no reaction between **1** and nitromethane for at least 24 h.*

Under Ar: For the reaction done under argon, an NMR tube containing **1** (0.50 μmol) was placed in a Schlenk tube and degassed using the Schlenk technique. A degassed solution of styrene (0.10 mmol) and CH_3NO_2 (0.50 μmol) in CD_3CN (0.5 mL) was transferred to the NMR tube under argon. The NMR tube was sealed and measurements were done at various time intervals.

Determination of activation parameters

According to the Eyring equation, activation parameters *($\Delta H^{\ddagger}$ and $\Delta S^{\ddagger}$)* were obtained from the plot of ln(*k*/T) versus 1/T $\ln\frac{k}{\mathrm{T}} = \frac{-\Delta H^{\ddagger}}{R} \times \frac{1}{\mathrm{T}} + \left(\ln\frac{k_{\mathrm{B}}}{h} + \frac{\Delta S^{\ddagger}}{R}\right)$. *The* enthalpy *($\Delta H^{\ddagger}$)* of activation was calculated from the slope which is equal to $\frac{-\Delta H^{\neq}}{R}$ (where R is the Gas constant) while the entropy ($\Delta S^{\neq}$) of activation was calculated from the y-intercept of the Eyring plot which is equal to $\ln\frac{\mathrm{k_B}}{h} + \frac{\Delta \mathrm{S}^{\ddagger}}{R}$ (where k_{B} is the Boltzmann's constant and *h* is the Planck constant). Gibbs energy of activation ($\Delta G^{\ddagger}$) was calculated according to the equation:

$\Delta G^{\ddagger} = \Delta H^{\ddagger} - \mathrm{T}\Delta S^{\ddagger}$.

Computational Studies

Calculations on cobalt complexes utilized density functional theory (DFT) as implemented in Orca 5.0.4 program package.[6-7] Geometry optimization was performed with unrestricted B3LYP functional without imposing any symmetry constraints.[8-11] The resolution of the identity chain of spheres ("RIJCOSX") approximation was used in the calculation.[12-13] A triple-ζ Ahlrichs basis set def2-TZVP and def2/J auxiliary basis were used on all atoms.[14-15] Becke-Johnson damping scheme (D3BJ) was adapted to include dispersion in all the calculations.[16-17] Solvent CH_3CN was applied to all calculations with the conductor-like polarizable continuum model. Tight optimization and tight self-consistent field convergence with Orca default grid (defgrid2) were employed. Frequency calculation was performed on the equilibrium structures and transition states at the same level of theory to ensure the local minimum of the geometry (without any imaginary frequency) or a genuine transition state (with only an imaginary frequency corresponding to TS). Gibbs free energy at 298.15 K is used for energy comparison. Singlet, triplet, broken-symmetry (3:1) triplet and quintet states were considered in the computational analysis; broken-symmetry (1:1) singlet state was not considered as we could not converge the electronic configurations of **I**, **II** and **VI** to broken-symmetry (1:1) singlet state. The spin densities were plotted with a 0.03

isosurface value and Loewdin population analysis was used to identify the spin density of the atoms. The equilibrium structures of carbon-centered radical rebound cobalt complexes were also studied by the same methods, and the results show equilibrium structures of triplet and broken-symmetry (3:1) triplet states converge to the same structure with identical electronic structure. The energies of the structures are 6.1(singlet), 9.7(triplet), and 9.1(quintet) *k*cal/mol, respectively, with reference to the sum of the energy of **1** and styrene.

Table S1 Summary of crystal data, details of data collection solution, and refinement parameters for complexes **2(ClO$_4$)$_2$**.

	**2(ClO$_4$)$_2$·CH$_3$CN**
Formula	$C_{36}H_{37}Cl_2CoN_6O_{12}$
*M*r	875.54
Crystal system	Monoclinic
Space group	$P2_1/n$
a/Å	14.345 (1)
b/Å	15.265 (1)
c/Å	18.303 (1)
β/(°)	100.593 (1)
V/ Å^3	3939.6 (2)
Z	4
D_c/Mg m^{-3}	1.476
F(000)	1808
Collected reflections	8010
R(int)	0.040
Final *R* indices,	R_1(obs) = 0.068
$I > 2\ \theta(I)\ R$	*wR*(all) = 0.207
GOF	1.05
No. of parameters	648

Table S2 Selected bond lengths (Å) and angles (°) of **2(ClO$_4$)$_2$·CH$_3$CN**.

Bond lengths (Å)		Angles (°)	
Co1−O1	1.862(2)	O2A−O1−Co1	119.3(5)
Co1−N1	1.990(3)	O2−O1−Co1	114.1(2)
Co1−N2	1.866(3)	N1−Co1−N4	114.14(12)
Co1−N3	1.864(3)	N2−Co1−N1	81.86(12)
Co1−N4	1.992(3)	N2−Co1−N4	163.99(13)
Co1−N5	1.942(3)	N2−Co1−N5	91.64(13)
O1−O2A	1.399(12)	N3−Co1−N1	164.14(12)
O1−O2	1.425(5)	N3−Co1−N2	82.29(13)
O2−C21	1.341(8)	N3−Co1−N4	81.71(12)
O3−C22	1.384(10)	N3−Co1−N5	92.45(12)
O3−O4	1.402(12)	N5−Co1−N1	88.60(12)
O4−C29	1.496(16)	N5−Co1−N4	88.68(12)
O3A−O4A	1.394(17)		

Table S3. Second-order rate constants for the reaction of **1** and *para*-substituted styrene.[a]

Entry	Substitute	T (°C)	k_2 (10^{-3} M^{-1} s^{-1})[b]
1	H	10.0	0.45 ± 0.06
2	H	20.0	2.40 ± 0.06
2	H	25.0	4.64 ± 0.10
3	H	30.0	9.28 ± 0.30
4	H	35.0	14.3 ± 0.60
5	H	40.0	30.0 ± 0.30
6	OMe	25.0	7.71 ± 0.22
7	Me	25.0	6.10 ± 0.17
8	F	25.0	3.73 ± 0.16
9	CF_3	25.0	1.75 ± 0.04

[a] Reaction conditions: [**1**] = 0.2 mM, [styrene] = 0.1–1.0 M, T = 10 – 40 °C, solvent = CH_3CN, in air; [b] mean values of triplicate measurements.

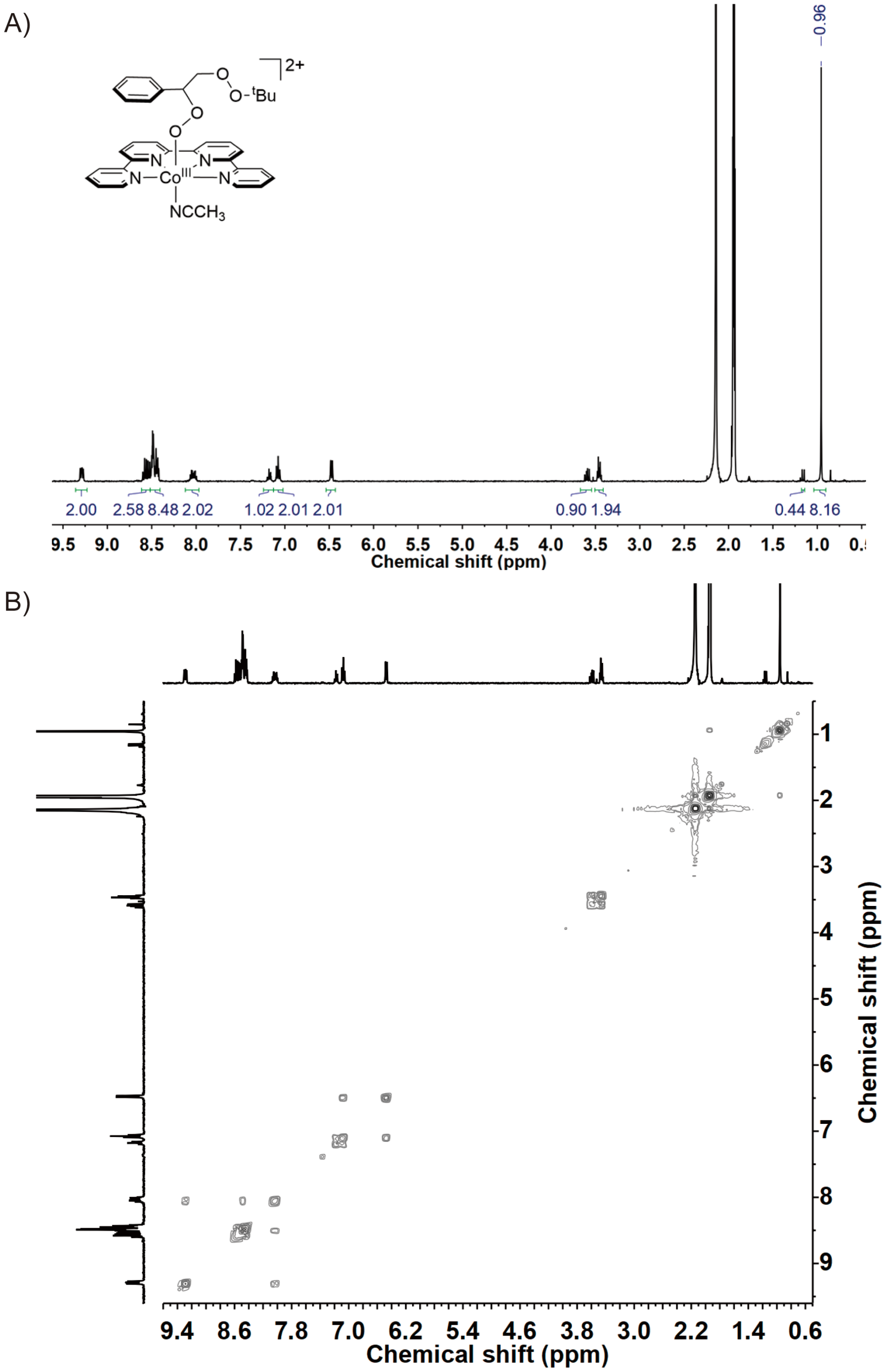

Figure S1 NMR spectra of **2** in CD_3CN: (A) ^{1}H NMR, and (B) ^{1}H-^{1}H COSY.

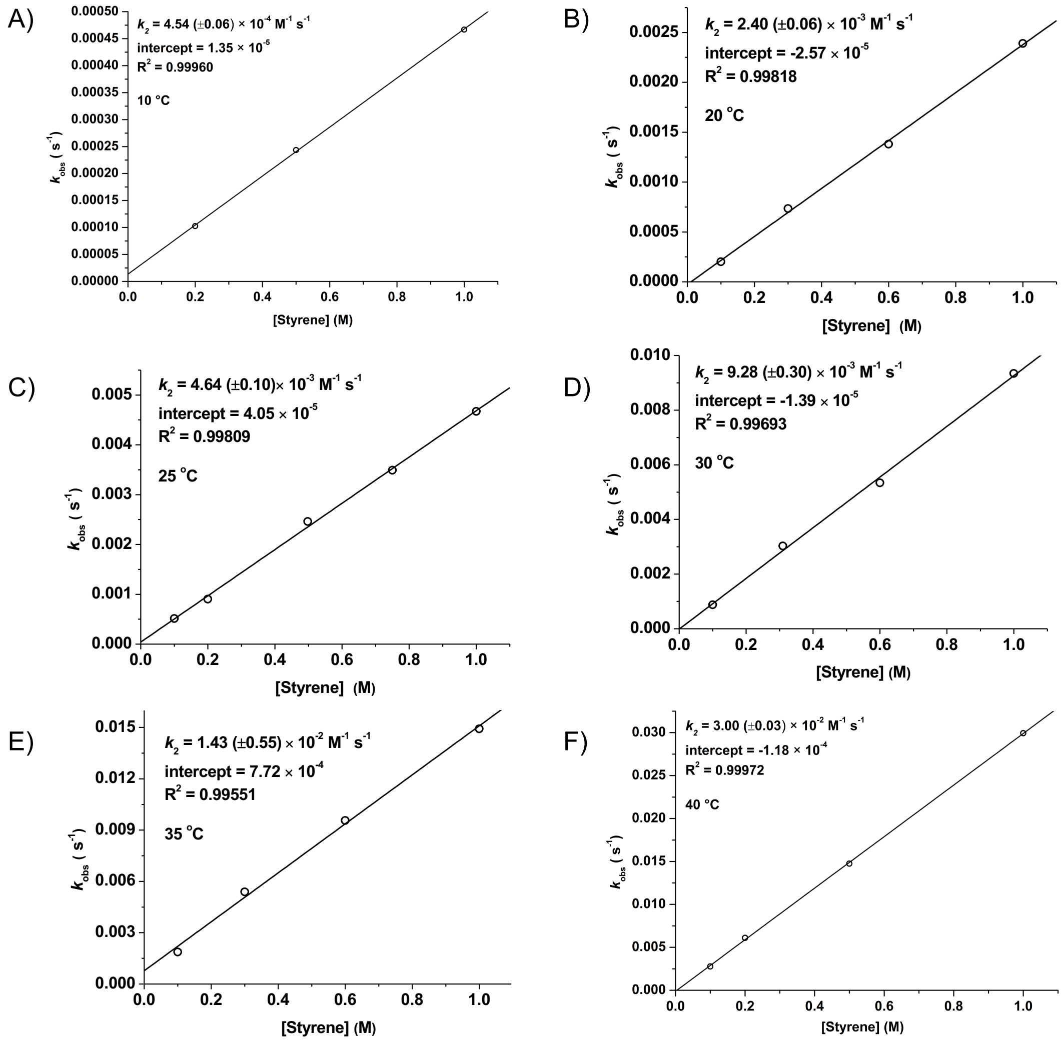

Figure S2 The second-order plots for the reaction of **1** (0.2 mM) and styrene at different temperatures. (A) 10 ºC; (B) 20 ºC; (C) 25 ºC; (D) 30 ºC; (E) 35 ºC; (F) 40 ºC.

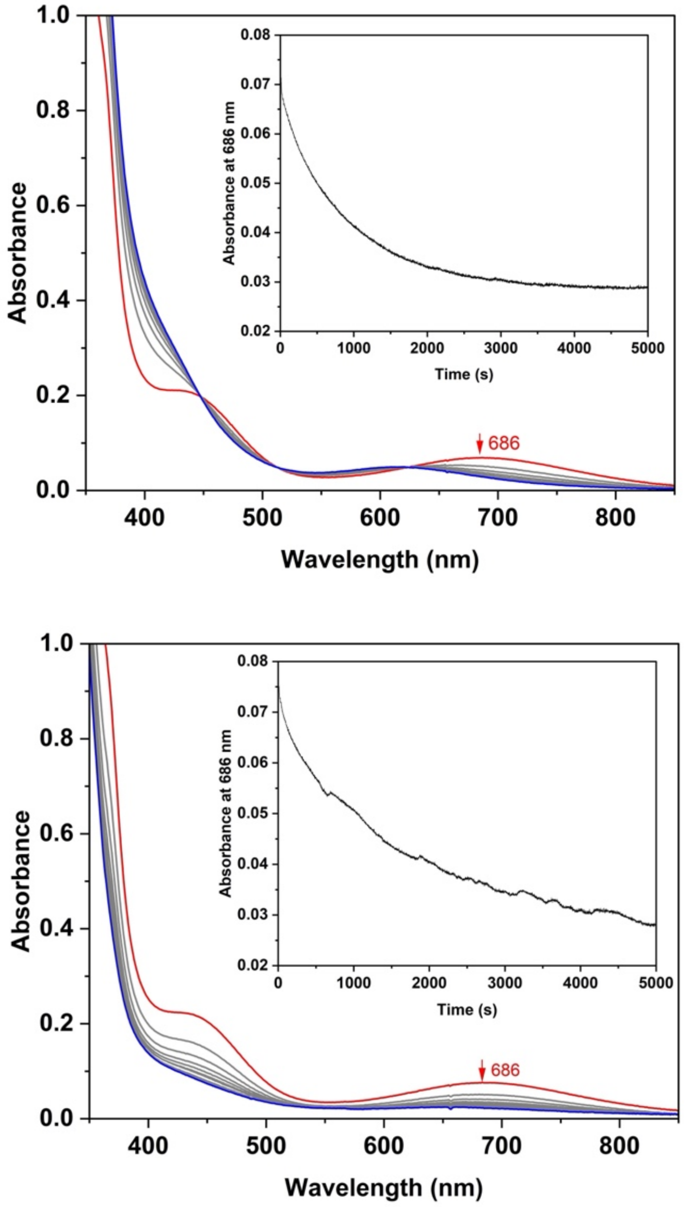

Figure S3. The spectral changes for the reaction of 0.2 mM **1** with 0.2 M styrene in CH_3CN at 25 ^{o}C under O_2 (top) and Ar atmosphere (bottom). Insets show the decay of **1** at 686 nm against time. The k_{obs} values are 1.23×10^{-3} s^{-1} (under O_2) and 0.84×10^{-3} s^{-1} (under Ar), respectively.

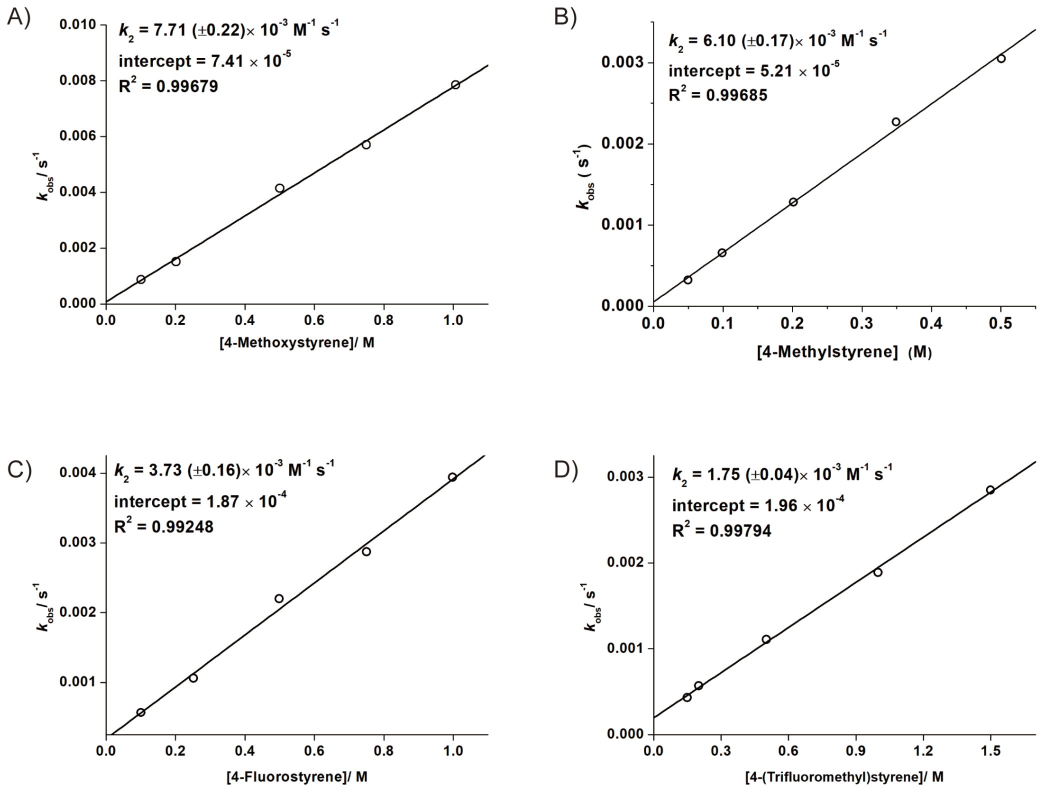

Figure S4 The second-order plots for the reaction of **1** and *para*-substituted styrenes in CH_3CN under air. (A) 4-methyoxystyrene; (B) 4-methylstyrene; (C) 4-fluorostyrene; (D) 4-trifluoromethylstyrene.

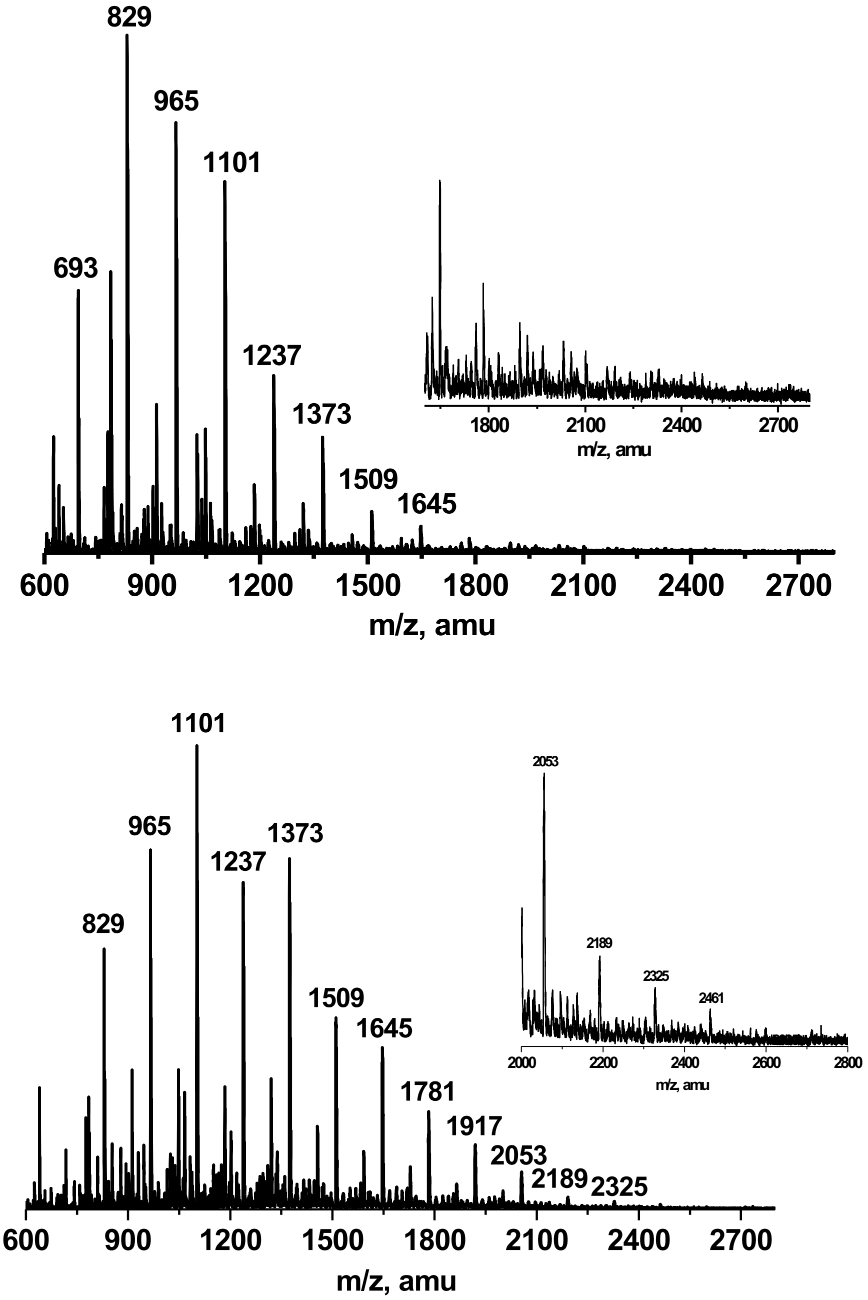

Figure S5 The ESI mass spectra for the solution containing 0.2 mM **1** and 1 M styrene in CH_3CN under O_2 atmosphere. Top: 0.5 h; Bottom 3 h.

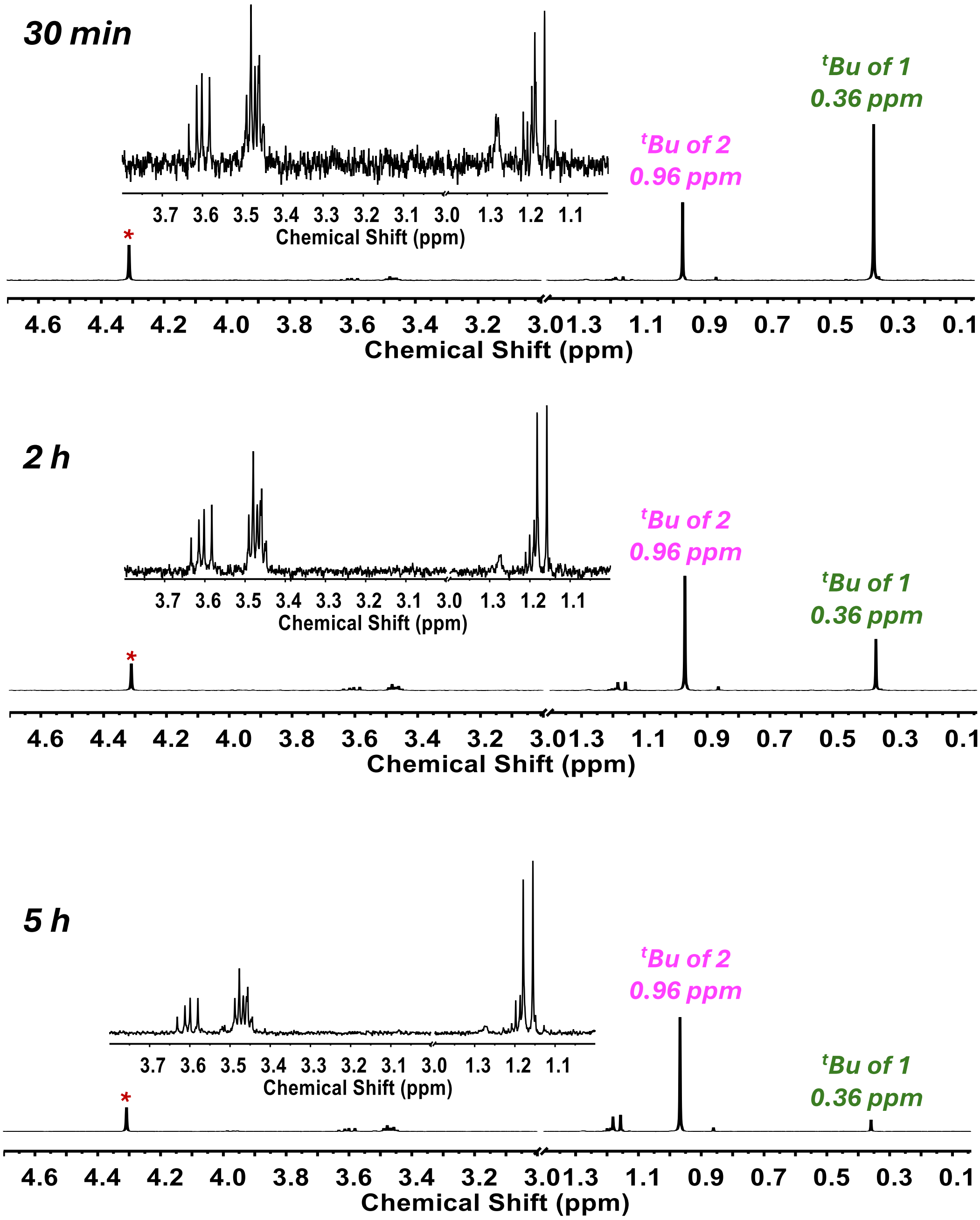

Figure S6 Time-dependent product analysis by ^{1}H NMR in CD_3CN for the reaction of 1 mM **1** and 200 mM styrene. The singlet at 4.3 ppm corresponds to CH_3NO_2, added as the internal standard.

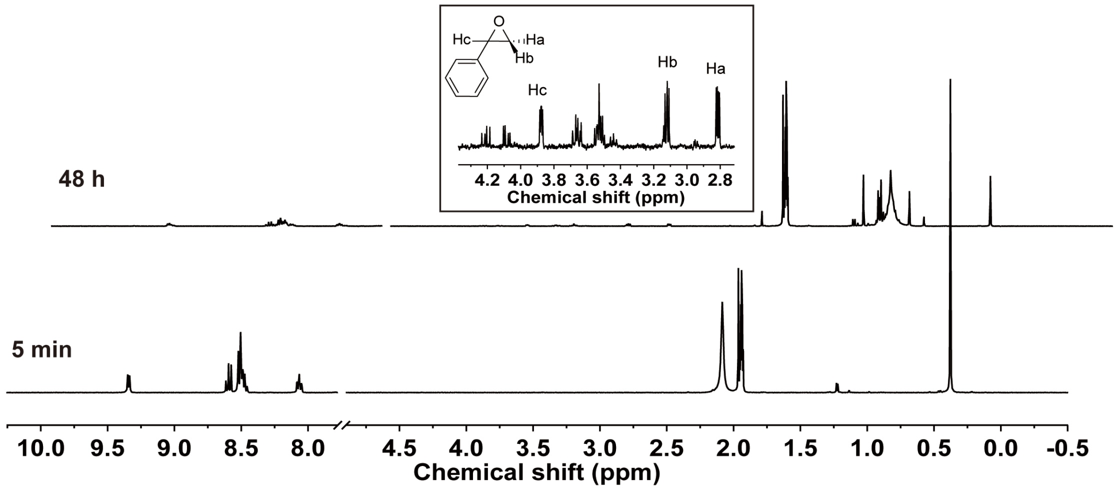

Figure S7 NMR spectra of the reaction between **1** and styrene in CD_3CN under Ar. [The protons of styrene were omitted for clarity]

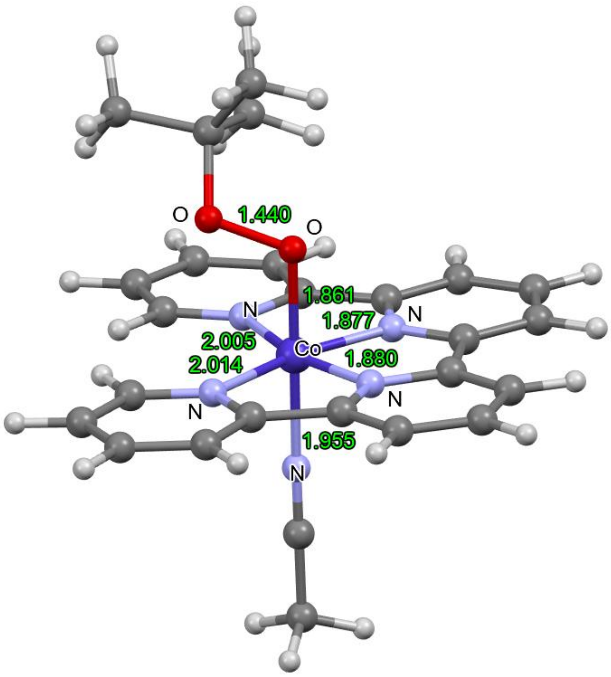

Figure S8 The molecular structure of **1** from DFT calculation at singlet state.

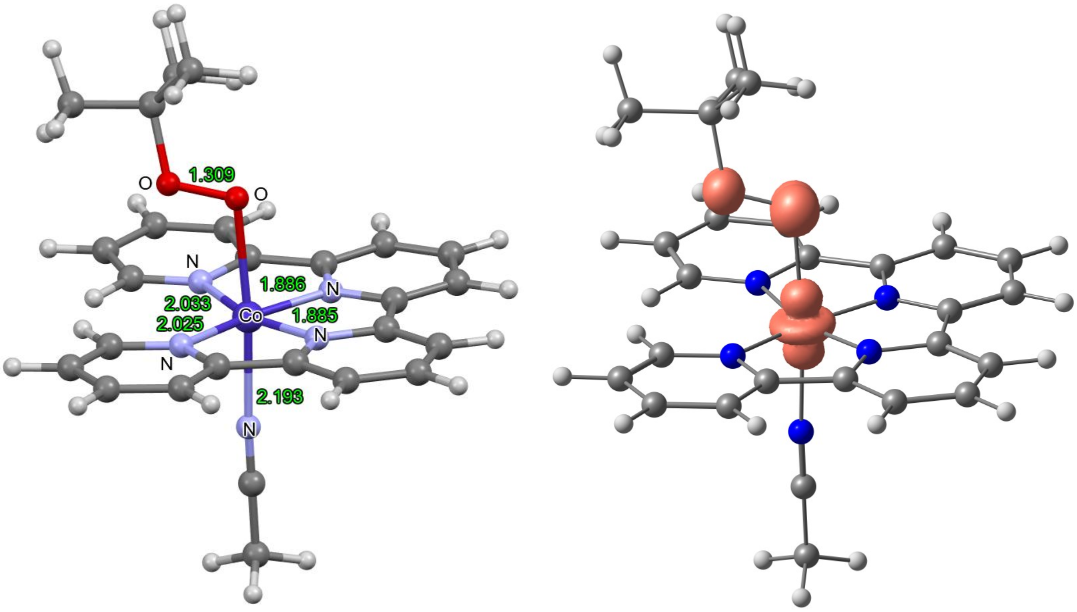

Figure S9 The molecular structure (left) and spin density (right) of **1** from DFT calculation at triplet state.

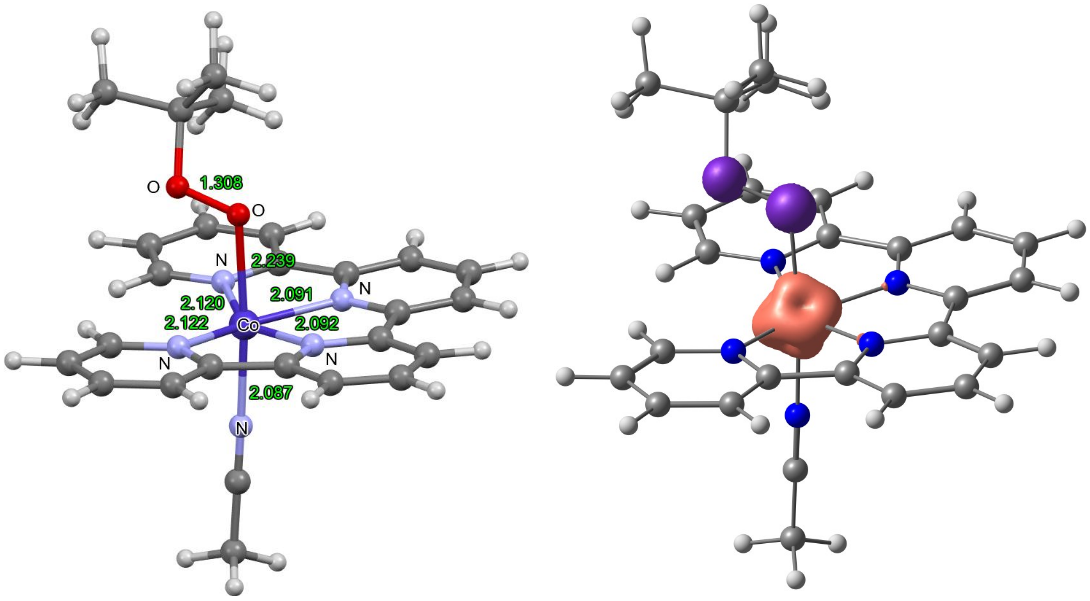

Figure S10 The molecular structure (left) and spin density (right) of **1** from DFT calculation at broken symmetry BS(3,1) triplet state.

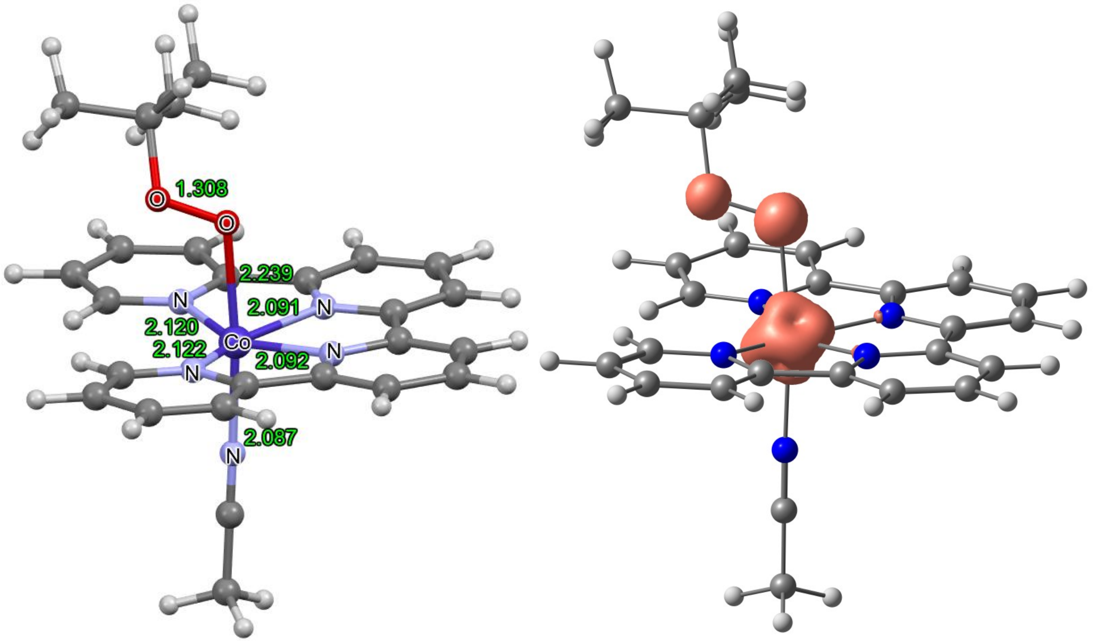

Figure S11 The molecular structure (left) and spin density (right) of **1** from DFT calculation at quintet state.

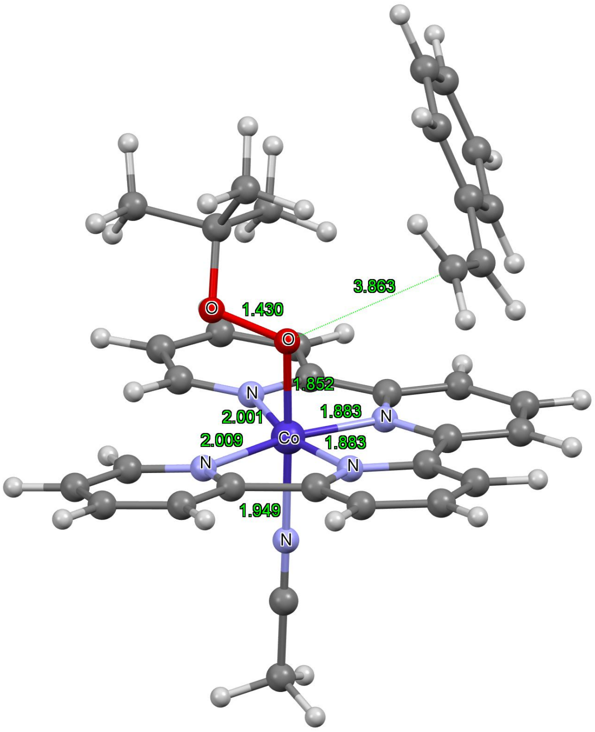

Figure S12 The molecular structure of reaction complex (**II**) between **1** and styrene from DFT calculation at singlet state.

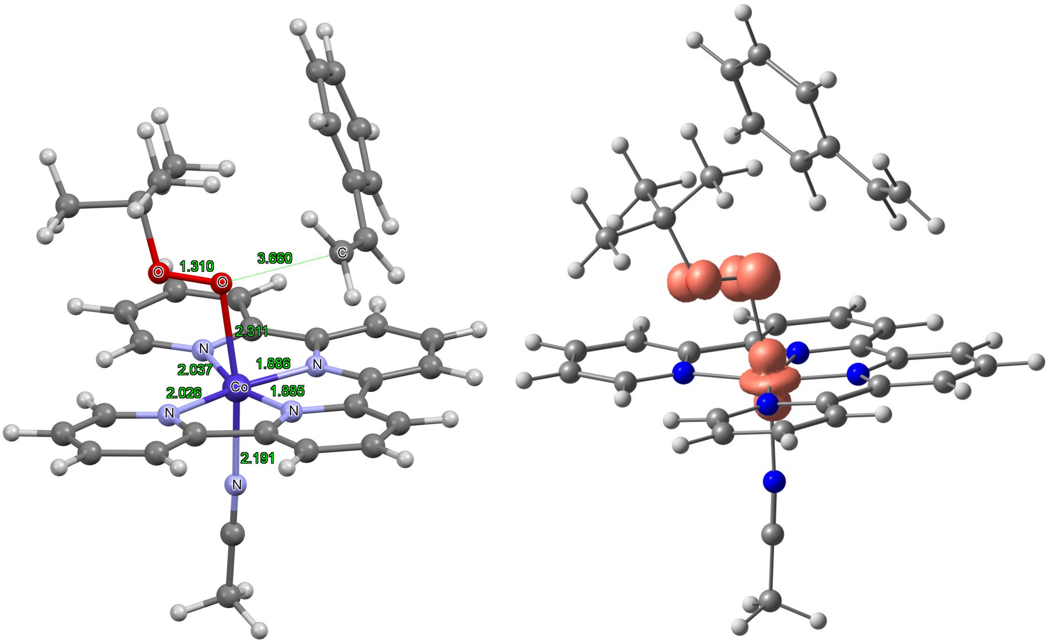

Figure S13 The molecular structure (left) and spin density (right) of reaction complex (**II**) between **1** and styrene from DFT calculation at triplet state.

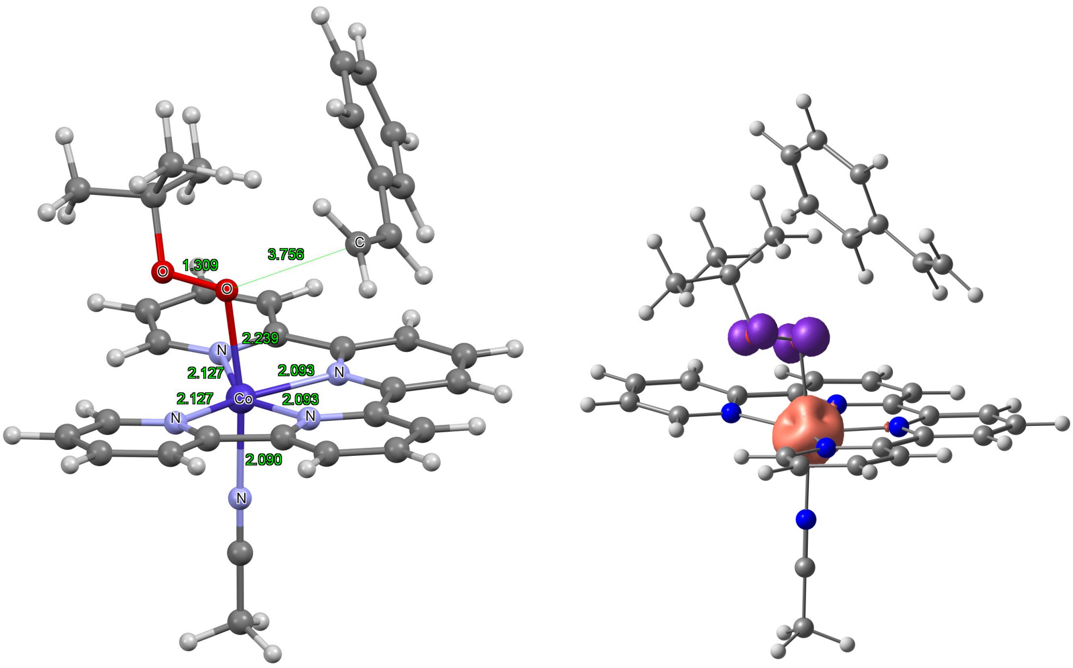

Figure S14 The molecular structure (left) and spin density (right) of reaction complex (**II**) between **1** and styrene from DFT calculation at broken symmetry BS(3,1) triplet state.

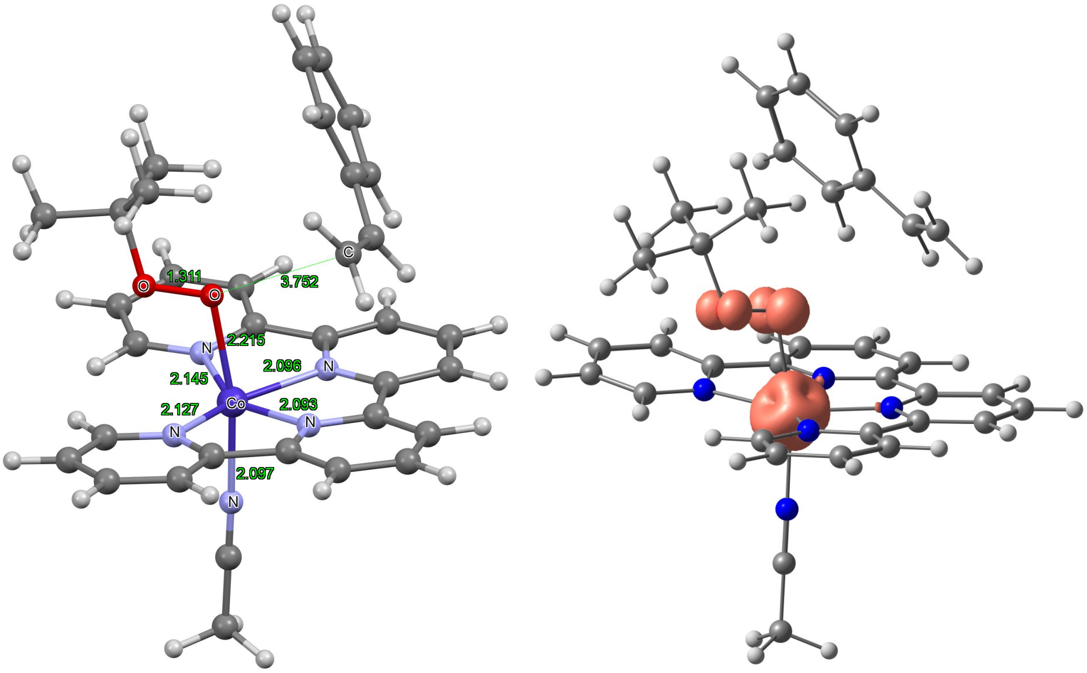

Figure S15 The molecular structure (left) and spin density (right) of the reaction complex (**II**) between **1** and styrene from DFT calculation at quintet state.

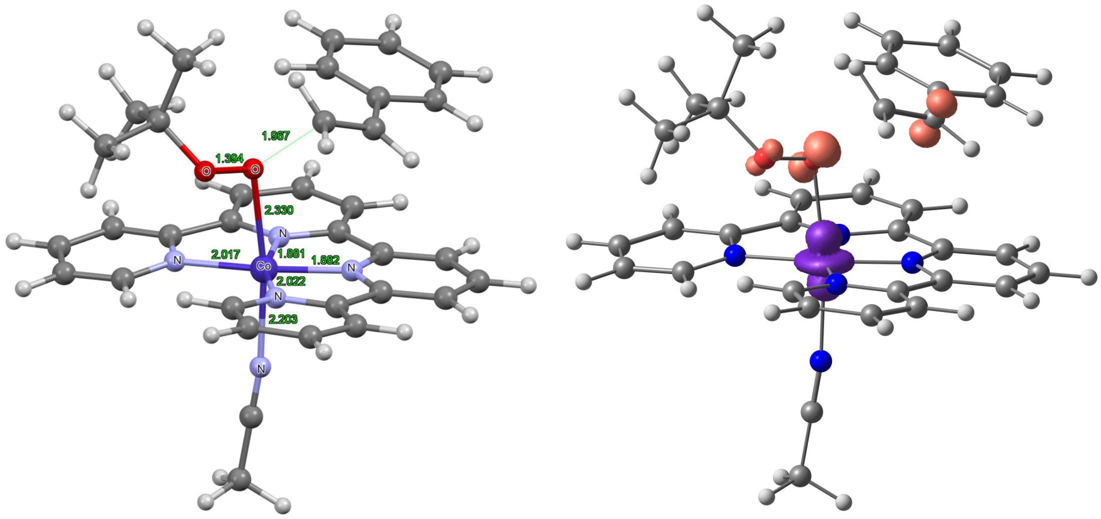

Figure S16 The molecular structure (left) and spin density (right) of the transition state (**III**) between **1** and styrene from DFT calculation at singlet state.

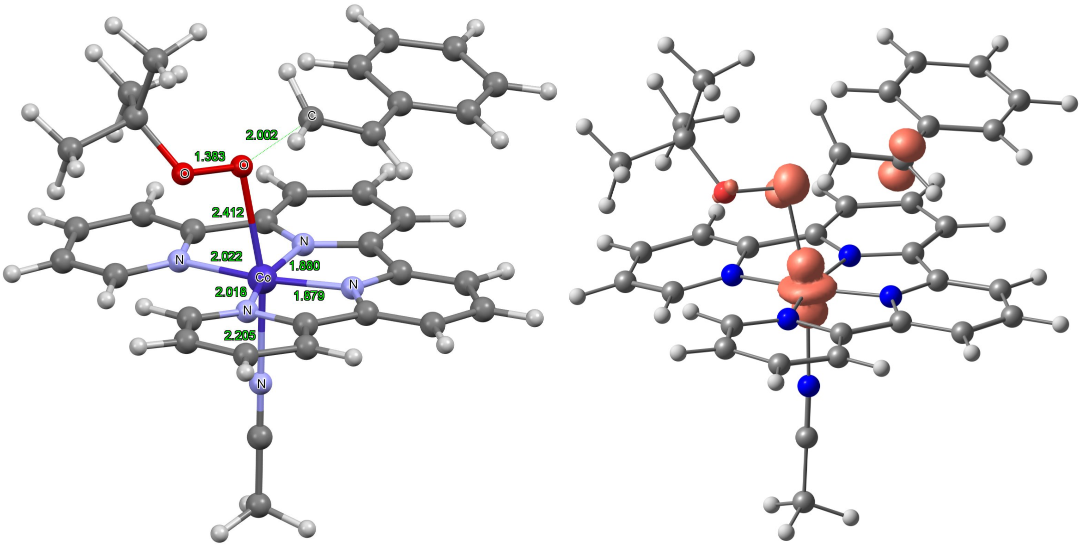

Figure S17 The molecular structure (left) and spin density (right) of the transition state (**III**) between **1** and styrene from DFT calculation at triplet state.

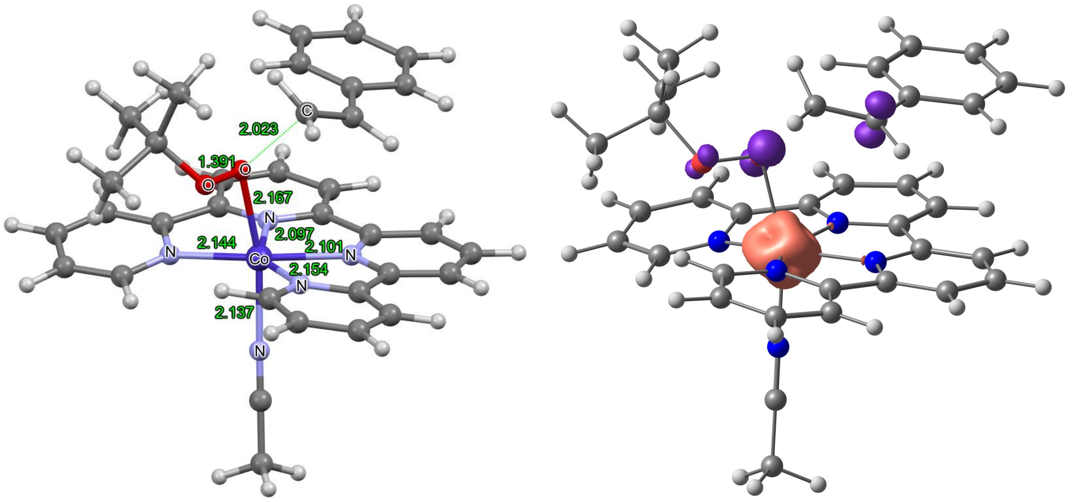

Figure S18 The molecular structure(left) and spin density(right) of the transition state (**III**) between **1** and styrene from DFT calculation at broken symmetry BS(3,1) triplet state.

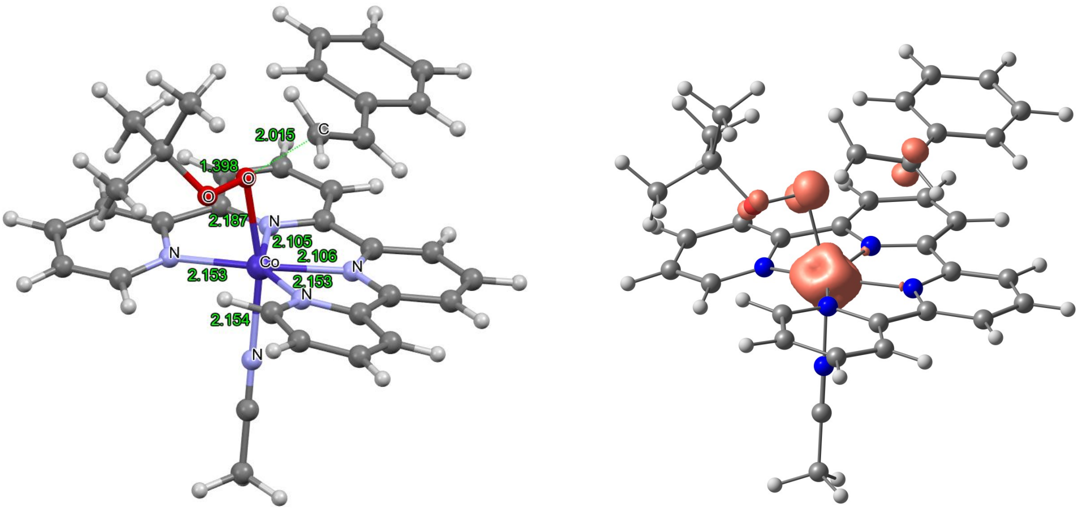

Figure S19 The molecular structure (left) and spin density (right) of the transition state (**III**) between **1** and styrene from DFT calculation at the quintet state.

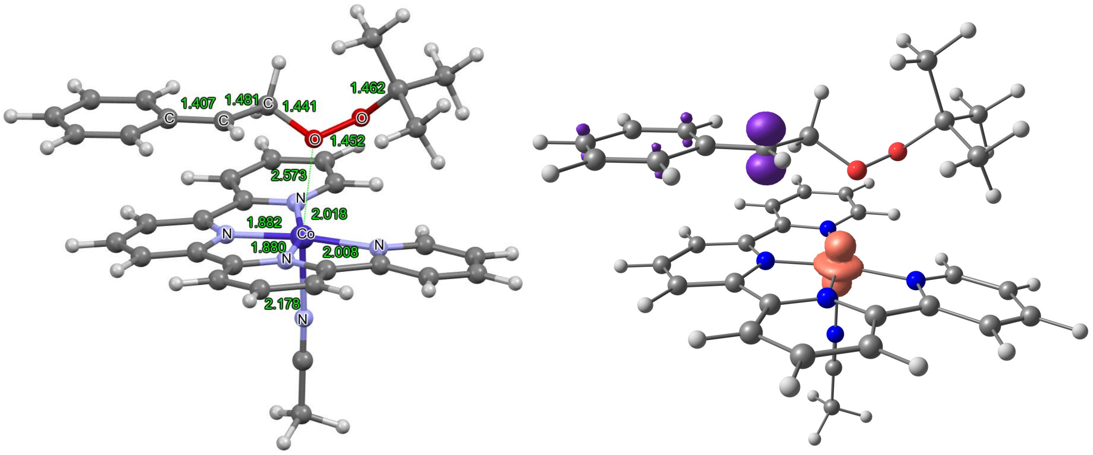

Figure S20 The molecular structure (left) and spin density (right) of intermediate (**IV**) between **1** and styrene from DFT calculation at singlet state.

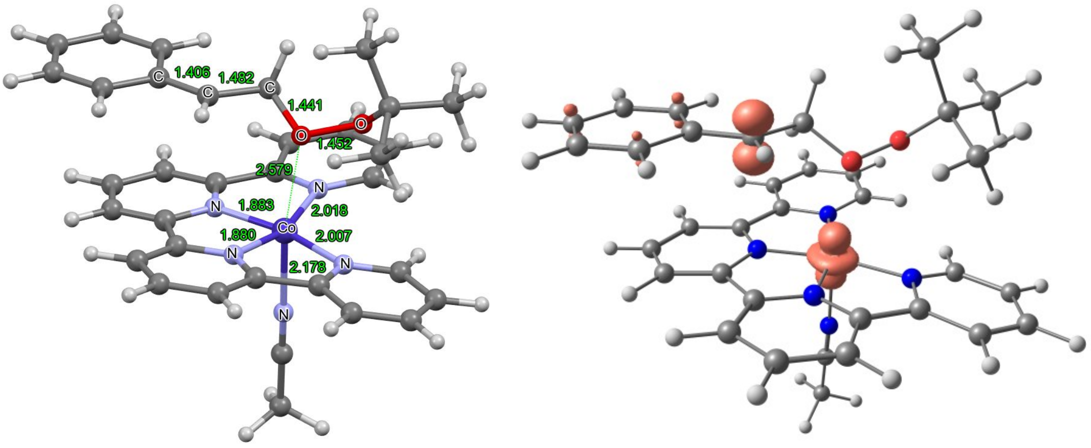

Figure S21 The molecular structure (left) and spin density (right) of intermediate (**IV**) between **1** and styrene from DFT calculation at triplet state.

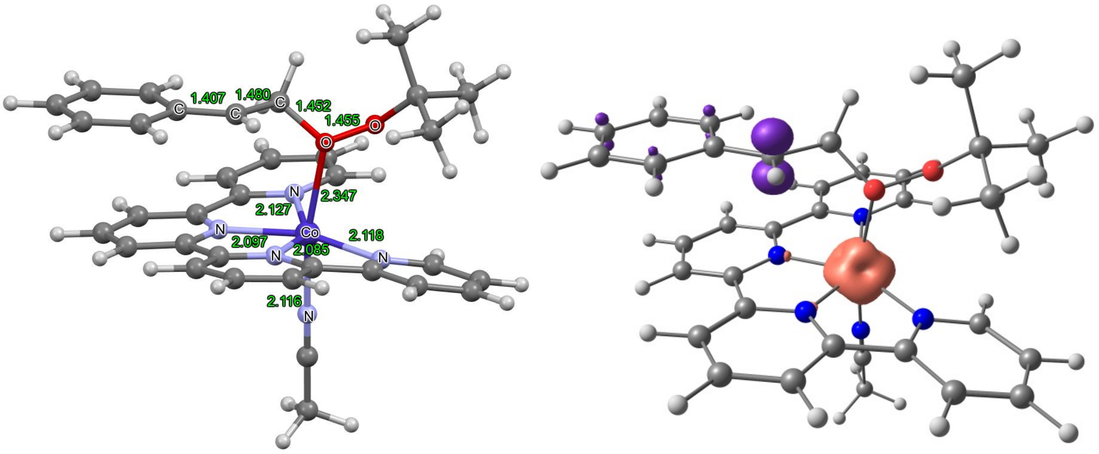

Figure S22 The molecular structure (left) and spin density (right) of intermediate (**IV**) between **1** and styrene from DFT calculation at broken symmetry BS(3,1) triplet state.

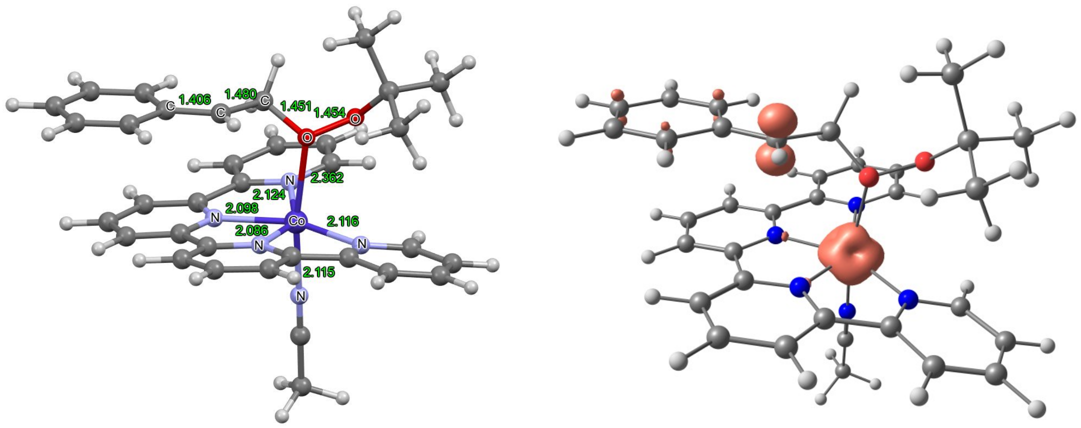

Figure S23 The molecular structure (left) and spin density (right) of intermediate (**IV**) between **1** and styrene from DFT calculation at quintet state.

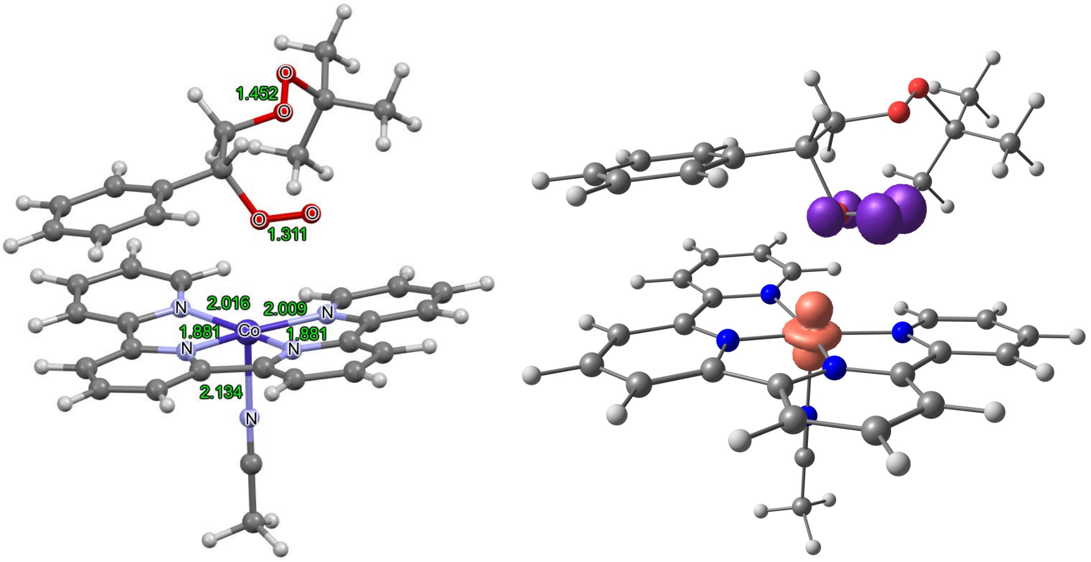

Figure S24 The molecular structure (left) and spin density (right) of O_2-adduct (**V**) of **IV** from DFT calculation at singlet state.

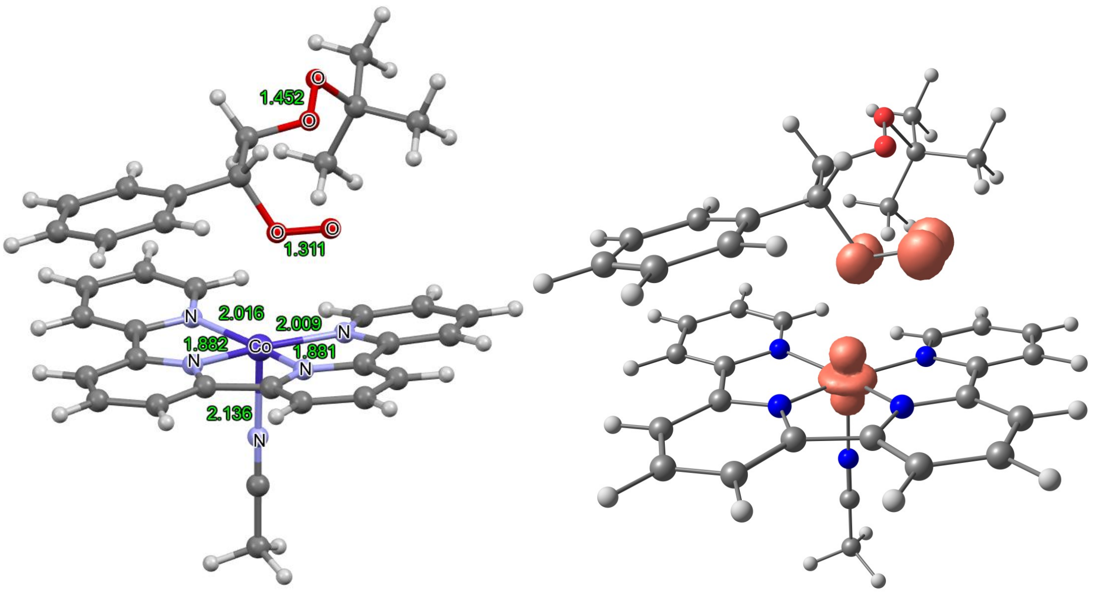

Figure S25 The molecular structure (left) and spin density (right) of O_2-adduct (**V**) of **IV** from DFT calculation at triplet state.

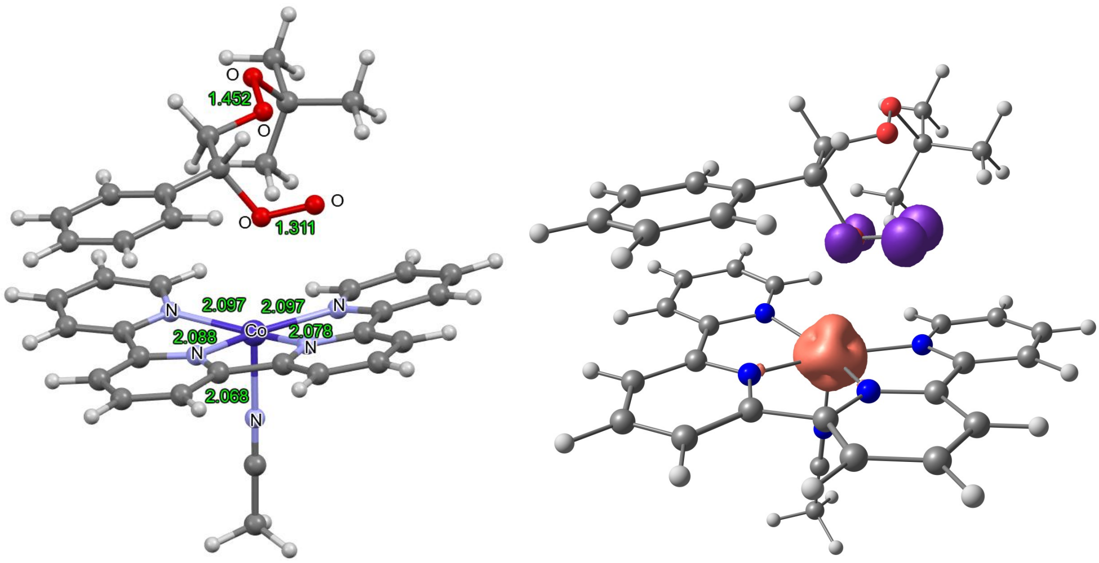

Figure S26 The molecular structure (left) and spin density (right) of O_2-adduct (**V**) of **IV** from DFT calculation at broken symmetry BS(3,1) triplet state.

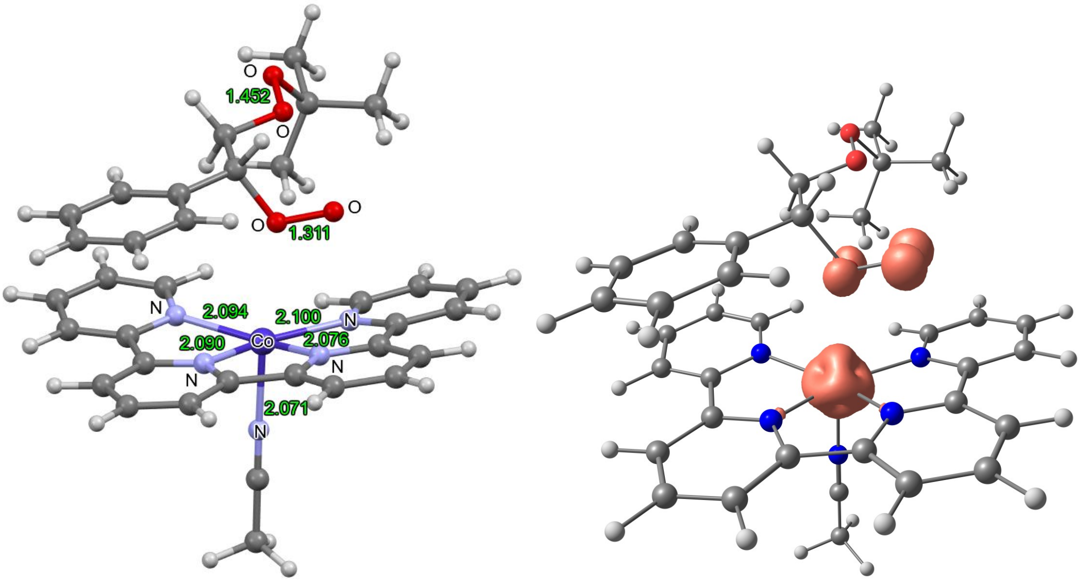

Figure S27 The molecular structure (left) and spin density (right) of O_2-adduct (**V**) of **IV** from DFT calculation at quintet state.

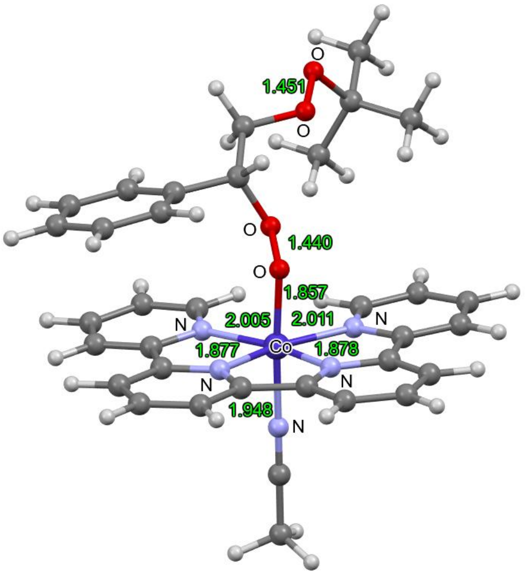

Figure S28 The molecular structure of the product (**VI**) from DFT calculation at singlet state.

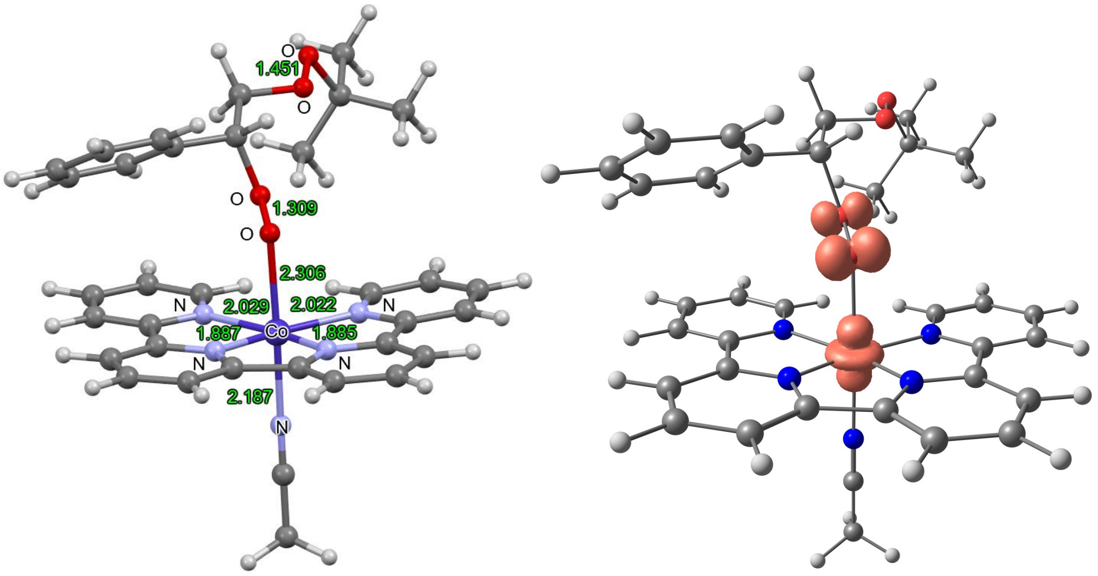

Figure S29 The molecular structure (left) and spin density (right) of the product (**VI**) from DFT calculation at triplet state.

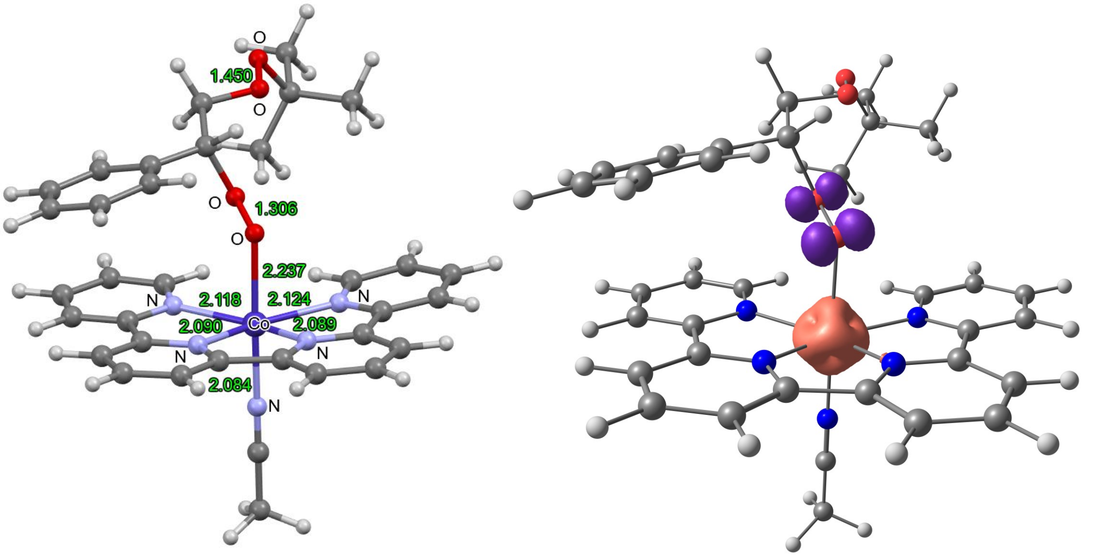

Figure S30 The molecular structure (left) and spin density (right) of the product (**VI**) from DFT calculation at broken symmetry BS(3,1) triplet state.

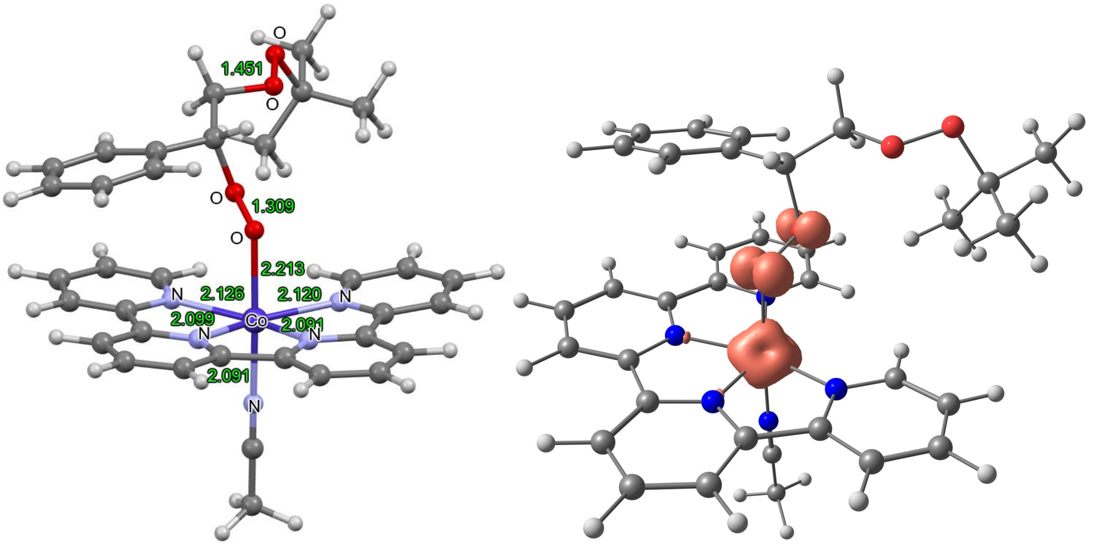

Figure S31 The molecular structure (left) and spin density (right) of the product (**VI**) from DFT calculation at quintet state.

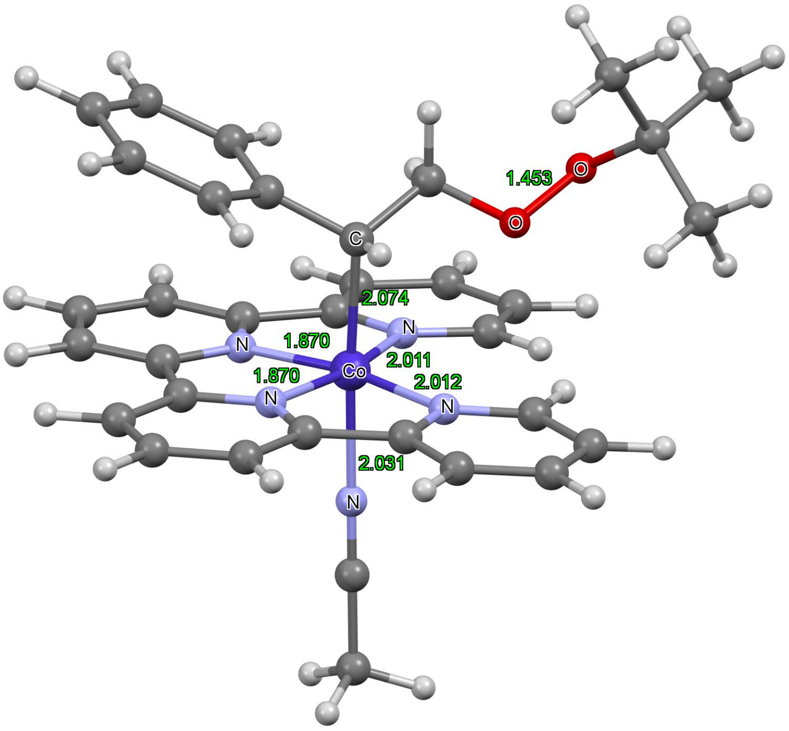

Figure S32 The molecular structure of carbon centered radical rebound cobalt product from DFT calculation at singlet state.

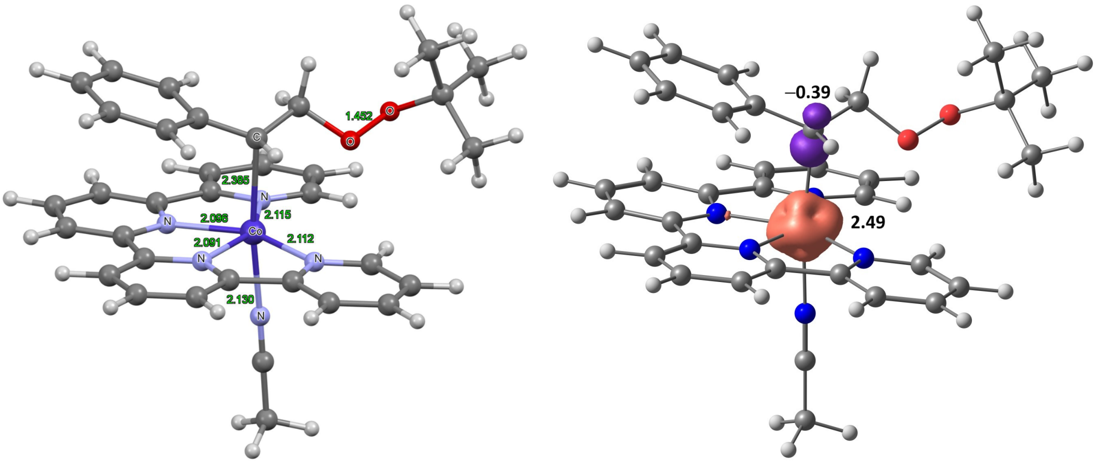

Figure S33 The molecular structure(left) and spin density(right) of carbon centered radical rebound cobalt product from DFT calculation at triplet state.

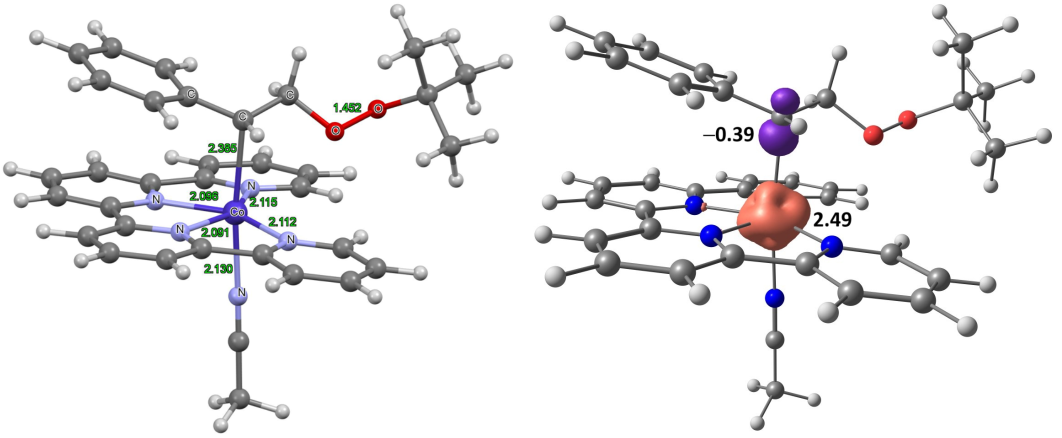

Figure S34 The molecular structure(left) and spin density(right) of carbon centered radical rebound cobalt product from DFT calculation at broken-symmetry (3:1) triplet state.

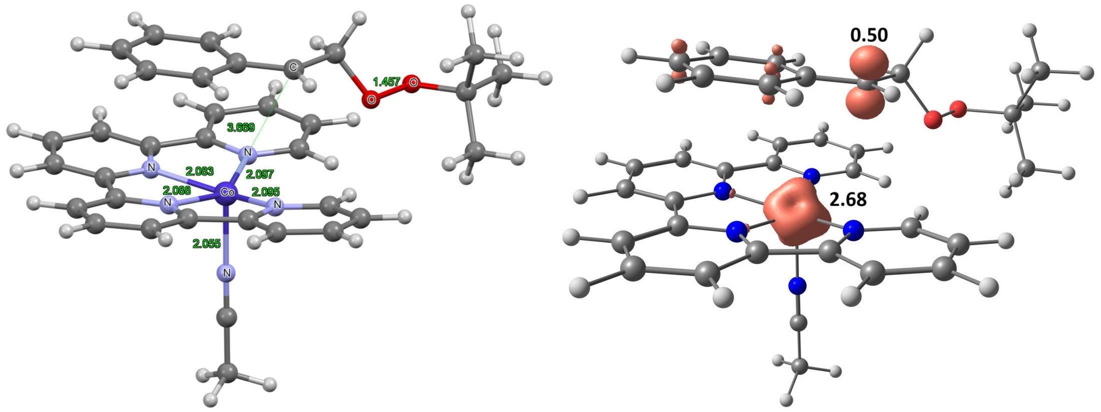

Figure S35 The molecular structure(left) and spin density(right) of carbon centered radical rebound cobalt product from DFT calculation at quintet state.

References

(1) Chen, Y.; Shi, H.; Lee, C. S.; Yiu, S. M.; Man, W. L.; Lau, T. C. Room Temperature Aerobic Peroxidation of Organic Substrates Catalyzed by Cobalt(III) Alkylperoxo Complexes. *J. Am. Chem. Soc.* **2021**, *143*, 14445–14450.

(2) Duan, L.; Fischer, A.; Xu, Y.; Sun, L. *J. Am. Chem. Soc.* **2009**, 131, 10397–10399.

(3) Dolomanov, O. V.; Bourhis, L. J.; Gildea, R. J.; Howard, J. A. K. Puschmann, H. *J. Appl. Cryst*. **2009**, *42*, 339–341.

(4) Sheldrick, G. M. *Acta Cryst*. C. **2015**, *64*, 112–122.

(5) Krause, L.; Herbst-Irmer, R.; Sheldrick, G. M.; Stalke, D. *J. Appl. Cryst*. **2015**, *48*, 3–10

(6) Neese, F., The ORCA program system. *WIREs. Comput. Mol. Sci.* **2012**, 2 (1), 73-78.

(7) Neese, F., Software update: The ORCA program system—Version 5.0. *WIREs. Comput. Mol. Sci.* **2022**, 12 (5), e1606.

(8) Becke, A. D., Density-Functional Thermochemistry .3. The Role of Exact Exchange. *J. Chem. Phys.* **1993**, 98 (7), 5648-5652.

(9) Lee, C.; Yang, W.; Parr, R. G., Development of the Colle-Salvetti correlation-energy formula into a functional of the electron density. *Phys. Rev. B* **1988**, 37 (2), 785-789.

(10) Stephens, P. J.; Devlin, F. J.; Chabalowski, C. F.; Frisch, M. J., Ab Initio Calculation of Vibrational Absorption and Circular Dichroism Spectra Using Density Functional Force Fields. *J. Phys. Chem.* **1994**, 98 (45), 11623-11627.

(11) Vosko, S. H.; Wilk, L.; Nusair, M., Accurate spin-dependent electron liquid correlation energies for local spin density calculations: a critical analysis. *Can. J. Phys.* **1980**, 58 (8), 1200-1211.

(12) Neese, F.; Wennmohs, F.; Hansen, A.; Becker, U., Efficient, approximate and parallel Hartree-Fock and hybrid DFT calculations. A 'chain-of-spheres' algorithm for the Hartree-Fock exchange. *Chem. Phys.* **2009**, 356 (1-3), 98-109.

(13) Izsak, R.; Neese, F., An overlap fitted chain of spheres exchange method. *J. Chem. Phys.* **2011**, 135 (14).

(14) Weigend, F.; Ahlrichs, R., Balanced basis sets of split valence, triple zeta valence and quadruple zeta valence quality for H to Rn: Design and assessment of accuracy. *Phys. Chem. Chem. Phys.* **2005**, 7 (18), 3297-3305.

(15) Weigend, F., Accurate Coulomb-fitting basis sets for H to Rn. *Phys. Chem. Chem. Phys.* **2006**, 8 (9), 1057-1065.

(16) Grimme, S.; Ehrlich, S.; Goerigk, L., Effect of the Damping Function in Dispersion Corrected Density Functional Theory. *J. Comput. Chem.* **2011**, 32 (7), 1456-1465.

(17) Grimme, S.; Antony, J.; Ehrlich, S.; Krieg, H., A consistent and accurate ab initio parametrization of density functional dispersion correction (DFT-D) for the 94 elements H-Pu. *J. Chem. Phys.* **2010**, 132 (15).

xyz coordinates of calculated structures

Coordinates of I at singlet state

Co	6.49003333916184	2.65522917661633	2.60388029210629
O	5.66261679712179	1.08039074365352	3.15029255505361
O	4.26271116422131	1.35910923777074	3.34253989515713
N	7.53227216757495	1.58606186252935	1.46099470835118
N	5.28325229764391	2.69125688209565	0.99173734530063
N	5.80836407021507	3.69331501362113	4.17755667770055
N	7.35825347827024	4.28327102236126	1.95645908292199
N	7.90999590819828	2.32042446144363	3.78542288515489
C	5.78461624903819	1.95744112416915	-0.03715286180293
C	6.66846116474649	3.66108472060289	5.22908031799529
C	9.98120396766430	1.26405509512339	4.17410287189114
H	10.79615985618022	0.64060309337394	3.83583145513142
C	7.07823966287640	1.31743268914733	0.23927891538291
C	8.92494946903729	2.61190336204947	5.88440511424314
H	8.92303836813908	3.03406764266461	6.87814321078634
C	7.83043696234438	0.49887126156075	-0.59901797249839
H	7.48982715482349	0.26177873650752	-1.59584716813220
C	8.68236412065610	1.10034586845791	1.95607156041161
C	9.03111989834240	-0.01209441118688	-0.12232784171192
H	9.62917877742313	-0.64981739879242	-0.75864181402287
C	7.87635308007051	2.85758704269501	5.00294264383408
C	9.97448953484978	1.80836397632930	5.45632357614524
H	10.79768700536050	1.60402198115175	6.12718214293881
C	4.33270413893016	5.06493026591140	5.44743106533659
H	3.39594772037049	5.60180608907609	5.48837398873619
C	8.91142803478841	1.54437327851781	3.33903814801709
C	4.67277610275159	4.37492559987971	4.29198316343152
H	4.00616811557193	4.37032147430829	3.44646568245463
C	3.76468875509422	0.64418409613974	4.50412335938304
C	9.47291710801307	0.28189336674816	1.16554053950513
H	10.40327865202827	-0.11810118756175	1.54169343875213
C	5.10552726306173	1.83888825605460	-1.23863979160890
H	5.52369389256913	1.25191005957932	-2.04314656485383
C	7.88570357704757	5.22538426932490	1.57956471263094
C	3.88551110822517	2.48462340213833	-1.39286165976728
H	3.34080012318988	2.40477638327380	-2.32383374855742
C	6.39202996268042	4.32812494531328	6.41077475195558
H	7.08952251534918	4.28423906681313	7.23432836884626
C	4.11436867549642	3.30468669595850	0.83818182927458
H	3.74185809301078	3.87314698023514	1.67360140557827
C	5.20666844763370	5.04319753185944	6.52161338913854

H	4.97163574756840	5.56894897798652	7.43706070272845
C	3.38161803261272	3.22796104523818	-0.33863535050277
H	2.43615275064316	3.74620197706778	-0.40998310869467
C	8.55093984627654	6.41655493347696	1.10602099763816
H	8.50352861919299	6.44936612165738	0.01687883738492
H	9.59354326684123	6.39858351086708	1.42602603705567
H	8.05688054300574	7.29616240382056	1.52044363155575
C	3.88305729208359	-0.85890775931252	4.26223728019915
H	3.38864812894688	-1.13124878742277	3.32802863335722
H	3.41086030448221	-1.40799517441722	5.07859089263063
H	4.92946838207140	-1.15847854299121	4.20607262341701
C	2.30387685528571	1.08166988410697	4.54772804960800
H	2.23070308691011	2.16168553681051	4.68478988739003
H	1.80103930912266	0.59178349879270	5.38253868751926
H	1.79022311367636	0.80661633210372	3.62520302471110
C	4.49537490268684	1.05560714598199	5.77769311730213
H	5.56295916897896	0.86079040888883	5.69086540896008
H	4.10937622873987	0.47855591322583	6.61935909485360
H	4.33909164110242	2.11170981660328	5.99318588229500

Coordinates of I at triplet state

Co	6.61483835509067	2.74514142026435	2.58108919818879
O	5.49815632041789	0.99354096586722	3.59573110128433
O	4.22555976360132	1.29857070102403	3.62767723583676
N	7.61886106907786	1.61959987036504	1.45064643515667
N	5.32206299364165	2.64173137027036	1.02566616717603
N	5.95988855313466	3.78825298610191	4.19834430492452
N	7.51787891019168	4.56658367881615	1.75860198085354
N	8.06584751285839	2.41990385458622	3.74131870495667
C	5.81266019889094	1.89522446252833	-0.00214653921374
C	6.82876051630883	3.71283212164997	5.24286480670835
C	10.16467227884580	1.38820970206000	4.08521834178222
H	10.98141436987610	0.77829789559275	3.72695028752453
C	7.14434825850067	1.31531689975121	0.24179660326867
C	9.11946574986547	2.69727614922722	5.83054924966532
H	9.13024741528036	3.10266575853229	6.83134924602660
C	7.90353676283708	0.52280778810731	-0.61481009517609
H	7.54347075667586	0.26329886385265	-1.59921165168158
C	8.81019148290674	1.18896797853875	1.90401572352895
C	9.14141732310622	0.06986637705515	-0.17476490232656
H	9.74694043615313	-0.54578848472994	-0.82566994219566
C	8.04983780106567	2.93524602080193	4.97163175246129
C	10.17745465862632	1.92176286518569	5.37194189513049
H	11.01825543463113	1.72675671116463	6.02357334613491
C	4.48672352036368	5.09517001811116	5.54849459670704
H	3.54976284925533	5.62841730008007	5.62291886435776
C	9.07132470250070	1.65623447731099	3.27661015859319
C	4.82624936383964	4.46793414985529	4.35752276540083
H	4.16172111465407	4.51350573097469	3.50893886337530
C	3.42807276125966	0.47703955746071	4.61091470260449
C	9.61023724852740	0.39719009838148	1.09544895690382
H	10.57036225156693	0.04361098164754	1.44248694597909
C	5.09007047243334	1.70127472013723	-1.16857371099055
H	5.50113520130378	1.10522945581544	-1.97024714177857
C	8.04690072740922	5.51061510821215	1.37759966833703
C	3.83287315944914	2.27924553866837	-1.29157350638313
H	3.25415651341640	2.13830066498642	-2.19445947515258
C	6.55605643851580	4.31938324297702	6.45881504306418
H	7.26009960114807	4.24470699764291	7.27459349223014
C	4.11444263219511	3.18848353434125	0.89765384287006
H	3.75098409525559	3.76948419495695	1.73094706829223
C	5.36589346722150	5.01798296519882	6.61615321557285
H	5.13384705472338	5.49225321468234	7.56015900640334
C	3.33566882823450	3.03317305460056	-0.24113236236449

H	2.36187683000502	3.49926860763850	-0.28900464969276
C	8.71652011724021	6.70248490187216	0.89975312771992
H	8.74582690244466	6.69405985634782	-0.19032321260670
H	9.73496989225974	6.72923806553910	1.28871348318049
H	8.17647992106784	7.58664760290397	1.24005633781388
C	3.58364735437680	-0.98597416551528	4.23992087858192
H	3.24817278770777	-1.16197697560415	3.21717186817270
H	2.97063029663563	-1.58492014166267	4.91417256299939
H	4.62036905599577	-1.30425176741103	4.33962913670214
C	2.02156100371449	1.00032212026783	4.38565340969609
H	1.96988096486226	2.07084751947222	4.58773671269414
H	1.34010012498700	0.48708332012628	5.06439820663573
H	1.69722067485892	0.81285192610207	3.36146923249040
C	3.96426832912198	0.80653569266430	5.99409929469128
H	5.00208946001484	0.48939956081009	6.09178242343305
H	3.36740653603912	0.27895782369724	6.73873538992040
H	3.89682482380913	1.87770609009638	6.18799555353021

Coordinates of I at broken symmetry BS(3,1) triplet state

Co	6.43961697377117	2.92082559375251	2.50920322560342
O	5.31928159689821	1.16206038223253	3.32397790128812
O	4.05108802378818	1.40436503347335	3.53627373242216
N	7.61809387204661	1.61198465033931	1.38038929455625
N	5.31080046695910	2.63500801159728	0.73567493216362
N	5.91958288769172	3.80551410430654	4.36450247266323
N	7.32769481844596	4.66577838905136	1.78729185303786
N	7.99394619655137	2.34129263104649	3.78142441798268
C	5.90177200257730	1.87987379298345	-0.21574277615608
C	6.80294711670552	3.61841696166244	5.36811642408917
C	10.05172861039141	1.22416881454241	4.10773124185702
H	10.85400272843212	0.60618851264152	3.73237416015308
C	7.21429732231190	1.30180208656560	0.15165044469723
C	9.02970998942648	2.47745292221077	5.89819558504915
H	9.04019861168482	2.82894939354110	6.91882941229561
C	7.99931350691317	0.48195555613299	-0.65644517564831
H	7.69011111455700	0.21696101379503	-1.65618999126314
C	8.75597989455647	1.14663237202561	1.90082305101779
C	9.19656058441152	0.00284543364663	-0.14247992646174
H	9.82450326057210	-0.63427519007422	-0.75053062126459
C	7.98583662478956	2.79454763856429	5.03216585352676
C	10.06679839907406	1.68926251936032	5.41794527923337
H	10.89053680177702	1.43148672029477	6.06980124515993
C	4.53698385793797	5.09777711588004	5.81287469913281
H	3.63063120069727	5.67101558661657	5.94530742687084
C	8.97483765559164	1.57194398871145	3.30301500584399
C	4.81890643142278	4.52432065214783	4.58212612353944
H	4.14324516687597	4.63956776946208	3.74478508949967
C	3.47529398531728	0.60841789858767	4.68979411874017
C	9.58926193818931	0.32653795779976	1.15168226109721
H	10.51349598571156	-0.05541233159388	1.56002368732362
C	5.28854056932977	1.65973427031952	-1.44179774305789
H	5.77115380935872	1.05598807817493	-2.19584500473398
C	7.79140175739122	5.63151831930713	1.37943848679711
C	4.04453492885505	2.22431649544651	-1.68708597715751
H	3.55331377006910	2.06214092116677	-2.63709380088452
C	6.58629558982791	4.16511458521153	6.62556359107429
H	7.29854965185265	4.00996616544416	7.42182659527650
C	4.11523257456505	3.17343739537703	0.49796224702186
H	3.68471064370427	3.76713668873643	1.29393420816730
C	5.43928753211730	4.91344213350894	6.84955610875335
H	5.25468645635195	5.34411495509815	7.82445781277119
C	3.44318537137412	2.99271605485041	-0.70205189834214

H	2.47426685432055	3.44749395492682	-0.85075941645907
C	8.37626254262168	6.84878794672774	0.86401145300325
H	8.60110504381523	6.72496973737657	-0.19583404779258
H	9.29515624619599	7.06953263606237	1.40832178772929
H	7.67208061428499	7.67161670207563	0.99262948548647
C	3.62588532138385	-0.86320051912607	4.34996136517276
H	3.13689617183918	-1.09314448788662	3.40268514948993
H	3.15365310885744	-1.45268690883406	5.13646439536956
H	4.67688569738834	-1.14347363851858	4.29149973935351
C	2.03093127917669	1.07060140663154	4.70045839964609
H	1.96998609124720	2.14749541111240	4.86135501154500
H	1.50711178087783	0.57010527926708	5.51515301927046
H	1.53381487128546	0.81818458146100	3.76328941640986
C	4.23300184634763	1.00992132182029	5.94307890926950
H	5.29360954802561	0.78233785682684	5.84531262218521
H	3.83364589066883	0.44587412993558	6.78644188911754
H	4.10795081078722	2.07227354619525	6.15006974746604

Coordinates of I at quintet state

Co	6.47226325041127	2.88680187615560	2.56321139754887
O	5.35929635502996	1.24212174259462	3.54230645656212
O	4.08878117372897	1.54882921027083	3.63604989211512
N	7.65564075315954	1.59781798645146	1.41490649369369
N	5.28804919649803	2.51620138102971	0.84547014859661
N	6.02067452125333	3.85200449726607	4.41652652478654
N	7.32165285070265	4.62715834512892	1.75830761174138
N	8.08998973015875	2.38113559210595	3.79397267259991
C	5.87190660794476	1.77164648237437	-0.11972609375028
C	6.92029305332852	3.65616607735319	5.40467138326131
C	10.22276938515723	1.38849243933429	4.04074205468863
H	11.03886367180152	0.80603524594826	3.63884209312899
C	7.22457054896601	1.26105269331386	0.20140102997778
C	9.22820610372123	2.64350232772750	5.84623014668671
H	9.27739581420563	3.03902710582713	6.84950036220260
C	8.02330471294650	0.48039285463250	-0.63211422821407
H	7.69548273203766	0.19785449373449	-1.62121399440723
C	8.83549313005402	1.19724258071308	1.89660892629315
C	9.26147488322909	0.06584985769275	-0.15860682376526
H	9.90037217881707	-0.53987705663807	-0.78687176818992
C	8.12225843549485	2.87459074654329	5.03040221106653
C	10.28022990669191	1.89441013254241	5.33524231309878
H	11.15084003998757	1.70487909790253	5.94860736105665
C	4.62677781452156	5.07118015811985	5.91714448583954
H	3.71083227355125	5.62246971569303	6.07577500764879
C	9.08976700726289	1.65595666747628	3.28312951864679
C	4.90793058127474	4.54134192281646	4.66608942485301
H	4.22126123798804	4.66645379626955	3.83887045139811
C	3.32233523006206	0.65797369328008	4.58714726885062
C	9.68258026352702	0.41675439617530	1.12009217961876
H	10.64051250800467	0.08692502081337	1.49501233115114
C	5.21401149853107	1.50040077193917	-1.31241062174953
H	5.68844684922891	0.90260288650695	-2.07651222632633
C	7.81006718179216	5.58121579312521	1.35174758287239
C	3.93456706852250	2.00310110257308	-1.50934205915288
H	3.40803803345809	1.79970993576032	-2.43224621367580
C	6.70250879037031	4.15512240949085	6.68266502851762
H	7.42215600915950	3.98331384227477	7.46935961229364
C	4.05885387238627	2.99611186511960	0.65468619410737
H	3.63825550302255	3.58299968064282	1.46087688680242
C	5.54054096106944	4.86991357983325	6.94099784243711
H	5.35365769144458	5.26139809280077	7.93197419407666
C	3.34321507032663	2.76258443910382	-0.51064110062283

H	2.34850901270862	3.17010233725886	-0.62261779860437
C	8.43072560505330	6.78404181673399	0.84396092663173
H	8.57373039738085	6.69513771003861	-0.23346134132974
H	9.39738501474135	6.92510943500129	1.32922444224668
H	7.78981051194314	7.64058446413528	1.05659430881749
C	3.47580478253411	-0.77556539998845	4.11522962016717
H	3.11970268413833	-0.88475423718176	3.09016649600242
H	2.87845548202998	-1.41997462543747	4.76093514955965
H	4.51512485665803	-1.09560113482934	4.17256012628973
C	1.90840512115800	1.18680172020791	4.43584669617422
H	1.85877633937978	2.24188827983964	4.70731780280408
H	1.25029873480305	0.62763257987176	5.10116047677156
H	1.55438462671176	1.06186466034909	3.41209671426024
C	3.89788086651317	0.89947358933237	5.97282498701710
H	4.94223142347588	0.59218776041281	6.01650972998438
H	3.33131873540916	0.31119996897164	6.69530142207445
H	3.82152532452945	1.95258659546373	6.24506228276699

Coordinates of II (reaction complex between I and styrene) at singlet state

Co	6.53423896962266	2.67921524075266	2.69194215725504
O	5.71545372931251	1.10103241017309	3.23262883102735
O	4.31933728304975	1.39434164997955	3.43786285311751
N	7.56809609864317	1.61155348441501	1.54104072083779
N	5.30939025099348	2.70276662171485	1.09762566663409
N	5.85198373319892	3.68418207269206	4.28361634533220
N	7.35626012311184	4.32781624958503	2.03697964474245
N	7.99169577150256	2.39058757687392	3.84219146771585
C	5.76083602043819	1.90079665433703	0.09725994622282
C	6.74353119582925	3.69868773166971	5.30926866153644
C	10.16505513505535	1.52322083705096	4.12752136699580
H	11.00601732625785	0.95553824075004	3.75630401992180
C	7.06441354417281	1.27278722572340	0.35678701585773
C	9.10912032020499	2.84358538077587	5.85912765696747
H	9.13654844826528	3.30205073770541	6.83615406813151
C	7.79060496495575	0.41837377997159	-0.46863990098130
H	7.40963620476680	0.12264418914101	-1.43468732557030
C	8.74729650862210	1.17032028305303	2.00744646053511
C	9.02016337094446	-0.04883777694532	-0.02062171434962
H	9.59754799572575	-0.71465681243746	-0.64708844271422
C	7.98994307873276	2.96970465119505	5.04121821769648
C	10.19595005035250	2.11879979622778	5.38627024294693
H	11.07638385759943	2.01324211073052	6.00511626983250
C	4.32702835835559	4.94385508641330	5.60768127914537
H	3.35730938596511	5.41431958281147	5.68274655080943
C	9.02306572353448	1.67866130474961	3.35874410742011
C	4.67526930586351	4.28242334656870	4.43785707439524
H	3.98062210271075	4.22199850546087	3.61722342614949
C	3.81642702860424	0.64467327563699	4.57510664176682
C	9.51656540680978	0.32417345676502	1.22598414484896
H	10.46706003650341	-0.04417824832429	1.58330629452901
C	5.03732089730669	1.72887027012495	-1.07124610959528
H	5.41214480873438	1.08392945190331	-1.85248052147426
C	7.81841717203634	5.30430798676240	1.66193732454168
C	3.83145091022198	2.40123548137766	-1.22476051845366
H	3.25179421182095	2.27945019112259	-2.12972081562976
C	6.45927078606360	4.33874843466368	6.50398415857029
H	7.17957790399644	4.33319259431520	7.30881426773911
C	4.16330304095035	3.35599653069216	0.93861709044676
H	3.84922731489675	4.00254290941657	1.74150105042774
C	5.23406829565761	4.97571509019483	6.65383475222387
H	4.99286637087571	5.47957400472163	7.57991318191624
C	3.39113713401979	3.23329774707468	-0.20879606991880

H	2.46545111132689	3.78495959943542	-0.28810792114943
C	8.39719379179081	6.54014353775936	1.18991284966607
H	9.16043576126199	6.32018578799543	0.44249693801131
H	8.84896339300805	7.07144454940905	2.02845556776966
H	7.61659004500022	7.15756354546897	0.74369906701801
C	3.85969797169162	-0.84430694436685	4.24204146815549
H	3.28862505967068	-1.04254548217442	3.33304326611925
H	3.43311353160114	-1.42911328324858	5.05809632925938
H	4.88670147779739	-1.16977393999957	4.08974789927375
C	2.37887109598263	1.14335100003057	4.68342104031469
H	2.35655015424735	2.21188046610781	4.90223891015532
H	1.87060029234190	0.61413265418839	5.49057788159309
H	1.83498830433967	0.96140287508661	3.75507523741367
C	4.60028501441147	0.95547534559492	5.84513016304492
H	5.65422647650891	0.71842576180838	5.72116554545625
H	4.21323174787030	0.35165542346651	6.66624244310527
H	4.49799489054542	2.00444847024130	6.12066632132607
C	7.55649666429584	-2.37590610918658	3.19841168051293
H	8.05650193958331	-2.34522631032099	2.23919022269386
C	8.08451226143239	-1.76724416051314	4.26100434838895
H	9.02165275147682	-1.23339431189047	4.13796500588640
C	7.51958040860182	-1.70327698271665	5.61437377370066
H	6.61450795714791	-2.90895912050196	3.24125312569222
C	8.05227976223525	-0.78558326707522	6.52712525490662
H	8.88029705205705	-0.16063020272814	6.22152184173052
C	6.45607499955703	-2.51225940480867	6.03600177382183
C	7.52982733713293	-0.65842025568466	7.80824045189094
H	6.03258003048991	-3.24262749409621	5.35923815100638
C	5.93622009306101	-2.39045733841275	7.31575836679067
C	6.46689228953093	-1.46055360170179	8.20778725128437
H	7.95318576323040	0.06663425014953	8.49217715573107
H	5.11202787913095	-3.02306101835755	7.62110214730999
H	6.05560251535167	-1.36659337654487	9.20487490256857

Coordinates of II (reaction complex between I and styrene) at triplet state

Co	6.73950642493781	2.72607782563366	2.73767536321649
O	5.61463190579041	1.00068284877969	3.78402356985784
O	4.35327706587121	1.35429034137358	3.78858913238092
N	7.74351549941753	1.58968693136319	1.61844289308461
N	5.40954361674835	2.53276932424479	1.22088039565472
N	6.09391666549976	3.78503752741928	4.35291016594648
N	7.51574508303196	4.57139083656415	1.84536313208185
N	8.24663002823391	2.50881079390970	3.85108666979936
C	5.86743298303911	1.71364966196351	0.23509609215503
C	7.00816165190556	3.79008933576211	5.36073726272015
C	10.43063675333797	1.64296561152597	4.11808219234204
H	11.26624743839769	1.07034207569194	3.74217281613656
C	7.22208129422630	1.18169177136186	0.46054257793361
C	9.39360487366428	2.96340093854647	5.85908308560734
H	9.43112147553292	3.41953014273273	6.83692062341635
C	7.96412561069966	0.34795408573231	-0.37178774552268
H	7.56359198868298	0.00218934569008	-1.31302490452280
C	8.97104588751923	1.23318101060004	2.03764014152062
C	9.23738233981517	-0.03130618688939	0.03481613015714
H	9.82935126076188	-0.67895217627842	-0.59713764234774
C	8.26288615276130	3.08291868159597	5.05504334933586
C	10.47781702371024	2.24192351155967	5.37500053948904
H	11.36548612616315	2.13848561988089	5.98390975939007
C	4.59357039337937	5.02039666187940	5.73948372763833
H	3.62612538257770	5.49034537898328	5.84417812079853
C	9.27724825417595	1.79319808020936	3.36466076222175
C	4.92422866902472	4.38996340657845	4.54744371990471
H	4.22126869579915	4.36755111120926	3.72932306490683
C	3.49106604048318	0.51654050097404	4.69704207798116
C	9.75800952164956	0.40961629624708	1.24907408635878
H	10.74427104135977	0.11183733488471	1.57415218095395
C	5.09870850491101	1.41990810138813	-0.88007024849358
H	5.48171118875543	0.76364132047931	-1.64788956383595
C	7.87393072113196	5.58074395977291	1.43421592901177
C	3.83190748389018	1.97808892133658	-0.99560528419811
H	3.21660940168341	1.75874261108712	-1.85774920883752
C	6.74621290293811	4.40373677340341	6.57557781997975
H	7.48478290504806	4.38993473148252	7.36373888098823
C	4.19634662772823	3.06722491944543	1.09702076415292
H	3.86588415217182	3.71609607830102	1.89327640451280
C	5.51972896978606	5.02631035329508	6.76943262555586
H	5.29556522460801	5.50460134307052	7.71334029360454
C	3.37397250769568	2.81780828206736	0.00668857091706

H	2.39697744642899	3.27719750385972	-0.04088538959957
C	8.32346248940454	6.85655577955516	0.91751039688914
H	9.07725403891670	6.69409184488962	0.14639741881366
H	8.75512379986915	7.44675586348185	1.72657642065325
H	7.47794875929547	7.39536540059749	0.48851395973770
C	3.52646489863163	-0.90835232041181	4.17717693816452
H	3.15432011573794	-0.95335909924927	3.15293131158240
H	2.89028617505122	-1.53042796655691	4.80757138317544
H	4.53867524089472	-1.30513626935871	4.21161803480333
C	2.13368143989968	1.17488242557956	4.53074158850132
H	2.16859349021906	2.21770646279395	4.84797626318050
H	1.40654712163309	0.64848866923343	5.14939458180708
H	1.80415292144955	1.12758127275756	3.49226831468037
C	4.04926522686675	0.66162405809266	6.10214159159673
H	5.05342245106771	0.24895676187987	6.16513958555857
H	3.40738439799646	0.11803138660703	6.79574066856641
H	4.07205802179034	1.71186308418053	6.39683711027926
C	7.33156019510992	-2.11864584068491	2.96973739104590
H	7.78188901490556	-1.99104419620893	1.99411106709002
C	7.84746146327807	-1.51633621894097	4.04141394018252
H	8.72094052268404	-0.88721611343551	3.90976959380238
C	7.34855548077802	-1.57761413099479	5.41928958576143
H	6.44627539333884	-2.74074816223815	3.02207064129459
C	7.82857675184124	-0.65132708571287	6.35273334684632
H	8.56455239300708	0.07808651519707	6.04088532839006
C	6.40333792436679	-2.51901144466790	5.84807926730885
C	7.36975355785449	-0.64839773287947	7.66406673130825
H	6.02757378826071	-3.25992546651747	5.15443180124945
C	5.94664318585360	-2.51994439857983	7.15782646108790
C	6.42463215813842	-1.58333286009353	8.07232043179881
H	7.75016642613627	0.08384847675364	8.36529288739315
H	5.21504378823248	-3.25505037234409	7.46942200474031
H	6.06372218251088	-1.58595185144403	9.09299901835234

Coordinates of II (reaction complex between I and styrene) at broken symmetry BS(3,1) triplet state

Co	6.53371093705720	2.88000163071017	2.69575167333539
O	5.42919376555726	1.14818096346847	3.58727969107097
O	4.15955352817003	1.44447506116527	3.70381322779874
N	7.74903427268912	1.59499655941488	1.57754271683193
N	5.36386117539918	2.45424091530781	0.97138297714676
N	6.02620033113503	3.78325367567845	4.55312248168647
N	7.30485752214266	4.65161001542848	1.89997583045711
N	8.18686369568550	2.47695027137479	3.91503476463410
C	5.93805813375190	1.63421716375182	0.06524712042404
C	6.96366898558920	3.69611141023078	5.52026696651784
C	10.39349211196081	1.65613828225469	4.12913544795722
H	11.23919003076327	1.12137134430663	3.72269805651326
C	7.30262011734491	1.16813365463286	0.39966716581649
C	9.35315310434552	2.88218925615157	5.92750823525516
H	9.39185885748117	3.30102621655004	6.92151634195017
C	8.10141012098993	0.35038457810859	-0.39692673034008
H	7.75740209739142	-0.00914800876374	-1.35486351756809
C	8.95311412254196	1.26463533020695	2.04935080429767
C	9.36132710402419	0.00357338475347	0.07031876708844
H	10.00036390443409	-0.63044478241838	-0.52927802129805
C	8.21359863196049	3.00408412631451	5.13566409241836
C	10.44770950672845	2.20745990559135	5.40478830886094
H	11.34664719677863	2.10302006289526	5.99703895485917
C	4.57661290896387	4.93268485337681	6.05514923858855
H	3.62213586786044	5.41056298715606	6.22313718723640
C	9.22139501541911	1.80788207179779	3.40072510998497
C	4.86715766736244	4.38596338030350	4.81420158728465
H	4.14933275908349	4.42455719726911	4.00558465581234
C	3.45052554557951	0.64643112903739	4.77639814549408
C	9.80502380634150	0.45870707206644	1.30693380324385
H	10.78026776283147	0.18253908047218	1.67981022320565
C	5.26672023907391	1.25771871420483	-1.09045030028749
H	5.73419187285520	0.59830615494555	-1.80640038398451
C	7.74172227624099	5.61747229792205	1.46375697710812
C	3.98297327230184	1.73601270836301	-1.31320849397951
H	3.44576811464528	1.45116445155632	-2.20787403707448
C	6.74021260891205	4.21710331737353	6.78741501563207
H	7.49417294745702	4.13220762801705	7.55571949226073
C	4.13148548483660	2.91173139881156	0.75452002372386
H	3.71748920258842	3.56406002427583	1.51229986625181
C	5.53090195627932	4.84238125305817	7.05710192556133
H	5.33882675141710	5.25067068917470	8.04017287250351

C	3.40206158475985	2.57797082474235	-0.37723320125911
H	2.40474274233766	2.97185956233479	-0.51134429205984
C	8.29447935801542	6.83453464024513	0.91346881431095
H	9.04711556755360	6.58483739343324	0.16470735083720
H	8.75365774671218	7.41978231053475	1.71078846407897
H	7.49827443249428	7.41606846094225	0.44731069703390
C	3.42029709023581	-0.79495404787998	4.30090860572704
H	2.88534229038302	-0.87737900478852	3.35423255532033
H	2.90761357298784	-1.40280780417587	5.04683939680366
H	4.43085450259971	-1.17947197630615	4.18026315667114
C	2.07875412885008	1.29378235554194	4.79436345774633
H	2.14903687483114	2.34396983145274	5.07951022271833
H	1.45660950043696	0.77747651131414	5.52588027654832
H	1.59865185661620	1.21733232492168	3.81816131564657
C	4.20827468930066	0.83561339507584	6.07746295910682
H	5.22096438294099	0.44718736744686	6.00470740443087
H	3.68712937523410	0.28861014331925	6.86299456681545
H	4.24319422449726	1.88912101254362	6.35501498168180
C	7.22001926240655	-2.07614283179009	2.87763357308900
H	7.65963269493306	-2.00166953031800	1.89167660230018
C	7.83525785234693	-1.55292923131319	3.93851594888838
H	8.78029033690921	-1.04208939971705	3.78745499730326
C	7.35953158977249	-1.55705918090025	5.32643906159981
H	6.26320293883701	-2.57887178165372	2.95067047484852
C	7.93534110888925	-0.66734102349211	6.24008508396689
H	8.73067146586173	-0.01394728111168	5.90807433375458
C	6.34467173953604	-2.40805049477596	5.78241028902633
C	7.49839290283166	-0.60576967995235	7.55698922360574
H	5.89610059404437	-3.12241213977648	5.10442239828493
C	5.91212809173182	-2.35384925090511	7.09896096745402
C	6.48267041708686	-1.44967807826378	7.99202801890343
H	7.95192452466731	0.10039828176973	8.24119356740730
H	5.12546768924831	-3.01917439505939	7.43217991747662
H	6.13902755613269	-1.40725470973682	9.01770054564840

Coordinates of II (reaction complex between I and styrene) at Quintet state

Co	6.60268032493971	2.84940830710159	2.75774743190677
O	5.50529673704335	1.20803623771466	3.76238275320721
O	4.24424607519796	1.56252317969128	3.82036872324936
N	7.80834013244886	1.58385275348424	1.60563050517403
N	5.38415742159397	2.37893179875951	1.07928488825383
N	6.16053434737757	3.82204991826387	4.61820381247450
N	7.29357281877076	4.62110322256249	1.87352058726454
N	8.29407871154071	2.47395931814812	3.93696026951781
C	5.95681049129272	1.59318074019637	0.14141655804394
C	7.10666509858096	3.69618041835053	5.57327202235709
C	10.50617204365912	1.65307856493962	4.10499745161294
H	11.34368381096128	1.11937654935471	3.68039114148835
C	7.34003083182702	1.15507891911197	0.43568508834634
C	9.50355383962070	2.87943880474770	5.92511744042943
H	9.56412152065250	3.30158669818953	6.91683066521041
C	8.13517656518331	0.35796855234755	-0.38589506888359
H	7.77442998247373	-0.00153352250735	-1.33782926766122
C	9.02856292919144	1.26963560648460	2.04920669776933
C	9.41219385258462	0.03017117869217	0.04920367846413
H	10.04817345573952	-0.58755441445573	-0.57019199224546
C	8.34666674900127	3.00243439529428	5.15788371441782
C	10.58645578474589	2.20187293366601	5.38102254544437
H	11.49708604071314	2.09686121389266	5.95515315565856
C	4.73539097696140	4.93549745525558	6.17111489594978
H	3.78668262798957	5.41586902335605	6.36405115415632
C	9.32052360291802	1.80815763478382	3.39853714223685
C	5.00998457990951	4.42808844681704	4.90912712000878
H	4.28576304132369	4.50041013996949	4.10841467212704
C	3.40402224062983	0.67133219106437	4.70204782669550
C	9.87571235092799	0.48264003724614	1.28028511039412
H	10.86359649344196	0.21899393559692	1.62852756932124
C	5.26522562020905	1.22504763190219	-1.00567132115903
H	5.73039246303017	0.59350756650796	-1.74790971925320
C	7.63146201494742	5.59127892391536	1.36488476417203
C	3.96407259736246	1.67546978623159	-1.18541694876830
H	3.41112734202592	1.39598217290559	-2.07226698528272
C	6.89964918685077	4.17776206212757	6.85954467569758
H	7.65928308276693	4.05869094594147	7.61792914120582
C	4.13553508889031	2.81141435205372	0.90377534728214
H	3.72579435789847	3.43665968899882	1.68606743950394
C	5.69717763519394	4.80273118522020	7.16166440803049
H	5.51606537190825	5.17736762685920	8.16022214144056
C	3.38650585797086	2.48321742626650	-0.21696844156872

H	2.37694827706243	2.85555809351086	-0.31816503088609
C	8.05699011129686	6.81333310029564	0.72053414425712
H	8.85255577663289	6.59053655207224	0.00856805889733
H	8.42455859713722	7.51396068037414	1.47093904214661
H	7.21184071092496	7.25682801132803	0.19254091944539
C	3.44263287951399	-0.72554819596760	4.11170448877295
H	3.05784295928373	-0.72288818001589	3.09130021444285
H	2.81771584280472	-1.38073047541784	4.71923810793054
H	4.45685687980660	-1.11818792368283	4.11529602233690
C	2.03914486239633	1.32493973661549	4.59079216891766
H	2.06965436131824	2.35126591906172	4.95803451204162
H	1.32909954899808	0.76159384559404	5.19662312132485
H	1.69080792478934	1.32366702804122	3.55743658518400
C	3.98833701792809	0.75456804466654	6.10129082270277
H	4.99888090904386	0.35395017026742	6.12431685757114
H	3.36813059384798	0.16602116325140	6.77741631425443
H	4.00205156846950	1.78816436715932	6.44883065845550
C	7.21568679496451	-2.01569177636572	2.89236736740691
H	7.66931246671271	-1.91868656437732	1.91476705359639
C	7.78708925559106	-1.46874674325467	3.96553116017889
H	8.71073204749450	-0.91546560205085	3.83472147257970
C	7.28947315921048	-1.50140490219876	5.34528052973863
H	6.28171054064661	-2.56186868027788	2.94617758842326
C	7.82251480566174	-0.60358974840455	6.27785024000954
H	8.60266637761985	0.07844430073823	5.96678629811624
C	6.29577516886424	-2.39034459676590	5.77643808033940
C	7.36464986752015	-0.57287532073962	7.58916647532669
H	5.87968000461028	-3.11031824658949	5.08376794709009
C	5.84291702150563	-2.36649491283987	7.08728253951619
C	6.37140824534653	-1.45558700111437	7.99978457081828
H	7.78521144375657	0.13863638885289	8.28882671638699
H	5.07361368038594	-3.06108698888051	7.40089669328696
H	6.01204420055108	-1.43740114993701	9.02083546569568

Coordinates of III (transition state between I and styrene) at singlet state

Co	6.57918203420111	2.47445707234386	2.81539538690115
O	5.29443754955647	0.80296424726084	3.80805625634166
O	3.95686993194187	1.14182475303302	3.61054816174761
N	7.82946975505847	1.32094630817270	2.00934816612291
N	5.56349210282601	2.06874866958831	1.11424034421603
N	5.67763479272025	3.75612185523167	4.08537538510239
N	7.49283915636482	4.23288888094349	1.85334359684594
N	7.83440833238911	2.41479801486060	4.21561029538350
C	6.27515266725829	1.27444683160644	0.26883688340969
C	6.35549810546657	3.88627725767928	5.25703697769042
C	9.88995672855336	1.58329103577199	5.03311764178273
H	10.78586481102336	0.99688960574914	4.89706879310894
C	7.59798352342103	0.87134039006432	0.77495787412294
C	8.49012967045373	3.00672250083535	6.39881416814304
H	8.30625026065232	3.52861387100932	7.32613312863181
C	8.56567043085743	0.10475662869649	0.13167874301007
H	8.40304885168561	-0.27138017285457	-0.86736937561239
C	8.96658480728853	1.07264156116240	2.68377615259254
C	9.75259288671877	-0.16397874247323	0.80364263604002
H	10.51754476074855	-0.75627370484660	0.32038669491710
C	7.58892424855047	3.08655281229860	5.34110819209521
C	9.63935078223884	2.24316340911591	6.23323246436985
H	10.34971135925784	2.16376343337105	7.04456321804429
C	4.10497212330949	5.38545698374377	4.83567786456124
H	3.21472321403518	5.96028758960623	4.62420318916393
C	8.94700373648764	1.68333865521458	4.02309575389035
C	4.58867779815688	4.49491510954335	3.88694148054071
H	4.08436290345211	4.37589762481959	2.94050558683181
C	3.10822399440335	1.03934402650425	4.81163197225859
C	9.96767251758814	0.31932436266616	2.09211256002044
H	10.88646601520955	0.10943029819541	2.61980955825869
C	5.76243503813033	0.87323264830426	-0.95454756219840
H	6.34575341274607	0.24088076508605	-1.60798824716424
C	7.87701936583117	5.23447669801726	1.44664238581318
C	4.48932008968968	1.28896931776687	-1.32356343418435
H	4.07205630745826	0.98569533011074	-2.27434545107958
C	5.92347552116345	4.74932511774835	6.25182899539124
H	6.47588939774787	4.83262390044195	7.17652013532894
C	4.34075198929519	2.45310670445477	0.75624489078265
H	3.79660864100842	3.06227665809345	1.46054791405757
C	4.78047555665258	5.50926540923158	6.03921609631198
H	4.42927465962862	6.18904163978257	6.80368611419137
C	3.76525859091408	2.08872836138640	-0.45409415085621

H	2.76858751734163	2.42967048417870	-0.69563438089868
C	8.35695116455688	6.50216408784339	0.93732830468252
H	7.52814076708045	7.05391910960232	0.49239385170106
H	9.12258065435568	6.32748252529005	0.18076828720393
H	8.78233407666764	7.08612030671317	1.75425454333903
C	2.77327322744286	-0.41926905135184	5.11184122011573
H	2.49257532090935	-0.94859093474328	4.20066362357208
H	1.92968004409931	-0.45347544639218	5.80306826068649
H	3.60567570357972	-0.93486341853607	5.58920872377232
C	1.86157216227833	1.79345864412585	4.36029893523650
H	2.10644548853433	2.82410129393529	4.10373456036869
H	1.13140189934821	1.80103142224628	5.17019119559255
H	1.40966253799239	1.31059207919363	3.49229529929789
C	3.76449698459556	1.70542945896079	6.01042180149275
H	4.75949292078147	1.29913705710909	6.18236859529646
H	3.15788043655751	1.51707887631717	6.89724695530575
H	3.83545902649397	2.78127275195250	5.87509774656023
C	5.56682628364933	-1.10423128626215	3.40919432322655
H	5.05389420976609	-1.10769800562383	2.45814323204983
C	6.92850982040289	-1.34963998682910	3.43530715223735
H	7.44925583297864	-1.46789617765755	2.49275111479940
C	7.73162007045312	-1.41558880290513	4.62520240174142
H	4.97295701838594	-1.36158478777802	4.26807424747771
C	9.05871495114891	-1.88102621842103	4.54418166376367
H	9.44080345135309	-2.21152554224633	3.58660159201492
C	7.25291768929654	-0.97870752724173	5.87763540297366
C	9.86851674445700	-1.91850320828048	5.66635031402291
H	6.24950004566879	-0.58475903987837	5.95667115426728
C	8.06962384202918	-1.00995528781003	6.99378589713987
C	9.37945807396430	-1.47985796283145	6.89567086061579
H	10.88611160444822	-2.27874109166344	5.58526974321943
H	7.69301790190655	-0.65410346923646	7.94427231706774
H	10.01755410533329	-1.49361457111815	7.76985362312873

Coordinates of III (transition state between I and styrene) at triplet state

Co	6.58685586786617	2.49699733536190	2.84409271305467
O	5.25206696419653	0.85073331204143	3.99605034381823
O	3.96573088227691	1.19745666589272	3.62584365723339
N	7.82612657781694	1.35515187252974	2.01311399247742
N	5.53943600976602	2.08055191504113	1.17040015953369
N	5.68737004793583	3.74746008545547	4.15342328956937
N	7.48527415855285	4.25974115506850	1.87072757808870
N	7.86326015536841	2.43547366854315	4.22282408694739
C	6.23078342103872	1.27989590828939	0.31599151549025
C	6.36872666544388	3.84284276272122	5.32528403038752
C	9.95174139681981	1.63680337858084	4.98475788341729
H	10.85697261771391	1.07268400171094	4.82081828142932
C	7.56830921791623	0.89165561832674	0.79057376069601
C	8.54664304434460	2.99854278531047	6.40439394633467
H	8.36734135041054	3.49655759274099	7.34549790860426
C	8.52440080444722	0.12800597342550	0.12884210650992
H	8.33901358176274	-0.25873926352320	-0.86200836220411
C	8.98085985846101	1.12123145566861	2.65955018012814
C	9.73077390831041	-0.12184169661903	0.77127974060510
H	10.48889524533993	-0.71059784092227	0.27326964866472
C	7.62360329295590	3.07527881558840	5.36626484988209
C	9.71172929920113	2.27107918702628	6.19980087814233
H	10.44167752327329	2.19686925895683	6.99390398254574
C	4.08599932695551	5.30866843110349	4.98362605515048
H	3.18358134350382	5.87414713614619	4.80104253068530
C	8.98424201960454	1.72907717321602	3.99893946135866
C	4.58459988057561	4.47342598812042	3.99354467755936
H	4.08021086477759	4.38873780601123	3.04337558655200
C	2.95514969362985	1.04365780948209	4.68956351840219
C	9.97415140478349	0.37383212024644	2.04901780376056
H	10.90786611091178	0.17639780335272	2.55439113948486
C	5.68919467820946	0.85975130110592	-0.88761489907853
H	6.25809386107490	0.22036177096119	-1.54676147210485
C	7.96082474774270	5.18119564952115	1.37968504521083
C	4.40529937492814	1.26462786528369	-1.22817621123339
H	3.96364477741119	0.94607157966946	-2.16273962784608
C	5.92249789189694	4.64643416516478	6.36187263734694
H	6.47887139690561	4.70036154469657	7.28620229397460
C	4.30742626528911	2.45668770755702	0.83762270663554
H	3.78042677427225	3.07542503660633	1.54615311777152
C	4.76141616475873	5.38804314442789	6.19031840257575
H	4.39746317641142	6.02171067894126	6.98762660007226
C	3.70200301083334	2.07429705585144	-0.35159939487345

H	2.69827324508122	2.40911419895992	-0.57098883963947
C	8.55975730442318	6.34570011982819	0.76109525931643
H	7.81276085166498	6.86526038006517	0.16015266396320
H	9.38624418202738	6.03566018617515	0.12103793570072
H	8.93341424517310	7.01837103812946	1.53363976696236
C	2.50291737317054	-0.41067552473627	4.78665534529929
H	2.29835683847876	-0.81671463040985	3.79536302614608
H	1.58474250078301	-0.45860795061822	5.37428843953323
H	3.24347120775945	-1.03421376165422	5.28320002135513
C	1.82376446158544	1.91784564207153	4.15786202059403
H	2.14143745743896	2.95600027274179	4.06937512648342
H	0.97827477489768	1.87075587093978	4.84464979314342
H	1.49301486388684	1.56605688213161	3.17932121452811
C	3.48892464058987	1.55273916878799	6.01849709663378
H	4.34956173672208	0.97047068375121	6.34247994954232
H	2.70571742741057	1.45954906102112	6.77216192022651
H	3.77511285620454	2.60062911824600	5.95337000244758
C	5.57171908264471	-1.09768813385476	3.66523828723314
H	4.97411296725563	-1.14081329285850	2.76684212012275
C	6.93194108730959	-1.29712914534359	3.57940971650312
H	7.37973569501742	-1.40398457588424	2.59876355193571
C	7.82520168546309	-1.34242142262924	4.70696800653050
H	5.06679599930020	-1.31933975596788	4.58940636212794
C	9.14199562920338	-1.80866134666705	4.53563173638180
H	9.45609694820808	-2.14447001204983	3.55560903680380
C	7.43622711196273	-0.89577638503612	5.98516796463845
C	10.02720375230074	-1.84427745684986	5.59911230928684
H	6.44118967676630	-0.49916894352428	6.13309904131445
C	8.32813995937151	-0.92371713506477	7.04241848307015
C	9.62518875121329	-1.40013786165125	6.85708694983130
H	11.03542947388461	-2.20872923107300	5.44953262786923
H	8.01986065035051	-0.56102761657825	8.01457354412226
H	10.32137090675359	-1.41432415507910	7.68573337723068

Coordinates of III (transition state between I and styrene) at broken symmetry BS(3,1) triplet state

Co	6.33638873401363	2.48185474822634	2.85262238083430
O	5.19127664483178	1.00078106914005	3.94304166954835
O	3.89368802843564	1.35957751586056	3.59391199685207
N	7.87424767763475	1.35220028896857	1.97273028871353
N	5.48287707416190	1.76478493938236	1.00999852127818
N	5.72655531333627	3.86495171400735	4.37353585659671
N	7.00318357714445	4.17480631818670	1.73169001888844
N	7.85751100636796	2.38550939715788	4.29318704246029
C	6.33944969258011	1.12059599870707	0.19123648692626
C	6.44338098005483	3.82420491773098	5.51438648431593
C	10.01446355322401	1.65451896310657	4.93982644351042
H	10.92892032633177	1.12665737393847	4.71667497350940
C	7.67991218914685	0.84912608919617	0.75542134409750
C	8.65904040969334	2.96099339609697	6.44076147725575
H	8.52450062606234	3.45982810665745	7.38872386223730
C	8.68269900041484	0.10965177731628	0.13336305453502
H	8.54069874532111	-0.31073040568411	-0.85065655909596
C	9.00398650811805	1.14865992342040	2.65298886459717
C	9.87576495960242	-0.09021797745745	0.81463222576261
H	10.66914142148591	-0.66208575129000	0.35271841217098
C	7.68351120153131	3.02131943854849	5.44912526008176
C	9.82652318450706	2.25944022469815	6.17528404513630
H	10.59929195893808	2.19601978378204	6.92908320066925
C	4.20368871257356	5.39734957141814	5.37675105951439
H	3.31913184421250	6.00996940218589	5.27690738676896
C	8.98809212027336	1.73517466393424	4.00880864968607
C	4.64523792453165	4.63799377081925	4.30311639418184
H	4.11625542129478	4.64329272675442	3.35890888696005
C	2.89186767983982	1.21207277761777	4.67198346499191
C	10.04847520484677	0.42537112964068	2.09259482370545
H	10.96436329477404	0.25554151015312	2.63851810436675
C	5.96263710050228	0.73818218511502	-1.08977009897683
H	6.65859226830999	0.22667072975927	-1.73755600511479
C	7.35093567506060	5.06863440404199	1.10365802826810
C	4.67913805610739	1.02795328624781	-1.53082640680610
H	4.36950111564225	0.73904349493249	-2.52631612092002
C	6.05400533603238	4.54554709841548	6.63511945470183
H	6.62895531584659	4.49395538571989	7.54756799914158
C	4.25113667155795	2.04027885529305	0.58443840924683
H	3.60388095347042	2.55042655630558	1.28417440576268
C	4.91715362856583	5.33868657832618	6.56470183319864
H	4.59782432055551	5.90700410542073	7.42792888402905

C	3.80535362110713	1.69006231951274	-0.68191307541223
H	2.79671797433094	1.93275355027042	-0.98495797969812
C	7.78773839075284	6.19659225100662	0.30971637654796
H	8.85157742790559	6.09811649570064	0.09116049120816
H	7.61515586998460	7.12096392413522	0.86179494200395
H	7.22708005386492	6.22227134825066	-0.62530219167129
C	2.33167957982389	-0.20788729710417	4.64990090253914
H	2.06685461525055	-0.49779158995477	3.63254343950221
H	1.43026837913591	-0.24382063852426	5.26373724904166
H	3.03588460485161	-0.93113157417930	5.05480236948824
C	1.82277437742941	2.20791772954220	4.23334647159460
H	2.20795811227360	3.22624386308952	4.24571838266808
H	0.97379986826216	2.14831977489330	4.91480836758346
H	1.47283316053316	1.97469189417665	3.22652349843868
C	3.48241381269756	1.56513336075839	6.02552150172435
H	4.28596830522444	0.88259652006710	6.29518937150600
H	2.69663463329561	1.48753737653600	6.77813274329277
H	3.86510019244269	2.58318209441275	6.03720250398283
C	5.47567328434217	-0.98791383513261	3.70320881129994
H	4.84362310120977	-1.04813447226702	2.83013186946157
C	6.82631432105684	-1.22219850022548	3.57925007312138
H	7.23809094912686	-1.36817491687240	2.58780145126195
C	7.75319355427809	-1.27848492665332	4.67762819397691
H	5.00070541371425	-1.13979451709375	4.65744507311575
C	9.04828065358539	-1.78721764122535	4.46628353512275
H	9.31901842456220	-2.13753846714077	3.47860403437107
C	7.41975501971480	-0.81037874502648	5.96407119836891
C	9.96579328325710	-1.84459368752966	5.50075500167576
H	6.44349343819500	-0.38091893985692	6.14190616409738
C	8.34347549496964	-0.86052971137139	6.99154170128139
C	9.61790788825046	-1.38060963496813	6.76733899148847
H	10.95669426562869	-2.24156328287420	5.32183035000953
H	8.07971914543974	-0.48099838489458	7.97017128234256
H	10.33938332656209	-1.41223782125704	7.57354040107387

Coordinates of III (transition state between I and styrene) at Quintet state

Co	6.35140725035269	2.44712278971293	2.87117832514956
O	5.21114274906661	0.99204560264493	4.03987304162662
O	3.93541451991681	1.43731663256959	3.68209709631544
N	7.93439429524110	1.36586952636877	1.99832086159651
N	5.52924392355328	1.68913701723295	1.03172563567103
N	5.74080164268777	3.87338099579897	4.36357110024838
N	6.95020372369812	4.16022800269682	1.71144126072204
N	7.87696879601399	2.40127980853027	4.32095308704615
C	6.41916425609548	1.10481054040207	0.20173036440046
C	6.44719681879126	3.85210677731659	5.51219224436105
C	9.99942269679221	1.62301291350370	5.02599013732505
H	10.91178053663020	1.08190900429076	4.82687223790281
C	7.76873773615644	0.88122493139449	0.76791232174926
C	8.61666699325176	2.93186527867651	6.50238682244647
H	8.45973292380530	3.41682690988916	7.45422527184077
C	8.80259569619838	0.18710563112611	0.14341726724603
H	8.68705794992303	-0.21639473579104	-0.85123293913231
C	9.05912995494654	1.17800779591969	2.69323564372670
C	9.99171563038955	0.00593267102873	0.83830876099948
H	10.80836590906259	-0.53124985045025	0.37513784330256
C	7.67521521197638	3.02656884273645	5.47950572648019
C	9.77889440342742	2.21009365460421	6.26572353938208
H	10.52287904809792	2.11726574249767	7.04515553268010
C	4.23603089832553	5.46116601242504	5.30739824955376
H	3.36258453497198	6.08518214520835	5.18237649968835
C	9.00728460761364	1.73844697223326	4.06070185098167
C	4.67244424575351	4.66151113489772	4.26087294377357
H	4.15047611261064	4.64562783056754	3.31280330378306
C	2.91600988511555	1.26897031128797	4.73517907249188
C	10.13069084819240	0.49457130236330	2.13162725032198
H	11.04402396176058	0.33671601363819	2.68564736593738
C	6.06082097830473	0.72474048471156	-1.08567622934576
H	6.78218212226447	0.26244763531658	-1.74286466980278
C	7.24573432334962	5.06733140335457	1.07563157507183
C	4.76125910316439	0.94922119674655	-1.52017678073268
H	4.46591268321282	0.66008948941440	-2.51998940479049
C	6.06227038926839	4.61622912416013	6.60716125993019
H	6.63055814495537	4.58653609290625	7.52494981108389
C	4.28130750157833	1.89980478323359	0.61362940409658
H	3.60934179543891	2.36023697803425	1.32419462796679
C	4.94042996862050	5.42777691244791	6.50249874900263
H	4.62583979088935	6.02962371826270	7.34457285351088
C	3.85269291291693	1.54403148141731	-0.65755442511327

H	2.83064158594702	1.73251662984511	-0.95443019983874
C	7.61701283558961	6.21429755136476	0.27494730176793
H	7.81949868236907	5.89555846949539	-0.74807866127655
H	8.51055767808411	6.67795864810116	0.69432456990146
H	6.80032264224033	6.93708417356743	0.27394573920448
C	2.41138903935607	-0.17074997117900	4.75549186423748
H	2.19739052303070	-0.51821573891175	3.74423835048918
H	1.48933393484572	-0.21428213260876	5.33711141326342
H	3.12596894305216	-0.84478485365203	5.22304127880382
C	1.82146057557632	2.21075755805159	4.24339658341049
H	2.17777380385179	3.23948394014900	4.20962143150189
H	0.97181281945166	2.15929070371568	4.92460497829187
H	1.48296479827491	1.92015340955714	3.24765823098905
C	3.46302526495352	1.68994123781437	6.08931836853056
H	4.30126174135809	1.06178952760946	6.38580469295732
H	2.67363462084123	1.58551724841740	6.83497749830123
H	3.78595117461968	2.72869949273017	6.07874446640247
C	5.43951148914240	-0.95970358827760	3.59577272315803
H	4.83096611213015	-0.93323940337909	2.70405877674505
C	6.79346385754238	-1.20417715983329	3.48361476849021
H	7.22417454694318	-1.29141319759630	2.49383609817527
C	7.69854187060694	-1.32207505633530	4.59208837335285
H	4.93643191494305	-1.19975185379879	4.51647405783318
C	9.00527360955340	-1.80160236892908	4.37382542990779
H	9.29571205081185	-2.09277500644110	3.37259025208375
C	7.33777479322894	-0.92979149987036	5.89788195591327
C	9.90781317666432	-1.90122647660027	5.41779489159375
H	6.35140960955817	-0.52684196972885	6.08292535903172
C	8.24794990581682	-1.02122580724737	6.93495453532778
C	9.53403149229988	-1.50945891480259	6.70217360771144
H	10.90746030989249	-2.27316200800471	5.23358838852954
H	7.96402416302631	-0.70055994913028	7.92896202342206
H	10.24423296001246	-1.57367913942025	7.51646636128935

Coordinates of IV (Intermediate between I and styrene) at Singlet State

Co	6.57189928883501	2.44100490978671	2.54503059374953
O	4.95882011139164	0.61186229639364	3.36512862430637
O	3.66067419801326	1.25924296282998	3.42939572325393
N	7.53550402865956	1.33856971397947	1.36232557203430
N	5.36061919371024	2.58789616522364	0.93737696898106
N	5.96870110960294	3.45411031134979	4.17037195384201
N	7.69068453510453	4.19175556831289	1.89161473576331
N	7.87045392455983	1.81836674500928	3.75423999039444
C	5.88481524403290	1.96909579675870	-0.15763318634290
C	6.73181774690026	3.18209794581024	5.26360611189615
C	9.75701954108191	0.45162075307827	4.14239214184185
H	10.51711887097466	-0.22667248824843	3.78254038938224
C	7.16044216340946	1.27520734774651	0.08418680461877
C	8.72754793085234	1.69908396937107	5.94458772001440
H	8.69269019091747	1.98709045185950	6.98505513669452
C	7.95009541844914	0.58267220698066	-0.82888284812941
H	7.66630016536589	0.51336964041939	-1.86837350989907
C	8.64776262221553	0.74751926277909	1.83435626429427
C	9.10953108468222	-0.02965701579401	-0.37163087390410
H	9.73252930503496	-0.57824874130095	-1.06367592119153
C	7.80712939537308	2.20286762875091	5.02971401932496
C	9.69865955984525	0.81636604388245	5.48611780485487
H	10.42204577335521	0.41155986165272	6.18070338343055
C	4.75627905177498	5.06790415541285	5.44235997089326
H	3.96839724776644	5.80704305475259	5.46574888574805
C	8.80923790233209	0.97880406922123	3.27967132528425
C	5.01388527846579	4.37691813642475	4.26629629567004
H	4.43750162588038	4.57550689294421	3.37586573250816
C	2.97965742072653	0.94183482932853	4.68337135653723
C	9.46842189835699	0.03870906469900	0.97265295460555
H	10.36255537935162	-0.44644667781088	1.33529580807651
C	5.22516383928793	1.97037209379082	-1.37597421214188
H	5.66076062010261	1.47239265272393	-2.22987882796368
C	8.29994285447703	5.08543306022771	1.50969640259386
C	3.99440190573795	2.60660506046981	-1.47839029759137
H	3.46307506088739	2.61484517137343	-2.42052982532735
C	6.52264521385647	3.82743922461755	6.47156512584582
H	7.14177942177690	3.59332955145770	7.32519117203795
C	4.17391648856641	3.18298603649980	0.83205988162976
H	3.77585797237709	3.63600907409784	1.72668176836789
C	5.52023799160145	4.78464588391595	6.56340197528595
H	5.34474708852341	5.30109905939285	7.49735354972698
C	3.45643306704967	3.21623865114998	-0.35621871184520

H	2.49603098342978	3.71069329071516	-0.38629560901418
C	9.06497355741867	6.21546690032242	1.02547211121189
H	9.04469758950463	6.22912368247090	-0.06483873381517
H	10.09723459158195	6.13183608047520	1.36687870958443
H	8.63173826562330	7.14070926378785	1.40680629794686
C	2.60855223364027	-0.53597419674805	4.75516654648828
H	2.04174169019988	-0.84086364849377	3.87457812175982
H	1.98987148793933	-0.70534206412177	5.63770525018638
H	3.49576645191003	-1.16309175070714	4.84712933228482
C	1.73575819135207	1.81824550502444	4.56707701840777
H	2.01416119744755	2.86994544451101	4.49058203445734
H	1.12089265394333	1.68432055379742	5.45764169350792
H	1.14470901466192	1.54154641262330	3.69292673079433
C	3.82816757445621	1.34610511796672	5.88109285124957
H	4.77957402368174	0.81629545313596	5.88029700668587
H	3.29718513500987	1.09314856113415	6.79979997127891
H	4.01287514345393	2.41723712137681	5.88161848498569
C	4.87400281092508	-0.48118104021971	2.42980107292809
C	6.11141545296277	-1.27603078748457	2.60684019598018
H	6.52594513611694	-1.30220646661342	3.60772832217191
C	6.74505772554021	-2.02590173605945	1.59956599573697
C	7.93625591716002	-2.73890564786748	1.90492219413242
H	8.32532460107229	-2.68918384515124	2.91471919828097
C	6.25849404908666	-2.10654674057359	0.26768849571636
C	8.60185061319123	-3.46520038356308	0.93904973686057
H	5.35502751758087	-1.57984252096126	-0.00834699878373
C	6.93324320488703	-2.84032035738320	-0.68941435989150
C	8.10932460928790	-3.51971063772543	-0.36781703617506
H	9.51438007913046	-3.98922921977239	1.19456894441927
H	6.54861743939832	-2.87940241521267	-1.70093767996846
H	8.63553510465096	-4.08589296695952	-1.12531510920087
H	4.75574453070053	-0.08924686258696	1.41787369737417
H	3.98350269178649	-1.07073048045822	2.66063758326522

Coordinates of IV (Intermediate between I and styrene) at Triplet State

Co	6.56848797006092	2.45278106732361	2.54192168875457
O	4.97555606514192	0.60473100220806	3.37671973675678
O	3.67933497253802	1.25753907717662	3.41826933399853
N	7.51872125411769	1.35100993019803	1.34702211810200
N	5.35113880939448	2.61851650286657	0.94066227584838
N	5.97726450270333	3.45566500996833	4.17718329763179
N	7.69686190866214	4.19884805301357	1.89404921341327
N	7.86554694874613	1.80940509360408	3.74188800554712
C	5.86751997795113	2.00642244633982	-0.16185722906867
C	6.73889838050058	3.16550286503845	5.26687321051261
C	9.73390108061945	0.41399741304283	4.11515380592710
H	10.48502341464082	-0.27034553109102	3.74806133666387
C	7.13752103624010	1.29857567137831	0.07000362870005
C	8.71826336954501	1.65273636694350	5.93136561152060
H	8.68518701045492	1.92778152279212	6.97537595315759
C	7.91576706212517	0.60257867708103	-0.84987361761022
H	7.62708461022554	0.54156119966807	-1.88857055944083
C	8.62561381866686	0.74468815171190	1.81151428638996
C	9.07048072696382	-0.02481228831333	-0.40031466799150
H	9.68481871506906	-0.57622283790066	-1.09786695154376
C	7.80491581981776	2.17870225137203	5.02186972325864
C	9.67922090314967	0.76411932227320	5.46297194404530
H	10.39685875470337	0.34207374911439	6.15325073482795
C	4.78108803358951	5.06774694213592	5.46658807439952
H	4.00021555131255	5.81398582513085	5.49813988010227
C	8.79306493072013	0.96263577592522	3.25835523854441
C	5.03130058359661	4.38645481791710	4.28326664794947
H	4.45617453395810	4.59966870556202	3.39537214778926
C	2.97801332668329	0.94522141189130	4.66218983186105
C	9.43585582390278	0.03233070478645	0.94252378151361
H	10.32603380467417	-0.46438693535760	1.29888804452270
C	5.20507614176668	2.02584023481870	-1.37847439069444
H	5.63473928229130	1.53390986011260	-2.23888119835272
C	8.28504666986393	5.09702611266662	1.49025966131087
C	3.97881500161993	2.67229719440303	-1.47111722709726
H	3.44547111734178	2.69446921597177	-2.41188857735197
C	6.53672984301923	3.80045628506970	6.48147719793093
H	7.15461875529558	3.55209219798955	7.33201577874030
C	4.16844659007693	3.22305204192599	0.84467679844250
H	3.77607189261689	3.66996482973467	1.74487054967439
C	5.54324728387787	4.76594327656879	6.58397318072452
H	5.37337717744216	5.27447232444660	7.52329204197865
C	3.44802689082886	3.27375051493003	-0.34122410138629

H	2.49110991791514	3.77532486080877	-0.36359121912045
C	9.02521625145214	6.23223731164151	0.98021139464082
H	9.01000462673520	6.21745337065250	-0.11016699360706
H	10.05750733369433	6.18214861790192	1.32804991749121
H	8.56822621669868	7.15635162840785	1.33568200726402
C	2.60513744389978	-0.53215242395216	4.73273796881339
H	2.05015290337392	-0.83875786569273	3.84524674487742
H	1.97479064693221	-0.69951914794994	5.60734129866905
H	3.49096513024975	-1.15920120656834	4.83757677055408
C	1.73692020593443	1.82203645209581	4.52264390029105
H	2.01726399273107	2.87335746199672	4.44771944996142
H	1.10676170638602	1.69131778103998	5.40297604822686
H	1.16045396545800	1.54319401587029	3.63947426262388
C	3.80813042321985	1.35333251289854	5.87155546723257
H	4.75918439119459	0.82317422324826	5.88682998219906
H	3.26303614720916	1.10382228627040	6.78290441310301
H	3.99356417941764	2.42444473678940	5.87115281495086
C	4.90284834856975	-0.48619721481972	2.43812770781187
C	6.13426154188932	-1.28650421770784	2.63895097887031
H	6.53933232550143	-1.30075170276342	3.64387074664354
C	6.77732897198451	-2.04538094363105	1.64499363255346
C	7.96827133666700	-2.75219144571586	1.96588656437851
H	8.34930877796468	-2.69295411076314	2.97825919964773
C	6.30189101622137	-2.13828675358216	0.30994627444226
C	8.64412032571188	-3.48375981864269	1.01122236987106
H	5.39901157565523	-1.61663493157822	0.02248664114007
C	6.98681086564162	-2.87761218287707	-0.63573159322076
C	8.16230317782233	-3.55064075893023	-0.29923881579660
H	9.55626379136589	-4.00267386604641	1.27835686907720
H	6.61047330486012	-2.92607542095679	-1.64997521026131
H	8.69652741987278	-4.12112702546624	-1.04783504009867
H	4.80871740760394	-0.09088113301769	1.42474215219149
H	4.00411398364822	-1.07135914139953	2.64758705457491

Coordinates of IV (Intermediate between I and styrene) at broken symmetry BS(3,1) triplet state

Co	6.36494786388618	2.47805953943507	2.50982487180630
O	4.95677581172433	0.79400566680938	3.33967723506701
O	3.66052306154648	1.45428941639931	3.34139997979918
N	7.51800746823324	1.33629085072625	1.18147729956563
N	5.21614875471681	2.43325094190997	0.72017148197778
N	6.11833457052858	3.39813984697821	4.40173041208180
N	7.30377241315523	4.23815820674094	1.80310064445628
N	7.87781327550938	1.63059941533282	3.66776403165518
C	5.85543367265656	1.95017252231168	-0.36774378171252
C	6.89771500632199	2.91223176601845	5.38932712641924
C	9.76115932794829	0.21162684208983	3.85537039913477
H	10.49542443814090	-0.43581181124526	3.39965648588263
C	7.15842956510675	1.30350184786530	-0.09891142523552
C	8.82997373479982	1.28979687364016	5.80107526777573
H	8.84653399926792	1.48313599909991	6.86305930983101
C	7.96401565658876	0.66431789153498	-1.03665675197046
H	7.68891009903052	0.62522974321351	-2.07982779400462
C	8.61574888984846	0.72541550469249	1.62854243358801
C	9.12808285776750	0.04993327002412	-0.59587058272867
H	9.76673822269426	-0.45983664945878	-1.30386124285555
C	7.89167688466765	1.89791476961139	4.97149310934645
C	9.76031136190960	0.43305559658800	5.22749781224829
H	10.49813090700468	-0.05324207972243	5.85122880088839
C	5.11471325567144	4.92400549494316	5.93065493694795
H	4.40429235509467	5.72049272509156	6.09958248877334
C	8.78657179927055	0.83998346054091	3.09190792452928
C	5.25639599519760	4.37942074993532	4.66314640758391
H	4.66058998821309	4.73416784759050	3.83205478404583
C	2.88550990400477	1.15744846988558	4.55232710258653
C	9.46287399093551	0.06189358608165	0.75203959226087
H	10.35185126073411	-0.43794444954162	1.10546679065050
C	5.28940464055185	2.03970864434053	-1.63161022488771
H	5.80720540602919	1.64775135592131	-2.49419568989190
C	7.78540139222578	5.19518193584950	1.39541593104313
C	4.04550202044918	2.63947102341531	-1.77400473721442
H	3.59055684033042	2.71779197730995	-2.75233122419026
C	6.79888513017503	3.39800670029408	6.68602930046179
H	7.42453539986698	2.99973364980382	7.47074960597196
C	4.02013542699585	3.00442289943503	0.58123523056449
H	3.55031692335512	3.36169929487031	1.48741088265725
C	5.89576509105186	4.41629600550476	6.95816111096368
H	5.80929711769719	4.80966879986304	7.96207204284754

C	3.39637410632121	3.13034866928292	-0.65128711111483
H	2.42511135044113	3.59933259894337	-0.71841025284953
C	8.39118179978860	6.40340499533905	0.88060181842219
H	8.21449872942658	6.47022964597444	-0.19338784969937
H	9.46472695292648	6.38173423850405	1.07116687879179
H	7.95236484621051	7.27027028716005	1.37577403546835
C	2.33435150634031	-0.26563431324516	4.52638839291748
H	1.83957387631021	-0.47558740873627	3.57736597459796
H	1.60154408435170	-0.37544364667759	5.32730178343488
H	3.11841290670381	-1.00371509933247	4.69661093935195
C	1.76063377801951	2.17993956664246	4.41219284524768
H	2.16132743331027	3.19380257354277	4.41541901287093
H	1.07384079366066	2.07224220492050	5.25241427261591
H	1.20329652972888	2.01895487531485	3.48832778390403
C	3.70383451672735	1.39691039175313	5.81173695305307
H	4.59219315879743	0.76759105038106	5.82651353601927
H	3.09431651849580	1.14718384624276	6.68114083056833
H	4.00030407662820	2.43852450437528	5.89529851840785
C	4.89409547493759	-0.39769247067693	2.51278115855499
C	6.09881884994368	-1.19001033123516	2.84443486898074
H	6.39667671371929	-1.19056265403475	3.88605063962604
C	6.84468883238302	-1.96250727603076	1.93601138263386
C	7.99402600711632	-2.65908876390406	2.39716751474227
H	8.26333259103802	-2.58003630542892	3.44311091805646
C	6.51515757565706	-2.08269375384621	0.56101141687403
C	8.76739825491012	-3.40906618073687	1.53592124396169
H	5.65045252967340	-1.56644554663163	0.16743570023560
C	7.29508705732621	-2.84151652589050	-0.28978411944778
C	8.42632253637966	-3.50632362512559	0.18421329809724
H	9.64536946071295	-3.92105432042694	1.90944104286232
H	7.03005859436588	-2.90975644151162	-1.33735417022223
H	9.03638255325789	-4.09214745323506	-0.49089119851197
H	4.83522733442742	-0.10742669880522	1.46458735120841
H	3.98001688905534	-0.93487677459577	2.76502721161889

Coordinates of IV (Intermediate between I and styrene) at quintet state

Co	6.35069202067946	2.52640536580446	2.48553186082794
O	4.94316570528271	0.81706316763574	3.30914444286638
O	3.62993530747536	1.44014180316930	3.35380910427324
N	7.49295696856537	1.36121069999089	1.16632459027924
N	5.19900858363828	2.47774659435342	0.70212552487757
N	6.07765183827829	3.43487205903028	4.37666331286425
N	7.34101165152738	4.26216447610132	1.79275348181971
N	7.85175232767019	1.66935843723992	3.65279031049399
C	5.83285447264435	1.98210768810906	-0.38498863391310
C	6.86350535629826	2.96017346763351	5.36625242497643
C	9.71915142982905	0.23117671482030	3.85564294908401
H	10.44938405149927	-0.42570922200480	3.40658280450727
C	7.13179739987925	1.32574660300635	-0.11442926154258
C	8.78463865031664	1.32402556933198	5.79379187112516
H	8.79436618210669	1.51700793382803	6.85607785444049
C	7.93339093100223	0.67468748371060	-1.04862692601576
H	7.65798989539536	0.63116517947849	-2.09172039925127
C	8.58703515137970	0.74548480078201	1.61877784098664
C	9.09385855824445	0.05423079771803	-0.60403161883163
H	9.72845792648080	-0.46467161317879	-1.30904860610567
C	7.85757717778306	1.94064952729450	4.95674195616370
C	9.71123840024466	0.45675168224102	5.22801956494508
H	10.43923489897304	-0.03636848125657	5.85801450373833
C	5.06782434627592	4.96681881182315	5.89604523967532
H	4.35234620634860	5.75965783310485	6.06123141096609
C	8.75527524137870	0.86648054287045	3.08330408141414
C	5.20814957341090	4.41209506507536	4.63244427394232
H	4.60648014957444	4.75456172717258	3.80004114660724
C	2.90869400031304	1.09580775281264	4.58458843458420
C	9.42970523369017	0.07157660980367	0.74464674311878
H	10.31586034023752	-0.43212857527768	1.10043205904412
C	5.26210061828942	2.06760560079898	-1.64745419637452
H	5.77423166025860	1.66748865588479	-2.50982313888233
C	7.89660906091407	5.19114449429409	1.41552346682169
C	4.02035277462600	2.67368639591079	-1.78890897662599
H	3.56242709338163	2.74882254379665	-2.76614167679860
C	6.76583448791783	3.45832177770341	6.65897111873081
H	7.39671716924133	3.07136547679655	7.44542723661058
C	4.00394953420103	3.05274894584597	0.56494182325279
H	3.53886205860812	3.41817060142643	1.47070389999446
C	5.85764164302832	4.47453724424502	6.92495548548583
H	5.77341671650690	4.87771480896343	7.92523157490399
C	3.37645812980921	3.17343854230913	-0.66655693207746

H	2.40677720378425	3.64586858895664	-0.73331384260749
C	8.60156488093165	6.36153487824365	0.94116862430543
H	8.60614577087316	6.36723720645285	-0.14938279230202
H	9.62830299847597	6.33989379477749	1.30841849046535
H	8.10438185886645	7.26027368042467	1.30774436666141
C	2.46665947528480	-0.36462513745356	4.58325500569143
H	1.93422324371759	-0.61369409922898	3.66453609204848
H	1.79368126570725	-0.53021972629164	5.42586497056936
H	3.31565484975435	-1.03781491830292	4.70347704671653
C	1.70865391848597	2.03216114913706	4.47396694168885
H	2.03350628846324	3.07297971969548	4.46089556644525
H	1.05895750922193	1.87938437110218	5.33632361894124
H	1.13600536961331	1.82516146966654	3.56887105098540
C	3.74089700007585	1.40257897336654	5.82032008739311
H	4.66776864788761	0.83149390769015	5.81762697141048
H	3.17151221049398	1.12424572331548	6.70805716940054
H	3.97042509000567	2.46250026035346	5.88493647932879
C	4.89633167017089	-0.35502251675108	2.45510825680313
C	6.07774198502992	-1.17100229772335	2.81571161630806
H	6.35964661322668	-1.16576129716010	3.86175672452283
C	6.82363097596240	-1.96259137966479	1.92405803327726
C	7.95892536039323	-2.67067472571994	2.40400793674607
H	8.21666606203735	-2.58729108290716	3.45267875146928
C	6.50862480722234	-2.08909248619744	0.54539051617590
C	8.73312382843423	-3.43647039225152	1.55715549059559
H	5.65456487148782	-1.56544382818897	0.13805159647390
C	7.28915902829072	-2.86440930857195	-0.29053798309771
C	8.40676663239952	-3.53952140848404	0.20167798394136
H	9.59999933504044	-3.95693239882457	1.94482781051710
H	7.03516320021277	-2.93810941934076	-1.34054234105078
H	9.01709200226217	-4.13844073568911	-0.46158791120377
H	4.88636740534007	-0.04298526611813	1.41129753875342
H	3.96289771763770	-0.88222688851219	2.65234810562197

Coordinates of V (dioxygen adduct intermediate) at Singlet state

Co	7.01571588122441	1.84321554249003	2.55128964642452
O	3.33891077027327	-1.18553225053808	4.44593269190267
O	1.89924223249013	-0.99782842994797	4.46538451657856
N	8.14564665802005	0.92128442883043	1.36259593561943
N	5.77264187832810	1.74210869716706	0.96699215284998
N	6.20084071034549	2.63031826765044	4.21004268627923
N	7.76144932650987	3.75137466380796	1.95566817519901
N	8.40006459604222	1.43434357641475	3.75699093332876
C	6.39503500322725	1.24901351329956	-0.13983623290915
C	6.98372649791871	2.44545620121563	5.30935046938730
C	10.50095075234202	0.41996156255119	4.12875447504632
H	11.36970608452349	-0.10543667704380	3.75908651430901
C	7.77301808470879	0.78697093487024	0.08934947527895
C	9.25242657505556	1.40956038404985	5.95148681219222
H	9.15788190456643	1.64946554552019	7.00009395063072
C	8.65755002165012	0.23075522023348	-0.82894480339586
H	8.38098367340494	0.11349990025135	-1.86581237402980
C	9.34088588547683	0.51857868675404	1.82936295270632
C	9.90434454666313	-0.18450317169052	-0.37791002791979
H	10.60420144764515	-0.62471135464685	-1.07483582643622
C	8.25303447801314	1.74862193695073	5.04445519529337
C	10.37803712545089	0.74600081728870	5.47801519031241
H	11.16569610710722	0.47294174225794	6.16699267617716
C	4.60868493059303	3.78263081604366	5.56472820705087
H	3.66597362015352	4.30789877643518	5.61622558783203
C	9.47167312269500	0.77923565178901	3.27350835674232
C	5.05841029318940	3.30181884128434	4.34308000576091
H	4.48363250277603	3.46708440765386	3.44509670552672
C	1.58339476700986	0.34151286368810	4.94684007719925
C	10.25827747574957	-0.05488681624117	0.96275274076308
H	11.22253772213028	-0.38756352510303	1.31900437184630
C	5.74228732581936	1.15314182697274	-1.35704026006867
H	6.26059048827808	0.76256301340587	-2.22019932339541
C	8.18926839162684	4.77124744857722	1.65272003416123
C	4.41088385303424	1.53874124560174	-1.44414400582489
H	3.88173702308498	1.46141932137116	-2.38425345115721
C	6.59674995465917	2.90122976517363	6.55839062150399
H	7.23161517710336	2.73923899257308	7.41726697438418
C	4.49320740383627	2.10151307030945	0.87417983740797
H	4.02525835977638	2.47161154657330	1.77279488775063
C	5.38568897179154	3.56964922332562	6.69109608527758
H	5.06377375858666	3.92452521626047	7.66078773538367
C	3.77199176936759	2.00796366880736	-0.30792104386229

H	2.73307952861839	2.30485858604077	-0.32286172000491
C	8.72896303261217	6.05909664063150	1.27151228746008
H	8.98556932028431	6.04845804469815	0.21167077798945
H	9.62408086045360	6.26730609785871	1.85880821083127
H	7.98633402789773	6.83591720293191	1.45670148296374
C	2.18341224460472	0.56037554457829	6.33158695273152
H	1.86726608019478	-0.22993368324728	7.01425667223895
H	1.84531138769055	1.51865625815989	6.72865982252365
H	3.27166728910003	0.57579854740794	6.28666031070414
C	0.05954840235174	0.29969830290837	5.00060632775681
H	-0.35592333709691	0.08464653728571	4.01504221405123
H	-0.31393429957866	1.27052608121402	5.32832650985044
H	-0.28302152319081	-0.45983359020587	5.70444906973687
C	2.07528375743971	1.38649283955548	3.95381778139973
H	3.15901691299084	1.34330365871058	3.86342417347007
H	1.80633474338857	2.38504150853478	4.30073660584540
H	1.63096203110728	1.22671838752875	2.97042082744372
C	3.72027055270722	-1.37113803307368	3.09059349237445
C	5.20613665323093	-1.65681562342845	3.10026460716786
H	5.43843380146364	-2.42639081086000	3.83451125809443
C	5.81673647269564	-1.98359407227955	1.76732567288352
C	7.12183345125444	-2.48431589094185	1.74775600081784
H	7.66207543286795	-2.60309451965100	2.67931186947511
C	5.13118920695025	-1.82155779472570	0.56593727860766
C	7.72462031703490	-2.82927014935865	0.54802859134027
H	4.12790281285983	-1.41901938084973	0.55267668267277
C	5.73291822605227	-2.17785902746124	-0.63678834806935
C	7.02634144926328	-2.68304564200617	-0.64822356815601
H	8.73796796383620	-3.20862059847457	0.54344232138552
H	5.19092830072351	-2.04884173326343	-1.56438535027242
H	7.49522334924304	-2.95310081728493	-1.58570263556381
H	3.49317016149116	-0.48209716310853	2.50036939521139
H	3.19993439557171	-2.23247136910065	2.66290054638690
O	6.21546516829574	-0.54163635398215	4.88889741165962
O	5.90186467333950	-0.44086307697984	3.61981713788177

Coordinates of V (dioxygen adduct intermediate) at Triplet state

Co	7.00494436740999	1.84306217944755	2.55188318335818
O	3.36477498982124	-1.15787766281240	4.44808534591562
O	1.92251282495020	-0.98995192843559	4.47031889659180
N	8.13637724780360	0.91680998762304	1.36771030912265
N	5.76538550005047	1.74093820337902	0.96485143233111
N	6.18851455392431	2.63667674391417	4.20678240351721
N	7.75803113805143	3.74947395080045	1.95286870724213
N	8.38922860033565	1.44055073784564	3.76011405595733
C	6.38848599438995	1.24136204462089	-0.13866714329995
C	6.97089161948707	2.45661889598358	5.30737894323636
C	10.48907650731660	0.42639914143242	4.13811914032948
H	11.35773394347059	-0.10124636763906	3.77144451626608
C	7.76500873978713	0.77719615579024	0.09463164662924
C	9.23933854671071	1.42406133521570	5.95556756801269
H	9.14365996316363	1.66805782893215	7.00312811743116
C	8.64978645895817	0.21571547025967	-0.82018963611772
H	8.37420727519656	0.09409074531427	-1.85680657544754
C	9.33090655916399	0.51543520564008	1.83739326874629
C	9.89569575836857	-0.19881719865587	-0.36596852418184
H	10.59566048760305	-0.64324254914921	-1.06009991099726
C	8.24087161116181	1.75956920918298	5.04618368208310
C	10.36516840546392	0.75820180962783	5.48590076339661
H	11.15204289382459	0.48758368602206	6.17674231936770
C	4.59569195450631	3.79487152442505	5.55582900884605
H	3.65302327357425	4.32049018755626	5.60450664960834
C	9.46079582200383	0.78272232453631	3.28041746328416
C	5.04617282413046	3.30905511249325	4.33646826693147
H	4.47200707839555	3.47092295890516	3.43750468310638
C	1.58807476340106	0.34647913473571	4.94676292039639
C	10.24856769430198	-0.06299262093904	0.97433083599443
H	11.21223791903675	-0.39478971799816	1.33300437732517
C	5.73806992803707	1.14274992931081	-1.35691910845952
H	6.25686807002846	0.74655821764204	-2.21720214737126
C	8.20521383916244	4.76281415895081	1.65590973585265
C	4.40843847386924	1.53315388653836	-1.44879876077119
H	3.88110888910873	1.45373448080795	-2.38975382741581
C	6.58297936173700	2.91734242827604	6.55435303110509
H	7.21735419661631	2.75896475798607	7.41427284996952
C	4.48782584072232	2.10589068004655	0.86716026049493
H	4.01927351981028	2.48236666060512	1.76281739639874
C	5.37180392503089	3.58619619134902	6.68362180152852
H	5.04915970418655	3.94479961229298	7.65170174888842
C	3.76904124612301	2.01050207063111	-0.31623596211889

H	2.73154995263414	2.31212442619448	-0.33494376635401
C	8.76943796604582	6.04223775114270	1.28171800981961
H	9.02956053538297	6.03130855511937	0.22274143370855
H	9.66609793923656	6.23150254456540	1.87304986302228
H	8.04048056256591	6.83171437898075	1.46767046697983
C	2.18986004183847	0.58108038336351	6.32810675518820
H	1.88913882739468	-0.21140889249570	7.01520694156611
H	1.83778086789901	1.53535582673254	6.72268154260284
H	3.27753849760059	0.61403794252237	6.27907262233381
C	0.06526995667375	0.28123185381779	5.00680005386729
H	-0.35059173937335	0.05398694094811	4.02413454794151
H	-0.32208620631621	1.24801675987735	5.33037859251547
H	-0.26266241309757	-0.47945832809914	5.71633178501990
C	2.05998353668839	1.39348624402735	3.94630657821661
H	3.14372587373665	1.36521194890412	3.85166111003978
H	1.77815502415600	2.38998296371858	4.28885226176336
H	1.61424755238137	1.22154133959387	2.96562613916258
C	3.74338523122159	-1.34784765027590	3.09270250015034
C	5.22543664763502	-1.65288641786475	3.09824434087273
H	5.44878920336719	-2.42572430841592	3.83189598030396
C	5.82694375459445	-1.98857490369983	1.76330992169610
C	7.12897090358697	-2.49703277065735	1.73865489607261
H	7.67359176182816	-2.61558854714010	2.66769159256247
C	5.13575976441637	-1.82749121292454	0.56503953806281
C	7.72323291922134	-2.84994328036298	0.53698816744067
H	4.13469157083210	-1.41938760890147	0.55553650701610
C	5.72891400000719	-2.19178749151635	-0.63954766319237
C	7.01943354847828	-2.70416793786337	-0.65608284677477
H	8.73439902689289	-3.23501965666130	0.52842570102730
H	5.18251667251789	-2.06325065531672	-1.56463605692384
H	7.48179468729719	-2.98028043096936	-1.59504291179955
H	3.52512205455935	-0.45702210812585	2.50167420338923
H	3.21285268210789	-2.20373621876793	2.66678570690401
O	6.22415073212311	-0.53627726609372	4.89195715129458
O	5.94510575366502	-0.44900377584767	3.61385460141957

Coordinates of V (dioxygen adduct intermediate) at broken symmetry BS(3,1) triplet state

Co	6.87288522691341	1.98612499626748	2.59812571556116
O	3.22167334162895	-1.02656485519803	4.34862458837659
O	1.78632209866249	-0.81984776883538	4.27986461095234
N	8.14340898070047	0.92278235223584	1.32789236815994
N	5.69210481214047	1.62097263347907	0.90389017836886
N	6.29372304343323	2.61767367444831	4.51168320683296
N	7.55883915677195	3.82523723085415	1.94737826793733
N	8.47162272889096	1.38932223165134	3.78339581156535
C	6.35574916508415	1.14794614036653	-0.17556430956895
C	7.16920882404933	2.34077188389767	5.50317584516811
C	10.61914908281401	0.42288570388392	3.98685692651084
H	11.47218933324262	-0.06923930361247	3.54318134351899
C	7.75951053186380	0.75732386535840	0.06750261198475
C	9.48425314597264	1.33282477859237	5.91296176607237
H	9.46221502120714	1.55080390632922	6.96995865512251
C	8.64254543744049	0.22573453660823	-0.86785895964789
H	8.35150305268551	0.08431460682157	-1.89741670417178
C	9.34617522619792	0.55416516816839	1.76845312824931
C	9.91210074042335	-0.13685640437790	-0.43936769759618
H	10.61679626333487	-0.55390376433177	-1.14582506556737
C	8.41873074732880	1.66946144166201	5.08250726363569
C	10.58672343392386	0.70513621483134	5.34816219009539
H	11.42634039428253	0.43215248719557	5.97302641267120
C	4.79357358831916	3.57640006778508	6.09683060692074
H	3.84546574649876	4.05892126373955	6.28528872179717
C	9.52114918614367	0.78578068827988	3.21952838256725
C	5.14530622893278	3.22826495925781	4.80172745848939
H	4.48880575530479	3.44398772720555	3.97039299981798
C	1.46002979622955	0.51930813122245	4.75424219089875
C	10.27708054799760	0.00934617202706	0.89425273510700
H	11.25683134998017	-0.29070473771695	1.23623780303383
C	5.72482083767159	1.00553840464931	-1.40176926111515
H	6.26808914783019	0.62637516104967	-2.25406233801155
C	7.93216089764423	4.84303666527812	1.57554941682297
C	4.38205550205101	1.33661122672613	-1.51446468989469
H	3.87259809427270	1.22216142548508	-2.46173931605519
C	6.88771933379982	2.67029517447726	6.82051406596472
H	7.59374698754859	2.44252064201894	7.60508805393482
C	4.40276310744573	1.94204193274531	0.79033045208913
H	3.91645858444933	2.31768108584657	1.67976465803888
C	5.68160925187514	3.28880818813121	7.12137271021397
H	5.44393067768900	3.54372777668454	8.14542166546649

C	3.70487788210461	1.80655688301530	-0.39985471252170
H	2.65769332973826	2.06967248147092	-0.44100830432542
C	8.40494900754941	6.12576837986289	1.10726456642512
H	8.67854540036313	6.05082917956043	0.05430091067318
H	9.27628053715906	6.42598146324887	1.69038361267743
H	7.61514878531022	6.86843210895045	1.22631514271941
C	1.95404605070876	0.70520157230470	6.18472504323529
H	1.56019527315251	-0.08165309744885	6.82997163731076
H	1.61569635322033	1.66956585736389	6.56677918729378
H	3.04245840175207	0.68656461489928	6.22648024396492
C	-0.06374030573722	0.50798095503641	4.68857026036219
H	-0.40497575914443	0.32347309723025	3.66891050423136
H	-0.44308004902961	1.47884363271047	5.00940786410122
H	-0.47450141816661	-0.25960429583894	5.34566354519200
C	2.04811878395509	1.56730184959819	3.81844803111349
H	3.13471962176040	1.50265592990800	3.81130511556370
H	1.77223453198402	2.56627284324295	4.15883131934002
H	1.67735361208088	1.43064536171681	2.80161842346289
C	3.67457838196059	-1.25012827828538	3.02153137294935
C	5.14887680478535	-1.56749234328893	3.12668736729435
H	5.31597687004437	-2.31854885708744	3.89701243911366
C	5.83516051110726	-1.94976065573295	1.84657917265664
C	7.16136606391342	-2.38410760836276	1.91950604466154
H	7.66245419031195	-2.41051765985164	2.87932260207986
C	5.19961325610660	-1.90591168047352	0.60914028951337
C	7.83574864432321	-2.77831121401191	0.77537259986113
H	4.18032624121082	-1.55599632598674	0.52287953076283
C	5.87375473299567	-2.31165137859782	-0.53772421438550
C	7.18905093349876	-2.74845411302379	-0.45738635028535
H	8.86489354840072	-3.10536890889254	0.84199244870980
H	5.37079377884638	-2.27200330999016	-1.49491454550465
H	7.71463440495360	-3.05502318474063	-1.35248876920465
H	3.50066551135750	-0.37202500392836	2.39842190909310
H	3.15975683146996	-2.11025149545241	2.58535619671978
O	6.16328470425559	-0.47584245699989	4.92484442118488
O	5.83967014101432	-0.35298805331439	3.66061062364134

Coordinates of V (dioxygen adduct intermediate) at quintet state

Co	6.85556078798758	1.97667901002863	2.57789443780897
O	3.22861182304176	-1.05922942780274	4.37509767602774
O	1.79332114229124	-0.84501190461186	4.32044679891531
N	8.15126197120244	0.93131807987270	1.31406571478290
N	5.69235986749872	1.58541082423852	0.88086261040803
N	6.26278909553420	2.64008165651737	4.48000515796178
N	7.55012524695958	3.81161266315906	1.91496851865853
N	8.44243132309648	1.38637975857634	3.77939509420481
C	6.37457640160539	1.14983472272220	-0.20412408758459
C	7.12346502661205	2.35300437406515	5.48276343616253
C	10.59459207174949	0.43475203640218	4.00722973183885
H	11.45641958282723	-0.05176751807245	3.57424433865583
C	7.78105923202110	0.77041431705688	0.04787599309688
C	9.43539094136435	1.34687770198985	5.91920635362786
H	9.40356582043110	1.57178290185849	6.97463758121966
C	8.67774233359345	0.24446497193876	-0.87891042378787
H	8.39995223659416	0.10533483899456	-1.91273801062785
C	9.34840065662195	0.56102905883860	1.77067672465495
C	9.94248927934263	-0.12009248006936	-0.43507403773716
H	10.65619785218001	-0.53424559083623	-1.13426459559281
C	8.37559997719677	1.67466720639322	5.07735952914452
C	10.54643242912417	0.72080678440371	5.36790170691247
H	11.38136157061350	0.45531983442120	6.00231944806151
C	4.75546935066407	3.62083717487384	6.04510482254290
H	3.81213566692764	4.11729140466337	6.22159855397510
C	9.50286116504761	0.78921791903606	3.22591989547649
C	5.11881605104496	3.26763926770031	4.75425673986350
H	4.47676521860604	3.49212967001175	3.91347123570664
C	1.47854291959098	0.49325279136861	4.80496412360784
C	10.29052749066502	0.01969459714199	0.90466557939831
H	11.26581714861355	-0.28370611988447	1.25693184050779
C	5.75515859812414	1.02852045507986	-1.43894676263366
H	6.31189344017287	0.68135718255342	-2.29647711884056
C	7.96796901343002	4.81968941403275	1.56484767749611
C	4.40636871043420	1.33727193621252	-1.55352572120905
H	3.90654733482728	1.23946700136059	-2.50783410808272
C	6.82766381256241	2.68490250423420	6.79688848450387
H	7.52042929593953	2.44789004181222	7.59073028856664
C	4.39622335210961	1.88037242033748	0.76788192653524
H	3.89779602602390	2.21920223736071	1.66580326904529
C	5.62511046812715	3.31893663556138	7.08184888033412
H	5.37629502052254	3.57569777155866	8.10283114252288
C	3.71008140301031	1.76061626215546	-0.43120303889554

H	2.65725062572698	2.00030926603910	-0.47400091986499
C	8.49842763555929	6.08963726401550	1.12446733420178
H	8.71637928140484	6.04320664079131	0.05689968715258
H	9.41439817526561	6.30950740129929	1.67456500340761
H	7.76509521095598	6.87466206501482	1.31311342777011
C	1.98593976321654	0.66887888012825	6.23217410538235
H	1.59166993350556	-0.11811615470572	6.87697748633160
H	1.65786580151863	1.63395965423077	6.62120463950331
H	3.07448243432116	0.64206278656162	6.26453914790694
C	-0.04590733433151	0.49082610982965	4.75272863808898
H	-0.39702869785752	0.31246896596741	3.73534738440846
H	-0.41694375019542	1.46235358949982	5.08116200315778
H	-0.45500305143016	-0.27736228626925	5.41016108141997
C	2.06340073607397	1.54377116757565	3.86992776581595
H	3.14966115397756	1.47554569226193	3.85280457440395
H	1.79426998051842	2.54179451050221	4.21845752596315
H	1.68306715877543	1.41425770061229	2.85564897567324
C	3.66925657633299	-1.27053186880877	3.04193589883996
C	5.14970308379491	-1.56733347369002	3.12620851084798
H	5.33846771229221	-2.31763063709586	3.89226932714056
C	5.82392799117342	-1.93761253949508	1.83596449659912
C	7.14884331112926	-2.37984429026117	1.89331080922788
H	7.65617021725420	-2.42227420750264	2.84941209099212
C	5.17957596971120	-1.87323871597761	0.60322035609638
C	7.81335915543541	-2.76098650203597	0.73834284377617
H	4.16169572433224	-1.51605626185990	0.52970372298780
C	5.84349148430404	-2.26677185139756	-0.55427214273076
C	7.15765005270012	-2.71108187142646	-0.48941112208834
H	8.84150828435278	-3.09365467436664	0.79277828426363
H	5.33395118032024	-2.21139658499969	-1.50720799373898
H	7.67507187858034	-3.00820526856290	-1.39249701736593
H	3.47546928951183	-0.39158505445449	2.42574514922161
H	3.16165488122262	-2.13545845198271	2.60636356589608
O	6.12786130045630	-0.44900240161141	4.92677552599781
O	5.83372869415155	-0.34537298508198	3.65355242607952

Coordinates of VI (Final product) at singlet state

Co	7.00507272787189	1.52854156323230	2.54791561460294
O	2.93861176356382	-1.47861554987267	4.25355056203913
O	1.50710361501060	-1.34263855568694	4.44527524290457
N	8.28323583482881	0.71916921136923	1.43688630740124
N	5.92401895067843	1.46288793637682	0.86013422313573
N	6.04691882482038	2.34759602186893	4.11512700369846
N	7.62004121895582	3.30252423909884	2.02772034222156
N	8.37546739006701	1.35101456368136	3.82024061129793
C	6.63942367171448	0.97910908492444	-0.19012229880289
C	6.80341617241797	2.31835865337204	5.24508938123713
C	10.54476398764535	0.58103313562072	4.32318898470355
H	11.47163041037214	0.11622021899171	4.01961720009681
C	7.98687223983277	0.51312330904810	0.15597104132899
C	9.15000328117997	1.59542419706042	6.02305834254972
H	8.99999323236482	1.91742079915883	7.04273170436263
C	8.92949265473797	-0.09081323874355	-0.67121852412990
H	8.71314221206822	-0.28030769921203	-1.71168687322304
C	9.46094240761980	0.39200956698812	1.99417414072991
C	10.15292359375280	-0.45413365597325	-0.12418590913053
H	10.89694924871189	-0.92880442100605	-0.74901274916695
C	8.14865941205794	1.75302426119764	5.06862585632793
C	10.34896787724252	1.01040979769981	5.63403525728866
H	11.13921042370478	0.88051528283613	6.36075974521705
C	4.26062862127129	3.32341859100926	5.35119741537625
H	3.24992161477754	3.70461593209818	5.34468514527931
C	9.51410331206212	0.76448348745438	3.41544920002045
C	4.81631311486382	2.84540648378771	4.17229332523770
H	4.25107345190761	2.86600759163690	3.25582137416258
C	1.20745777507384	0.00503494579548	4.91383853744378
C	10.43584666804515	-0.21446137842927	1.21898152076015
H	11.38917036723410	-0.48963579404934	1.64612206955543
C	6.11120807444316	0.94354950879875	-1.46895168378913
H	6.70061676746684	0.56006392898229	-2.28797973354881
C	7.96952280396314	4.34643221788354	1.71866772505378
C	4.81622114136336	1.39790057572079	-1.67812750485335
H	4.38634372178989	1.37845522703219	-2.67017580503355
C	6.31265029190634	2.78565663122096	6.45289957023807
H	6.92918017605614	2.75259483684369	7.33919008936024
C	4.67384383271414	1.86655778455643	0.65922061567137
H	4.12056710179108	2.20803050620238	1.51772247100229
C	5.01974581677290	3.29115185148963	6.50842392698678
H	4.61744087149795	3.65379385700730	7.44451060968377
C	4.08159296155033	1.85171141294106	-0.59635776646626

H	3.06273735463071	2.19447203857601	-0.70553394192895
C	8.40620973275977	5.66611823147972	1.32872675827419
H	8.68504269434609	5.65806820102524	0.27429141111517
H	9.26709009048212	5.95585077914586	1.93254457652835
H	7.59394415343630	6.37675498386823	1.48674316990185
C	1.97236043893696	0.29836402971856	6.20050297490936
H	1.77202825395292	-0.47082408486915	6.94817120408460
H	1.65641654135182	1.26282979901890	6.60098307750337
H	3.04454205564048	0.34184851311261	6.01431268038883
C	-0.29382456627167	-0.08132930889982	5.17008080871232
H	-0.82786856996701	-0.34256167153940	4.25523111170665
H	-0.65565125265259	0.88741718084798	5.51659147499370
H	-0.51337025575262	-0.82755381901830	5.93479978336110
C	1.52534410707854	1.03443483952127	3.83338245725060
H	2.58838955315459	1.03349480170744	3.59807109736578
H	1.25905909762879	2.03148129389257	4.18789977672023
H	0.95964431470921	0.82865309654637	2.92340029856939
C	3.17887844879200	-1.67306693188036	2.86369810467329
C	4.68303213054892	-1.56506061369839	2.66789898896537
H	5.18150453159176	-2.20761991810923	3.40018708982072
C	5.14333270154754	-1.95302428389770	1.28150159285228
C	6.39560330833019	-2.54756977982956	1.12366511662141
H	7.00402316465202	-2.73806037114534	1.99830900175615
C	4.36737977181707	-1.70970408041324	0.14828446750998
C	6.86946087112300	-2.88501649087369	-0.13848273676028
H	3.39932243405082	-1.23564750946340	0.24021512315346
C	4.83261625774933	-2.05895060193556	-1.11404128911741
C	6.08605329492156	-2.64341899300404	-1.26214102090496
H	7.84970103550709	-3.33215127809513	-0.24462843537251
H	4.22100656158240	-1.85953715135915	-1.98473815600839
H	6.45164389173925	-2.90387529109369	-2.24740757703061
H	2.65723886425666	-0.91109600228322	2.28538399807307
H	2.83676155840170	-2.66245881726156	2.54774653970408
O	6.43119885997708	-0.13439781372275	3.14133312630869
O	5.00035893217534	-0.20570989608143	2.99713103746732

Coordinates of VI (Final product) at triplet state

Co	7.02248742496220	1.74378490023224	2.57036804848186
O	2.84051353825404	-1.50275087314565	4.22369398132249
O	1.41012159337978	-1.27298740559084	4.31038214104272
N	8.23725884646298	0.96516264284110	1.35362727038323
N	5.81658657998503	1.65947050791054	0.94124848192434
N	6.14141965093544	2.40248624990196	4.26743065333003
N	7.60185347091866	3.78676455397448	2.04718502834834
N	8.46970888950114	1.49632410038032	3.75241172444673
C	6.45480530567268	1.14223042230640	-0.14395986965333
C	6.97955821475660	2.34227572739837	5.33908020363125
C	10.69708727750687	0.77598420680046	4.07400090381240
H	11.61510843739938	0.35821663638210	3.68656179518048
C	7.84676588223001	0.73049734301359	0.09997629593532
C	9.39157345616813	1.64894530164490	5.91509138673593
H	9.30138876476149	1.90902357358401	6.95937232683950
C	8.73298107624696	0.14096960842734	-0.79720112130543
H	8.43946669743852	-0.06323356664240	-1.81606029609265
C	9.46021892490633	0.64892205714073	1.81623652535540
C	10.00769734207290	-0.18466559804693	-0.35005390524935
H	10.71172256384189	-0.64333521557502	-1.03080376973174
C	8.32547860260044	1.82519907359517	5.03731657294456
C	10.57905629731866	1.12632371878386	5.41721772280825
H	11.41979099251685	0.98357726686210	6.08236923120883
C	4.42608890067423	3.26631655865887	5.68659319904289
H	3.40878691532082	3.61903824683006	5.77545554499914
C	9.60116739333517	0.97157934322748	3.24778459206811
C	4.90435441862791	2.86105553028493	4.44817080750402
H	4.26933446222408	2.90886037469644	3.57788260944326
C	1.16417440249425	0.12940328225569	4.62783778331044
C	10.38710826409488	0.06412564811334	0.96719311097979
H	11.37402911767106	-0.19791722177375	1.32031384326941
C	5.81734907292647	1.01019156449435	-1.36683945602874
H	6.34649046660536	0.59566854192677	-2.21195722081785
C	7.94408498533309	4.84978446145489	1.78475475296929
C	4.49104503154304	1.40303943453084	-1.48613658038844
H	3.97547168263048	1.30122405164575	-2.43141070299923
C	6.56918765409305	2.73566015363279	6.60289285965091
H	7.25146549751690	2.67690082338061	7.43833185026189
C	4.54411958347856	2.03110192108779	0.81610576345853
H	4.06415596577202	2.43375734003990	1.69358678538138
C	5.27199917507599	3.20073162687029	6.78119674123952
H	4.93330833957211	3.50653417746517	7.76187006379196
C	3.84162447985067	1.91705294300162	-0.37575319893009

H	2.80803909320269	2.22969590199864	-0.41714421497299
C	8.37261206116212	6.19308619374198	1.45535486632356
H	9.01574703728288	6.16666313152257	0.57512686223314
H	8.92591130068427	6.61591881546778	2.29458785995015
H	7.50104228160010	6.81473378118975	1.24763938994592
C	1.81371459873981	0.48271726945773	5.96140973133445
H	1.47229297075622	-0.19710027973004	6.74362808839595
H	1.54356633401472	1.50130507220479	6.24399986879015
H	2.89941535888329	0.42773310234454	5.89236765870851
C	-0.35776022525221	0.16178294554981	4.72448157688177
H	-0.81129583608184	-0.14073337536744	3.77956581954665
H	-0.68018956605966	1.17834497642686	4.95275752830472
H	-0.71054734623260	-0.49990082776142	5.51660322705709
C	1.66672159100402	1.02851411717832	3.50407743996853
H	2.74578361514340	0.94089270913485	3.39086563093464
H	1.43587884199251	2.06912961621234	3.73667713071894
H	1.18935472161847	0.77063318535818	2.55758658876897
C	3.12025909916815	-1.92752290630110	2.89623244556296
C	4.62666410082467	-1.95185952174987	2.76951837377420
H	5.08417095806323	-2.46634422980094	3.61347367212647
C	5.14650731303578	-2.47579765274268	1.46360512723631
C	6.15304692671361	-3.43908756388142	1.46161804369637
H	6.52182417923146	-3.82862540495728	2.40234291319842
C	4.68115310141201	-1.96362780052706	0.25167562665767
C	6.68679554675621	-3.89255591495581	0.26032662043945
H	3.91653742930268	-1.19809326983664	0.24118813489778
C	5.21919335924992	-2.41214467468697	-0.94704882548252
C	6.22208373206015	-3.37737635755495	-0.94520218494456
H	7.46893524827339	-4.64081660483013	0.26738453221776
H	4.86193838063292	-2.00140353899004	-1.88229150092545
H	6.64266617630935	-3.72309691223737	-1.88091129349033
H	2.68136549168938	-1.23769270179781	2.17439003811593
H	2.73128282596453	-2.93298429817504	2.71399200082951
O	6.32609446770965	-0.39981581672238	3.05634726740509
O	5.03271919246614	-0.52287519918452	2.89982947588880

Coordinates of VI (Final product) at at broken symmetry BS(3,1) triplet state

Co	6.84544182339782	1.82231996827391	2.59488328161080
O	2.69036735739106	-1.51178745160053	4.14660320527088
O	1.26802755869780	-1.23029710203540	4.18172410363308
N	8.20590583910986	0.83145736381323	1.35513878424781
N	5.74614212863463	1.47953496336700	0.81713885222863
N	6.20776512849602	2.44850394645243	4.52187597916164
N	7.41098737553223	3.74055375249095	2.00745056199793
N	8.49480853395803	1.40988152844939	3.80830451288463
C	6.45149679539934	0.96393658281360	-0.21217971190229
C	7.12432975270672	2.31565795992980	5.50483280400129
C	10.71994295592930	0.64459590120694	4.03153836382937
H	11.60731912377498	0.20102015929562	3.60433617638928
C	7.83631664971461	0.55820659983432	0.10723637155075
C	9.53909837354978	1.57846034593875	5.91786871437956
H	9.51129521221479	1.85950822572195	6.95990540700209
C	8.71720270440408	-0.08334810359809	-0.75946638814545
H	8.43203048358951	-0.32240458851397	-1.77246339723662
C	9.40474854868585	0.49812981522479	1.83801105129866
C	9.97449317429051	-0.42723479026143	-0.28334296858539
H	10.67578099354664	-0.93042788172736	-0.93512004831472
C	8.43407234529716	1.76246090529455	5.08973705428195
C	10.68613289779633	1.01966236661939	5.37036733389469
H	11.55850266746607	0.86933056048149	5.99193158670886
C	4.61616384727365	3.24899160276652	6.10535702059683
H	3.61412610643870	3.60418891942391	6.29834810818229
C	9.58127209010012	0.85522780298337	3.26532797665335
C	4.99015582808756	2.90253840142022	4.81539519169207
H	4.29382954075357	2.98631630912741	3.99151055648697
C	1.05631068492081	0.16396504918833	4.55808630939515
C	10.33444986257521	-0.14262250369468	1.02942894386086
H	11.30744813158168	-0.41954559042837	1.40798079836567
C	5.88313308298243	0.81414209410388	-1.46887908237389
H	6.45916769304868	0.40424893574412	-2.28434870555415
C	7.73027950854568	4.79272957758101	1.68350763502054
C	4.56064295682698	1.18531996986473	-1.66136701301950
H	4.10122463970911	1.07037538379636	-2.63377342951406
C	6.82310145424325	2.65371092333262	6.81714078641963
H	7.56363297476230	2.54333930244965	7.59518558916621
C	4.47212528028778	1.82354514288746	0.63290053385702
H	3.94731193141493	2.21843469816015	1.49279985889444
C	5.55278129600558	3.12481044598631	7.12003246537981
H	5.30058449889731	3.38659731375444	8.13888435450012
C	3.83625814246811	1.68881677534974	-0.59221133335953

H	2.80007622543086	1.97765482250441	-0.69519983197370
C	8.13622181017280	6.11835626130261	1.27428319993617
H	8.90835987952755	6.04130974384102	0.50800726431392
H	8.52925625532392	6.66107843379350	2.13459546830316
H	7.27561499666626	6.65219986910494	0.86949044144275
C	1.67449138462552	0.43653963417908	5.92505274895772
H	1.29871567634506	-0.27375870659004	6.66321743340691
H	1.41500223128800	1.44530725211035	6.24999971513625
H	2.76064552394435	0.36350457877290	5.88203803723919
C	-0.46664033606918	0.23678417555465	4.61107509744664
H	-0.89882922127171	-0.00652680916247	3.63938327254285
H	-0.76735461838811	1.25018274299934	4.87906783095375
H	-0.86235056220412	-0.45182883298035	5.35869654588416
C	1.61754448168445	1.10383377534308	3.49596141105657
H	2.70115458800910	1.02091604412566	3.43256391617347
H	1.37462778564652	2.13470958010330	3.75825290639123
H	1.18677386283466	0.88589393897799	2.51754155246309
C	3.02842755118105	-1.86012730722371	2.80901164999558
C	4.53751189147588	-1.81457648094196	2.74264925666187
H	4.98354324036719	-2.34539602962485	3.58267848397092
C	5.16292777124248	-2.22927192016968	1.44451244683014
C	6.45098998828780	-2.76602611223106	1.46242694381872
H	6.95151398833317	-2.92078875318959	2.40938805113035
C	4.52174998120780	-2.02892857839945	0.22309816548150
C	7.09149517854993	-3.09400058665419	0.27536275391988
H	3.53329054964456	-1.59228753538696	0.18625508009227
C	5.15847859437515	-2.37091339316809	-0.96314291644424
C	6.44427451221431	-2.89842564429842	-0.94017864618344
H	8.09508525711553	-3.49816964588261	0.29889630970987
H	4.65529158162042	-2.20770183871472	-1.90696985807965
H	6.94343520834037	-3.15048611625380	-1.86700496917186
H	2.58724693237297	-1.14636470690273	2.11363789032026
H	2.68118910749983	-2.86690729225364	2.56208820039773
O	6.11352814608389	-0.18195832974720	3.26798031291398
O	4.85202258201267	-0.37168781420608	2.98708564012360

Coordinates of VI (Final product) at quintet state

Co	6.85005569879886	1.83027576943762	2.62073360678505
O	2.75642208872255	-1.49591950279750	4.12893270566096
O	1.33618636237198	-1.21528943112966	4.21962039311103
N	8.23905692945296	0.85488914992244	1.38515516525123
N	5.79887957346404	1.55671301804162	0.79265502232625
N	6.17145233758536	2.40357574203109	4.54587439604609
N	7.41987141058316	3.77589846452392	2.10753405461619
N	8.47935449219381	1.39469336485628	3.85758053907356
C	6.50682768531578	0.99559403186411	-0.21214606660235
C	7.07597121646031	2.27521461322550	5.54216362241561
C	10.70663632127505	0.64196639546925	4.10667461051804
H	11.60436294014711	0.21058390968222	3.68836914905014
C	7.88234456939441	0.57470549285898	0.13364606016911
C	9.48984041166539	1.54609726857739	5.98576084893757
H	9.44428723933682	1.81420724741051	7.03076007478137
C	8.76816388972087	-0.08718632837129	-0.71415188693674
H	8.49515821343214	-0.33202401661417	-1.72932009841219
C	9.42518521118608	0.50506446080621	1.89062660229275
C	10.01511078414875	-0.44167971670724	-0.21719381607184
H	10.71967348603864	-0.95853474274174	-0.85463062862869
C	8.39570674894405	1.73235420650324	5.14296125784377
C	10.64975445903942	1.00200459139384	5.44937972490258
H	11.51393878605393	0.85211501464334	6.08248881102914
C	4.55739699405265	3.21083941531552	6.10305240014611
H	3.55327681842920	3.56821822766756	6.28054279018507
C	9.57817041050921	0.85257981546322	3.32422606305491
C	4.94905700919314	2.85921751609166	4.81963981279419
H	4.26509902074985	2.94118287123163	3.98521228080776
C	1.14040497554640	0.19078957152249	4.55884772644940
C	10.35885216288504	-0.15300113669606	1.09979092074110
H	11.32146113711426	-0.44324358395602	1.49492085472578
C	5.94780028477726	0.81615239350713	-1.47014196005112
H	6.52214021320727	0.36405003677490	-2.26441098480559
C	7.70223874606650	4.85130813543113	1.82851603409297
C	4.63582364873197	1.21101454849637	-1.69077207441037
H	4.18423104805502	1.07213564748946	-2.66381863027156
C	6.75317530170694	2.61334450637721	6.84979698294190
H	7.48201485870970	2.50342331328717	7.63910091578001
C	4.53661169683581	1.92790714454925	0.58063220192565
H	4.01137114771970	2.36015786675582	1.42242557803239
C	5.47799624612270	3.08578197089787	7.13255843407015
H	5.20997269498102	3.34914973789678	8.14699864659744
C	3.91267204965383	1.77077422045931	-0.64845600703513

H	2.88564737769996	2.08258652159092	-0.77483689757841
C	8.05821878485757	6.20713941963858	1.47601579242804
H	8.84361233645165	6.19160014623342	0.71939706567541
H	8.41622498070812	6.73272434274088	2.36191099841704
H	7.18175072722510	6.71986893088147	1.07779951364470
C	1.81425007424920	0.50509054109966	5.88994905648290
H	1.45987385809099	-0.17352976791131	6.66742987752227
H	1.57892720606977	1.52803364781719	6.18765741851033
H	2.89688862770090	0.41735753043587	5.80854046799411
C	-0.37886079647806	0.26991807215449	4.67227131097800
H	-0.85167543948145	0.00011233550418	3.72689596520217
H	-0.66582095296847	1.29172989989432	4.92301524536166
H	-0.74451574804317	-0.39513350043442	5.45566775407734
C	1.65751723068808	1.09423618566161	3.44412495691561
H	2.73625379372436	1.00169959959213	3.33113647047320
H	1.43380916015122	2.13419881766030	3.68688260538024
H	1.17883868492149	0.85098248479016	2.49430998087635
C	3.03351606610562	-1.89023749597583	2.79030892707188
C	4.53666231112146	-1.83491399988047	2.65011088910569
H	5.02910921543035	-2.32602404783297	3.48802914527505
C	5.10612321547141	-2.28675349027550	1.33751688550517
C	6.38017324975528	-2.85573627344282	1.31820801403888
H	6.90387629179336	-3.02609251464947	2.25027074353123
C	4.43878657123726	-2.05758627592269	0.13453627742253
C	6.98059497873666	-3.19137519458565	0.11154859118947
H	3.46330763530617	-1.59055562572970	0.12737236015680
C	5.03621495171259	-2.40428262122772	-1.07105299371425
C	6.30794581892079	-2.96662640308659	-1.08542802965234
H	7.97354732590291	-3.62189273082826	0.10569111261100
H	4.51491296798347	-2.21686786895602	-2.00062700003231
H	6.77657023354702	-3.22291323575834	-2.02703019394093
H	2.54995400667776	-1.20798682886186	2.09168706383776
H	2.68760501729086	-2.90920810930250	2.59649366361172
O	6.11660417359343	-0.19826619100488	3.11541978569864
O	4.86106874346433	-0.38340752147687	2.79606104199214

Coordinates of carbon-centered radical rebound cobalt product at singlet state

Co	6.77617304764005	2.61547468590431	2.57209278943247
O	3.71982212099689	2.31237196889775	3.63121056611061
O	2.32248127007965	2.49363356412076	3.27535825373022
N	7.48108447377501	1.67647103231643	1.11633249467795
N	5.43164223913915	3.16739888399215	1.18268150739702
N	6.50433048077341	3.45757112949119	4.37943488274753
N	7.84207940525631	4.26759212688088	2.06149418868719
N	8.26108661788385	1.88205418306836	3.43990957836193
C	5.69263342357149	2.61606009829085	-0.03262742854733
C	7.46982901837246	3.09062593267871	5.26400537286257
C	10.20395229187894	0.55192194193840	3.29367668679292
H	10.86873814882953	-0.07801371704526	2.72128711459992
C	6.87112766555366	1.74247330764160	-0.06326012666867
C	9.59161697207693	1.64071517978619	5.36333022628350
H	9.78910147349334	1.85548282458918	6.40273163479636
C	7.38180728271094	1.02620777142719	-1.14073491052290
H	6.89746980990128	1.05517812302720	-2.10524664029971
C	8.60083033952007	0.96673542405328	1.32897136813930
C	8.52642182294757	0.26575397565092	-0.94520110764726
H	8.93472245403473	-0.30589178152170	-1.76715106684561
C	8.48109107886892	2.17620860334437	4.71955173563338
C	10.44853983091136	0.82474836418178	4.63532237510295
H	11.31793292889659	0.39951493697270	5.11758969578386
C	5.54579760115555	4.86324506681340	6.04923812709325
H	4.76639965227857	5.56364656691870	6.31206349479581
C	9.07486184663240	1.10261400142939	2.71065228299280
C	5.57622236594816	4.32605522045893	4.77046069072878
H	4.82500968053818	4.59541534954336	4.04931831691069
C	1.51003843776596	2.49927302600648	4.48147014806902
C	9.15641050817995	0.23273638155253	0.29521149941831
H	10.05264274970608	-0.34933656768325	0.45146531836212
C	4.90566235953645	2.88599374108799	-1.13914100639050
H	5.13267371674880	2.42810487886438	-2.09046117785879
C	8.44600299031860	5.19938784838269	1.78234700251814
C	3.82990710862261	3.75293327974557	-1.00732711131358
H	3.20125304272474	3.97468922893299	-1.85893083434936
C	7.49271417450828	3.57911373748350	6.55981812141317
H	8.26871135931144	3.26879780908398	7.24362811498704
C	4.40866848269157	4.01092969369839	1.29474220907627
H	4.22648211495495	4.43265857789923	2.26724528083100
C	6.51412320984763	4.47765362534679	6.96057826499543
H	6.51535050892266	4.87152562659950	7.96778633142099
C	3.58287981288776	4.33017890601857	0.22663855240640

H	2.76275072085804	5.01718434424335	0.37724730206455
C	9.20603864856362	6.37851150709495	1.43241702451090
H	9.48676281037156	6.33121738141781	0.37973921060755
H	10.10545670689795	6.42633428847038	2.04716704791451
H	8.59847045226161	7.26707829206426	1.60720540815218
C	1.60192804991365	1.15396033406604	5.19281325263874
H	1.29804356182783	0.34527280478084	4.52660446853772
H	0.94409013301141	1.15163915004724	6.06332758176555
H	2.61939718223827	0.96679078002912	5.53607245176082
C	0.11329437723915	2.73338734573329	3.91368276643595
H	0.06640070901364	3.68544686388386	3.38316334202148
H	-0.60791186449244	2.75654543751905	4.73128365521562
H	-0.16467159668344	1.93104632976287	3.22884189267987
C	1.93507644449255	3.64591288081433	5.39239042407141
H	2.93267471264115	3.47344982922363	5.79299902411366
H	1.24070485960457	3.72946456321772	6.22992028321853
H	1.93315351452104	4.58948330803910	4.84414250007426
C	4.16900033928036	1.14921770201817	2.94826638643807
C	5.65479570143820	0.99472360731248	3.21708255995560
C	6.16324194324596	-0.32214438580493	2.74773196361551
C	7.06028308880321	-1.04385723423589	3.54340796325173
H	7.34795910527887	-0.64450388204089	4.50824679806737
C	5.78698255394750	-0.88170056664804	1.51944118288740
C	7.59239494480166	-2.25357130775378	3.11763126083077
H	5.08457707659089	-0.36480386832604	0.87997794359736
C	6.31695453520264	-2.08960531735432	1.09020238469928
C	7.23031062841189	-2.77892107573002	1.88264217998246
H	8.29319378025618	-2.78293552052453	3.75090679239849
H	6.02055373729432	-2.49180269980996	0.12959329335829
H	7.64954708119390	-3.71735109750478	1.54303419371479
H	3.93215260737062	1.23531750284017	1.88695928265977
H	3.64100515982561	0.27188842223872	3.33903255731725
H	5.81712083662904	1.06958567621313	4.29034283630712

Coordinates of carbon-centered radical rebound cobalt product at triplet state

Co	6.59067611350896	2.62905710283903	2.65071221707121
O	3.71267845719087	2.27846776230335	3.69854826436665
O	2.35791634497392	2.64017311939050	3.32323445575758
N	7.57040393129256	1.64297793073753	1.08248072766233
N	5.32915021757030	2.95327862051750	0.98494403735133
N	6.57491876706757	3.37354680453749	4.62679749952871
N	7.49184753602990	4.48645036798213	2.12651744143404
N	8.34264718314550	1.85449500560396	3.48797974335778
C	5.74372330350612	2.41769389656014	-0.18312080170312
C	7.59272102202238	2.97524172939226	5.41880959559608
C	10.29966515493260	0.54300399713730	3.27744746039000
H	10.96417059161648	-0.05908778540453	2.67574993953363
C	6.99437809945799	1.63455017125408	-0.11522918993181
C	9.70738546777429	1.54041452501959	5.38999232961946
H	9.91507979312721	1.71362349469320	6.43499616750098
C	7.56714845721644	0.92317509875351	-1.16608852826713
H	7.11072906924796	0.90214547516866	-2.14416506881049
C	8.70040566207306	0.97964045549474	1.33451719594816
C	8.74103028306195	0.22437688958985	-0.92451805470338
H	9.20136823557035	-0.34415978579673	-1.72112044355646
C	8.58620165060212	2.09103213688241	4.77454386496885
C	10.56249942258062	0.75978393957037	4.62429625337646
H	11.43998107599208	0.32031246939853	5.07897867313446
C	5.71377473084435	4.71205729726668	6.39843355860814
H	4.95443488222011	5.40000896346153	6.74149106556110
C	9.15694323148329	1.11327184993180	2.73332175688448
C	5.66784245775951	4.22590349200626	5.10080699170580
H	4.88050209577912	4.51835956931544	4.42173648909650
C	1.52029432782332	2.75300272275238	4.50982974296297
C	9.32372462664489	0.24300612908262	0.33679526349852
H	10.23223408113747	-0.30681529779310	0.53222303524358
C	5.03589385244319	2.62065515568511	-1.35950699991739
H	5.37727775855624	2.18204261454518	-2.28502314828244
C	8.00824542126712	5.47366411815643	1.85751528085493
C	3.88986323729308	3.40254861578102	-1.33317451088489
H	3.32640989633863	3.57098159725907	-2.24108469321960
C	7.69582291608947	3.41365178252379	6.73207887235892
H	8.51287461401262	3.08601735855340	7.35717559441219
C	4.23648670980603	3.71678418180815	1.00471046778562
H	3.94789537181652	4.12096272706080	1.96334753600497
C	6.74136123776953	4.29016181283691	7.22828814212466
H	6.80686637193828	4.64290801867195	8.24875714437427
C	3.48541034116955	3.96750005645347	-0.13345857998673

H	2.60380729703160	4.58901902864296	-0.06831299769466
C	8.66257580631238	6.71965460830166	1.52143033592790
H	8.84455433668875	6.75847148604526	0.44700175428574
H	9.61256516020511	6.78605530978633	2.05274434505031
H	8.02597078136760	7.55599447341620	1.81186375436928
C	1.40468467235777	1.40796846390243	5.21772718114743
H	1.01614568228393	0.64611712123534	4.54047099424239
H	0.72158318697993	1.49996445940341	6.06345364511345
H	2.37304371602340	1.08508919839648	5.60052616025725
C	0.18782632611312	3.17583205274979	3.89750029084031
H	0.28714232249948	4.12819782841441	3.37505757464544
H	-0.55002787019538	3.29123466985125	4.69200837401849
H	-0.17260271709858	2.42163839747237	3.19667305170874
C	2.06699033600579	3.82881139078650	5.44104778381235
H	3.00867394630662	3.51360489523291	5.88688219947956
H	1.35422198947322	4.01201832934907	6.24637465728892
H	2.22592489425184	4.76155226839334	4.89758848748192
C	3.98740381470534	1.01895007050894	3.09233145680274
C	5.41414399629130	0.69356317001321	3.39735826826265
C	6.04339910059883	-0.44067850941667	2.78029818168541
C	7.08290898256903	-1.10651830860068	3.46207731116121
H	7.37064614402937	-0.75867611754952	4.44595790712161
C	5.67352331224628	-0.91731450874717	1.50532778992177
C	7.73006738341253	-2.18692649076453	2.89243388072489
H	4.88644669664153	-0.42428946176194	0.95149224622140
C	6.33066530002091	-1.99210370344727	0.93702651236437
C	7.36344160343941	-2.63067097363296	1.62347301759436
H	8.52864122810053	-2.68040552976373	3.43094476849668
H	6.04704394162339	-2.33251607952439	-0.05031570020396
H	7.88024897601656	-3.46638518541000	1.16992828416701
H	3.78091849419319	1.06865477417124	2.02329676478054
H	3.34160189155227	0.24620548476486	3.52770249270154
H	5.69777230432683	0.87204066475690	4.42893866017475

Coordinates of carbon-centered radical rebound cobalt product at broken-symmetry (3:1) triplet state

Co	6.59094387208842	2.62903372329112	2.65135043085561
O	3.71320624226778	2.27858512599989	3.69850053390494
O	2.35869610396365	2.64091332288756	3.32286745234449
N	7.57058380676473	1.64314104687859	1.08285691137983
N	5.32955029745063	2.95387016863985	0.98544292527107
N	6.57535948232875	3.37320461918617	4.62743057472508
N	7.49239171607654	4.48644267679989	2.12711141520333
N	8.34269980455156	1.85388769321073	3.48846639428373
C	5.74407284582278	2.41837597634906	-0.18268880106810
C	7.59293854843923	2.97441158726594	5.41949839647080
C	10.29970324787999	0.54241485555680	3.27762470606090
H	10.96419280718742	-0.05955995012332	2.67579606598554
C	6.99452302424543	1.63491713217810	-0.11484190163972
C	9.70752664038515	1.53947333977136	5.39036319969031
H	9.91529225004379	1.71248666107520	6.43538782239544
C	7.56706299665391	0.92343099174264	-1.16574856908838
H	7.11059612867235	0.90257545317210	-2.14380673564431
C	8.70040817165341	0.97946023656320	1.33482631739092
C	8.74075783687383	0.22429634261571	-0.92425129236450
H	9.20090149111478	-0.34434723518651	-1.72088931159425
C	8.58633931298729	2.09023810709917	4.77506391042460
C	10.56257644104982	0.75894209484385	4.62450144988961
H	11.44007789884508	0.31938893741133	5.07906218758087
C	5.71410329895860	4.71118133095584	6.39942919566917
H	4.95482061252359	5.39913155763185	6.74263243404168
C	9.15696303382462	1.11280337618774	2.73363070065153
C	5.66831594887718	4.22553281306484	5.10159727119159
H	4.88113944858067	4.51838455326542	4.42249660115486
C	1.52056810724367	2.75322567575857	4.50916691889785
C	9.32350898866117	0.24271522807557	0.33704105842733
H	10.23187886487167	-0.30736251039484	0.53241432294337
C	5.03636610006506	2.62169629781088	-1.35908541936661
H	5.37771706310078	2.18316776700655	-2.28465447230464
C	8.00730259382209	5.47415066702516	1.85706087969737
C	3.89049228210274	3.40381510716276	-1.33268557703339
H	3.32712812810834	3.57250831678191	-2.24059916381851
C	7.69588395624828	3.41230004730308	6.73295939537509
H	8.51276299242517	3.08427613004817	7.35810261983313
C	4.23706756634577	3.71764672100315	1.00525957393177
H	3.94852246884525	4.12179591361461	1.96392684123804
C	6.74146431846668	4.28877664474628	7.22932393403868

H	6.80683257819632	4.64111249769148	8.24995066897805
C	3.48610232147645	3.96868001296200	-0.13290821571450
H	2.60461850840533	4.59035842738983	-0.06771181011513
C	8.65961941954670	6.72080864819049	1.51952786310317
H	8.82218089339689	6.76811425119591	0.44233139600988
H	9.61929947797193	6.77979113027529	2.03400809824588
H	8.03112995717650	7.55664100072442	1.82844399321750
C	1.40378341607348	1.40764907197333	5.21582125248776
H	1.01524538372988	0.64662844887751	4.53765651232676
H	0.72022819204675	1.49929024791641	6.06122130309101
H	2.37172496154086	1.08389827297969	5.59892682964612
C	0.18864528500237	3.17737538045021	3.89653904777552
H	0.28873633679529	4.13022657488318	3.37514759303823
H	-0.54957663992761	3.29230649966567	4.69076455915464
H	-0.17178756375519	2.42409987900835	3.19473507535517
C	2.06743623141885	3.82790728209427	5.44157137984346
H	3.00879460871936	3.51184472084668	5.88748360855294
H	1.35443170444993	4.01071651494116	6.24676231980833
H	2.22702473451865	4.76107533519813	4.89902742506728
C	3.98785898583026	1.01918559757788	3.09203118233961
C	5.41451750808610	0.69353673119972	3.39733903351403
C	6.04364921347766	-0.44080974889953	2.78029750025224
C	7.08280758425520	-1.10696747947890	3.46227583631042
H	7.37039288534711	-0.75927358807861	4.44625309273435
C	5.67396924009749	-0.91721607542707	1.50520267273185
C	7.72985686418239	-2.18746408160372	2.89266074444270
H	4.88713444870358	-0.42395075526715	0.95125533705631
C	6.33100532640009	-1.99206985462670	0.93692153452947
C	7.36344960826172	-2.63096200248603	1.62355313422054
H	8.52816536017124	-2.68119711374480	3.43130795355344
H	6.04756717415814	-2.33228629530710	-0.05054084577603
H	7.88018279029717	-3.46673907861778	1.17002743907514
H	3.78163083409976	1.06916506244810	2.02296006450007
H	3.34185015767464	0.24641548407005	3.52707290953577
H	5.69792250988353	0.87179992066110	4.42901853684420

Coordinates of carbon-centered radical rebound cobalt product at quintet state

Co	7.08163481657653	2.66808554042151	2.45914004346118
O	3.11494767555206	2.17557889433987	3.81002783858199
O	1.80476809174599	2.58532198871721	3.32176580838490
N	7.96740802904825	1.64630608205120	0.87420029882383
N	5.58543846467239	2.67566888168290	0.99056286769910
N	6.85318939511884	2.89938736435406	4.52841742655346
N	7.84508855839297	4.53620646603107	2.07312203929460
N	8.82747787079068	1.82585467270070	3.23541416968707
C	5.93230643926217	2.13484655519241	-0.20042502039516
C	7.91162646108934	2.52721717899313	5.28387265796969
C	10.88708110921754	0.71964901497335	2.89456943276920
H	11.59561510355281	0.23842581640680	2.23677926606694
C	7.29254345693794	1.56185502175111	-0.26770837682505
C	10.20871354927866	1.41494121279257	5.10317346145509
H	10.39442780795138	1.47283065037890	6.16496141884489
C	7.86654168721356	0.94545517236526	-1.37610036926501
H	7.33211096939807	0.85967100418543	-2.30994518424614
C	9.18713858989482	1.12714303972021	1.02531528307865
C	9.14890795922271	0.42854729214573	-1.25073731976711
H	9.61776418284520	-0.05346392503407	-2.09789604092922
C	9.03501062688335	1.91425539263497	4.54483149270558
C	11.13928234063820	0.82565122699100	4.25791981219339
H	12.05970719486298	0.43074125645108	4.66623449422797
C	5.71598652282460	3.63961029200253	6.48877073538183
H	4.82902005879252	4.07945827343202	6.92166263445380
C	9.69151442181167	1.23161743094209	2.41128693737542
C	5.78711489582512	3.44232825168189	5.11828027656693
H	4.96090803175685	3.71023441186482	4.47666687980559
C	1.02574157498715	3.12471846221792	4.42327751556908
C	9.82625117665859	0.50785934229647	-0.03950170282118
H	10.81393621216118	0.08514105246423	0.07031571637572
C	5.04436985276548	2.11057679818291	-1.26455212105265
H	5.33343917758482	1.67304968282961	-2.20830023667715
C	8.25740874789940	5.58407114457346	1.86016352854518
C	3.77546858266341	2.65011523136172	-1.10092564633866
H	3.06916843255774	2.63295088179675	-1.92002616653858
C	7.90710019486003	2.69454860520554	6.66067670602702
H	8.75742864244646	2.38790546410270	7.25103680631566
C	4.37026026163440	3.20481334478251	1.13949695740081
H	4.13574321876482	3.62008613610879	2.10777369555682
C	6.79467132256945	3.25786374207395	7.27068180385508
H	6.77488591899806	3.39426030429229	8.34358168433087
C	3.43070445601656	3.20785124779205	0.11951138960377

H	2.45481103063914	3.63731377864724	0.29296887461633
C	8.77870541033285	6.90430373543911	1.59102899017964
H	8.98420936276981	7.00366008137278	0.52463036479288
H	9.69930265661622	7.05376210938030	2.15626756503390
H	8.04277002605841	7.65138909338142	1.89088822840672
C	0.79270382116355	2.05700766767185	5.48682702731695
H	0.29570141588284	1.18650166042581	5.05585541641918
H	0.15936712097243	2.46013125382103	6.27863212317244
H	1.73580842447758	1.74227232772408	5.93327036139120
C	-0.27732527346702	3.50672007793745	3.72676997888608
H	-0.09582289256844	4.24381773060031	2.94325137727264
H	-0.96144169641976	3.93942740247784	4.45750767721659
H	-0.75115175822375	2.62875902815635	3.28562245809182
C	1.72107604104514	4.35297445033449	5.00192045434639
H	2.65550029239956	4.07717249238362	5.48857893824492
H	1.07700575659001	4.82594476252941	5.74476745978367
H	1.93342879911920	5.07735839217164	4.21381064613110
C	3.28096339499850	0.80158767209785	3.43251373386089
C	4.61418095132229	0.38591087469145	3.93025521199358
C	5.60200271256133	-0.29053207190047	3.18775504797962
C	6.80096864422790	-0.69706756061244	3.83734319579848
H	6.93286745742899	-0.44792158707960	4.88293088610958
C	5.46237026930358	-0.62733469957497	1.81358486998308
C	7.78068093236898	-1.39475524694625	3.16306054989979
H	4.57513770584998	-0.32701530816717	1.27455857119873
C	6.45062813974540	-1.32757589566482	1.15117169089164
C	7.61604892706293	-1.71733491722771	1.81400726835531
H	8.68689257701486	-1.68253164640215	3.68056128177460
H	6.32331143251288	-1.56522381624420	0.10285732146082
H	8.38968987672125	-2.25607097034209	1.28352199187202
H	3.18373996967099	0.70987451497882	2.35021732074521
H	2.48527713243574	0.20959620133464	3.90115683854535
H	4.81737340647316	0.58299356506191	4.97616919886507
